# Urbanization processes widen global divergence in extreme temperature variability

**DOI:** 10.1038/s41467-026-75457-z

**Published:** 2026-07-08

**Authors:** Renlu Qiao, Fangzheng Li, Tao Wu, Zeyin Chen, Xiang AO, Xi Meng, Jingkai Zhao, Zhiqiang Wu, Borong Lin, Yue Zhang

**Affiliations:** 1 Key Laboratory of Eco Planning & Green Building, Ministry of Education, Tsinghua University, Beijing, 100084 China; 2 Beijing Key Laboratory of Smart Spatial Planning for the Capital Metropolitan Area, Tsinghua University, Beijing, 100084 China; 3 Department of Urban Planning, School of Architecture, Tsinghua University, Beijing, 100084 China; 4 School of Landscape Architecture, Beijing Forestry University, Beijing, 100083 China; 5 Shanghai Research Institute for Intelligent Autonomous Systems, Tongji University, 1239 Siping Road, Shanghai, China; 6 College of Architecture and Urban Planning, Tongji University, 1239 Siping Road, Shanghai, China; 7 School of Geography and the Environment, University of Oxford, South Parks Road, Oxford, OX1 3QY United Kingdom; 8 Faculty of Information Technology, Beijing University of Technology, Beijing, China; 9 Department of Building Science, School of Architecture, Tsinghua University, Beijing, 100084 China

**Keywords:** Climate-change mitigation, Environmental impact, Climate sciences

## Abstract

Extreme temperature variability (ETV) is a key dimension of climate risk for billions of residents. However, how urbanization shapes global ETV divergence remains unclear. Here, we assemble a 1950–2020 panel of 10,522 cities and demonstrate that ETV trajectories diverge by development status as measured by the human development index (HDI). ETV intensifies in high-development cities, whereas it slowly weakens in low-development cities, resulting in a current gap of roughly 1.66 °C. Decomposing ETV into event frequency and intensity reveals that cumulative ETV is driven mainly by intensity. Using an interpretable machine-learning framework, we find that aerosols are the urban factor most strongly associated with ETV after controlling for climate and geography. Blue-green space is consistently associated with lower ETV, whereas urban morphology has a smaller and context-dependent effect. These findings link global ETV inequality to urban governance and support targeted management that focusses on limiting volatility under climate risk.

## Abstract (web summary)

Analyzing temperature data from 10,522 cities globally from 1950–2020, this study shows that urban extreme temperature variability diverges by development status, driven mainly by event intensity, and is linked to aerosols, blue-green space, and urban form.

## Introduction

Global climate change and rapid urbanization are reshaping daily life worldwide. Human-driven warming not only raises average temperatures but also shifts temperature variability and the frequency and intensity of extremes^1^. Short-term temperature variability (TV) is now recognized as a critical risk, as people and systems have little time to adapt^2,3^. Recent evidence further suggests that abrupt day-to-day temperature swings constitute a distinct hazard dimension that is largely independent of conventional ETCCDI temperature-extreme indices across almost all global land areas^4^. Sudden swings in temperature strain health services, disrupt energy demand, and stress ecosystems^5–7^. The World Health Organization (WHO) reports that frequent temperature fluctuations exacerbate heat stress and heat-related illnesses, thereby increasing the risk for urban residents. Urban TV, therefore, matters for public health, reliable energy systems, and ecological stability. It also matters for progress toward the United Nations’ Sustainable Development Goals (SDGs), specifically those related to sustainable cities and human well-being.

Urban areas sit at the frontline of climate risk. Cities concentrate people, infrastructure, and economic activity, and their distinct microclimates (for example, urban heat islands) can amplify heat exposure^8^. Observations show that, in recent decades, urban centers have warmed about 29% faster than nearby rural areas^9^. Most studies emphasize macro-scale indicators, such as the differences in mean temperature between urban and suburban areas and the frequency and intensity of extreme events. In contrast, knowledge of urban temperature variability, including abrupt day-to-day fluctuations and changes, remains limited^6,7,10^. Conventional temperature-extreme metrics are primarily designed to characterize exceedance, duration, or annual maxima of sustained thermal extremes. They are therefore highly informative for chronic warming and heatwave exposure, but less suited to capturing abrupt day-to-day thermal volatility and the cumulative load generated by repeated temperature swings. ETV is intended to complement, rather than replace, these metrics by quantifying the structure of thermal fluctuation itself, thereby identifying risk associated with rapid temperature changes even where persistent extreme conditions are less pronounced. Recent work also shows that land use change, such as deforestation, can increase daily temperature variability by up to 20% in certain regions, resulting in more frequent and rapid temperature swings, both warm and cold. However, whether the dense and thermally complex fabric of cities dampens or magnifies such abrupt variability is still unclear and calls for direct investigation.

Cities interact with both the radiative and dynamical components of the surface energy balance^11^. Low-albedo materials, large volumetric heat capacities, and canyon-scale radiative trapping alter net shortwave and longwave exchange, thereby increasing heat storage. Increased surface roughness and anthropogenic heat release generate local circulations, modifying the boundary layer stability^12–15^. These flows also interact with synoptic advection and passing fronts. The coupled pathways can buffer day-to-day variability by slowing nocturnal cooling and shortening post-event decay. Under certain atmospheric conditions, the same pathways can combine to produce compound responses and sharper temperature fluctuations^16^. The sign and magnitude of urban TV, therefore, cannot be inferred from isolated case studies. A global, integrative perspective is necessary to understand how short-term variability responds to the combined effects of city morphology, land-cover mosaics, and the climatic background.

Here, we show a global, city-level assessment of extreme temperature variability (ETV), defined as day-to-day differences in near-surface air temperature (see Methods). We compile ERA5-Land daily data from ECMWF and track 10,522 cities from 1950 to 2020. We harmonize these records with climatic, geographic, urban-form, and socioeconomic covariates. We demonstrate that atmospheric circulation, land–sea thermal contrasts, and development levels collectively influence the spatial pattern and temporal evolution of ETV. For 2005–2019, when annual urban covariates are available, we integrate climatic, geographic, urban-form, and socioeconomic indicators and apply an interpretable machine-learning framework to assess the associations between city-level factors and ETV across climate zones and development stages. We show that ETV trajectories diverge by city development status, and that recent cumulative ETV is driven more by event intensity than by event frequency. Those urban features, such as aerosols, blue–green infrastructure, and surface properties, are associated with distinct dimensions of ETV. Together, these findings position ETV as an underexamined axis of urban climate-risk inequality and advance Sustainable Development Goal 11 and climate–health adaptation targets.

## Results

### ETV’s spatio-temporal change trends

**Fig. 1:**
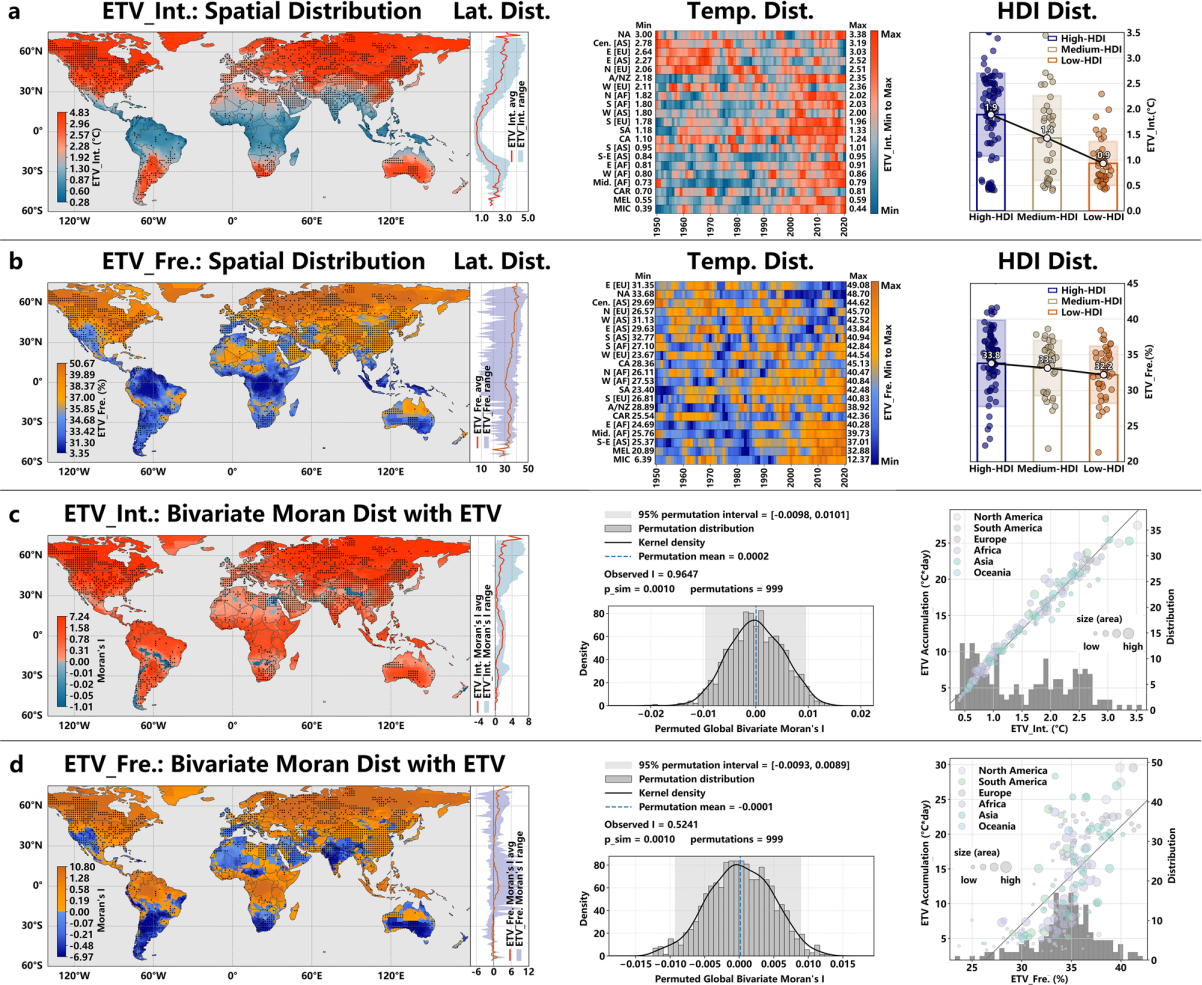
Spatio-temporal distribution of extreme temperature variability and its intensity and frequency components. **a**, **b** Spatial distributions of single-event ETV intensity (ETV_Int.; °C) and event frequency (ETV_Fre.; %) across 10,522 cities during 1950–2020, together with their latitudinal distributions, temporal evolution by geographic subregion, and distributions across Human Development Index (HDI) groups. In the HDI panels, points show annual group-level values, and the black line connects group means. **c**, **d** Spatial correspondence of annual cumulative ETV with ETV_Int. and ETV_Fre., respectively. The maps show local bivariate Moran’s I. The middle panels show the null distributions of global bivariate Moran’s I derived from 999 permutations. The right-hand panels relate ETV_Int. or ETV_Fre. to annual cumulative ETV at the city level. Point colors denote continents, point size scales with city built-up area, the solid gray line shows the fitted relationship, and gray bars show the marginal distribution of the corresponding ETV component. Regional abbreviations are as follows: NA Northern America; Cen. AS Central Asia, E EU Eastern Europe, E AS Eastern Asia, N EU Northern Europe, A/NZ Australia and New Zealand, W EU Western Europe, N AF Northern Africa, S AF Southern Africa, W AS Western Asia, S EU Southern Europe, SA South America, CA Central America, S AS Southern Asia, S-E AS South-Eastern Asia, E AF Eastern Africa, W AF Western Africa, Mid. AF Middle Africa, CAR Caribbean, MEL Melanesia, and MIC Micronesia.

Over the past 70 years, observations show a global annual mean ETV of 18.65 ± 9.36 °C. ETV increases in a clear three-step pattern with latitude (Fig. 1). This gradient reflects the dominant roles of large-scale circulation and the ocean–land contrast. It also signals a local reshaping of energy and water balances due to urbanization. At low latitudes (0–30°), the mean ETV is 10.83 ± 7.37 °C. Year-round humidity and evapotranspiration sustain large latent heat fluxes^17^. The high tropical tropopause further damps day-to-day swings. Coastal South Asia, Southeast Asia, and equatorial Africa, therefore, show minimal variability. Subtropical dry interiors, such as central Australia, southwestern Africa, and the far south of South America, also remain relatively stable. At mid-latitudes (30–60°), planetary waves have larger amplitudes, and jet-stream meanders frequently replace warm and cold air masses^11,18^. Mean ETV nearly doubles to 20.72 ± 8.56 °C. Distinct variability arcs appear from the Great Lakes to the U.S. East Coast, across the European plains, and along the North China–Korea–Japan corridor. At high latitudes (above 60°), jet-stream meandering and blocking occur more frequently. Polar amplification magnifies radiative perturbations^3^. Mean ETV rises to 25.49 ± 11.27 °C. Alaska, northern Canada, Scandinavia, and Siberia form the most concentrated high-ETV belt worldwide.

Coastal cities within 100 km of the shoreline show a much lower annual ETV (14.96 ± 7.80 °C) than inland cities (21.23 ± 9.49 °C). This coastal signal is not tied to any single basin; it forms a near-continuous belt along global shorelines. Lower variability is especially evident along the mid- to high-latitude coasts of the North Pacific and North Atlantic, around the Mediterranean, and along the southwestern margins of Africa and South America. At the same latitude, moving inland typically raises ETV by 5.15–5.44 °C, implying that ocean–land coupling offsets roughly 24–62% of urban temperature fluctuations. The ocean’s high heat capacity, which stores heat during the day and releases it at night, together with daily land–sea breezes that bring cooler, humid marine air inland, markedly dampens day-to-day variability in coastal cities^19^.

We decompose the annual ETV (1950–2020) into two metrics, event frequency (the number of variability days per year) and event intensity (the magnitude of each variability). To identify which component dominates the annual cumulative ETV pattern, we compare its spatial correspondence with the frequency and intensity fields using bivariate Moran’s I. The result shows that the spatial pattern of cumulative ETV mirrors the intensity field (Moran’s I 0.96) and differs markedly from the frequency field (Moran’s I 0.53). In the high-latitude belt, the number of variability days changes little, but larger per-event swings push annual totals to global maxima. Many low-latitude coastal and humid green-belt cities exhibit modest increases in variability days, yet maintain low cumulative loads because evapotranspiration and oceanic heat capacity mitigate the amplitudes^19^. This means that similar annual ETV loads can arise from very different event structures, but in intensity-dominant regimes, risk is concentrated in a relatively small number of high-amplitude events. Such events can impose disproportionate burdens on health, energy systems, and infrastructure. Focusing on event amplitude, therefore, provides a more relevant basis for early warning, design thresholds, and risk management than event counts alone. Reducing the upper tail of event amplitude may be especially important in high-latitude inland cities, where cumulative ETV is most strongly concentrated.

Together, these results suggest that urban heat risk over the past 75 years has been intensity-driven, rather than frequency-driven. This means that annual ETV can be shaped by a relatively small number of severe events, which may impose disproportionate burdens on health, energy systems, and infrastructure. Focusing on event amplitude, therefore, provides a more relevant basis for early warning, design thresholds, and risk management than event counts alone. By compressing the high-intensity tail, cities can reduce annual heat-variability loads and prevent risk from accumulating in high-latitude inland regions.

Since the 21st century, the annual mean intensity of ETV across global built-up areas has decreased from approximately 2.30 °C to 2.27 °C. Over the same period, variance across cities increased from 0.85 to 0.95, indicating a shrinking mean but widening dispersion. Most cities exhibit modest convergence, characterized by low and stable amplitudes. A smaller set of cities, especially those with high HDI, inland locations, and cold or arid high latitudes, continues to intensify, driving the spread. In low-HDI cities (HDI < 0.60), total ETV intensity decreased by more than 0.12 °C. In high-HDI cities (HDI ≥ 0.80), the overall level is higher, increasing by about 0.08 °C. The gap between low- and high-HDI cities is now close to 1.66 °C. This pattern marks a shift from a broad concern to a structural problem concentrated in specific urban clusters. Adaptation resources should therefore target hotspot cities with surging variability rather than the reassuring trend in the global mean.

As a robustness check, we recomputed ETV using daily near-surface air temperature from the CMIP6 EC-Earth3 historical simulations, using the exact ETV definition and urban spatial units as in the ERA5-Land analysis. The EC-Earth3 fields reproduce the coastal–inland contrast and the intensification of variability in high-latitude inland regions, indicating that these patterns are not specific to a single data product (Supplementary Fig. 8). Spatial concordance between ERA5-Land and EC-Earth3 is high: the global bivariate Moran’s I between the two datasets reaches 0.95 for event intensity (ETV_Int.) and 0.55 for event frequency (ETV_Fre.), demonstrating that our conclusions about an intensity-driven, spatially divergent evolution of urban ETV are robust.

### Urban factors associated with ETV

The overall magnitude of urban ETV has decreased, yet the gaps between cities have widened. Climate and geography account for much of the baseline spatial pattern, but they cannot explain this pattern of “mean convergence with stretched extremes.” Urbanization processes are driving the divergence^16^. The pace of surface sealing, the strength of sensible heat emissions, the extent of blue–green infrastructure, and block-scale morphology that governs ventilation and heat storage are reshaping local ETV trends^20–22^. To assess whether city-level urban characteristics are associated with this divergence, we developed an interpretable machine-learning framework for the 2005–2019 period using 10,522 cities with built-up areas exceeding 10 km², the sample for which annual urban descriptors are available. After controlling for macro-climatic and geographic background, five urban factors, including aerosol concentration, anthropogenic heat, urban morphology (in Supplementary Table. 1), blue–green infrastructure, and surface albedo, explain 30.83% of the spatiotemporal variation in ETV intensity and 48.85% in ETV frequency. The models achieve R² values of 0.97 for intensity and 0.84 for frequency. Crucially, the association patterns of these factors differ significantly between intensity and frequency, indicating distinct levers for risk management. The association patterns of climatic and geographic background, sensitivity analyses based on GAMs, and 3/7-day rolling windows are provided in Supplementary Fig. 1, Supplementary Fig. 2, Supplementary Fig. 3, Supplementary Fig. 4, Supplementary Fig. 5, Supplementary Fig. 6, and Supplementary Fig. 7.

**Fig. 2:**
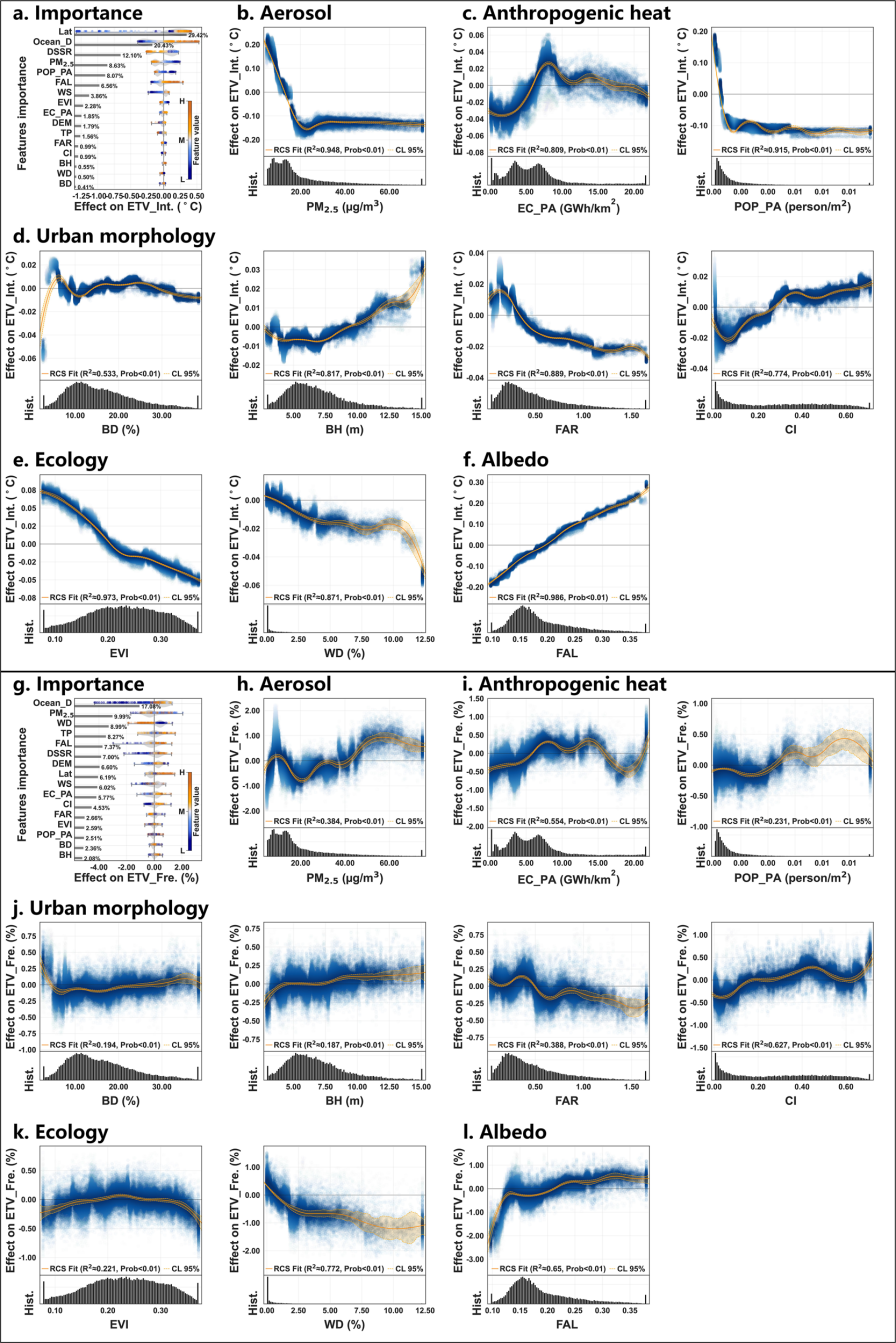
The effect of urban factors on extreme temperature variability (ETV). **a**, **g** Global importance and local effect of each feature included in ensemble learning models on ETV intensity (ETV_Int) and event frequency (ETV_Fre.) (*n* = 157,830). **b**–**f**, **h**–**l** the X-axis shows the sample feature value, and the Y-axis shows the SHAP value of the sample feature, i.e., the local effect on ETV. The Global importance of each feature is expressed as the ratio of each feature’s mean absolute SHAP value to its total value. Abbreviated in the figure as: electricity consumption per area (EC_PA), population per area (POP_PA), building density (BD), building height (BH), floor area ratio (FAR), compactness index (CI), enhanced vegetation index (EVI), water density (WD), and forecast albedo (FAL).

For decades, blue–green infrastructure (BGI) has been viewed as the primary means of regulating urban heat through evapotranspiration and increased reflectance^23^. However, it is worth noting that aerosol concentration (PM₂.₅) is the strongest predictor of ETV intensity in our models, with a marginal contribution of 8.63%, about four times that of ecological variables (Fig. 2). Mechanistically, the response is nonlinear. At low to moderate PM₂.₅, shortwave scattering lowers daytime absorption and damps single-event amplitudes. At higher concentrations, especially where anthropogenic heat is substantial, enhanced nighttime longwave re-emission and greater stability increase near-surface thermal inertia, allowing warm anomalies to decay more slowly and intensify. The relationship features a pivot near ~20 µg m⁻³, beyond which damping saturates or reverses. BGI remains consistently negatively associated with ETV intensity, but its overall weight is modest. Anthropogenic heat, proxied by population density and electricity use, ranks next to PM₂.₅ in terms of its direction and shape of effect: high population and energy demand add sensible heat and often co-occur with emissions, thereby strengthening thermal inertia. Surface albedo contributes 6.56% and shows an approximately linear positive association with intensity. Urban morphology has a smaller total contribution (2.94%) but mixed signs: higher FAR correlates negatively with intensity, likely because denser blocks have greater effective heat capacity that damps both warming and cooling, whereas greater building height (BH) and a higher compactness index (CI) correlate positively, consistent with taller and more compact forms enhancing near-surface ventilation and amplifying short-term heat exchange^16^.

In the frequency model, PM₂.₅ remains the top predictor, contributing 9.99% with a uniformly positive response, indicating that pollution makes it easier to trigger fluctuation days. Ecological factors gain importance. Water density (WD) moves to second at 8.99% and is negatively associated with frequency, consistent with high heat capacity and moist advection suppressing event counts. Vegetation (EVI) shows an inverted-U shape. When cover is sparse, the effect is small, and evapotranspiration only meaningfully reduces the number of days with extreme fluctuations above EVI 0.22. Albedo has its steepest slope below 0.15 and continues to rise thereafter at a slower rate, implying that higher reflectance tends to raise the frequency of fluctuation events. The total contribution of urban morphology increases to 11.63%, with signs consistent with the intensity model. Taller buildings and more compact footprints increase roughness and local turbulence, thereby raising event counts, whereas higher FAR is associated with fewer events. By contrast, the standalone effects of electricity consumption per area (EC_PA) and population per area (POP_PA) decline, although their positive slopes still suggest that anthropogenic heat increases the probability of a fluctuation day. The associations of climatic and geographic background features are shown in Supplementary Fig. 1.

### Aerosol-related importance increases

**Fig. 3:**
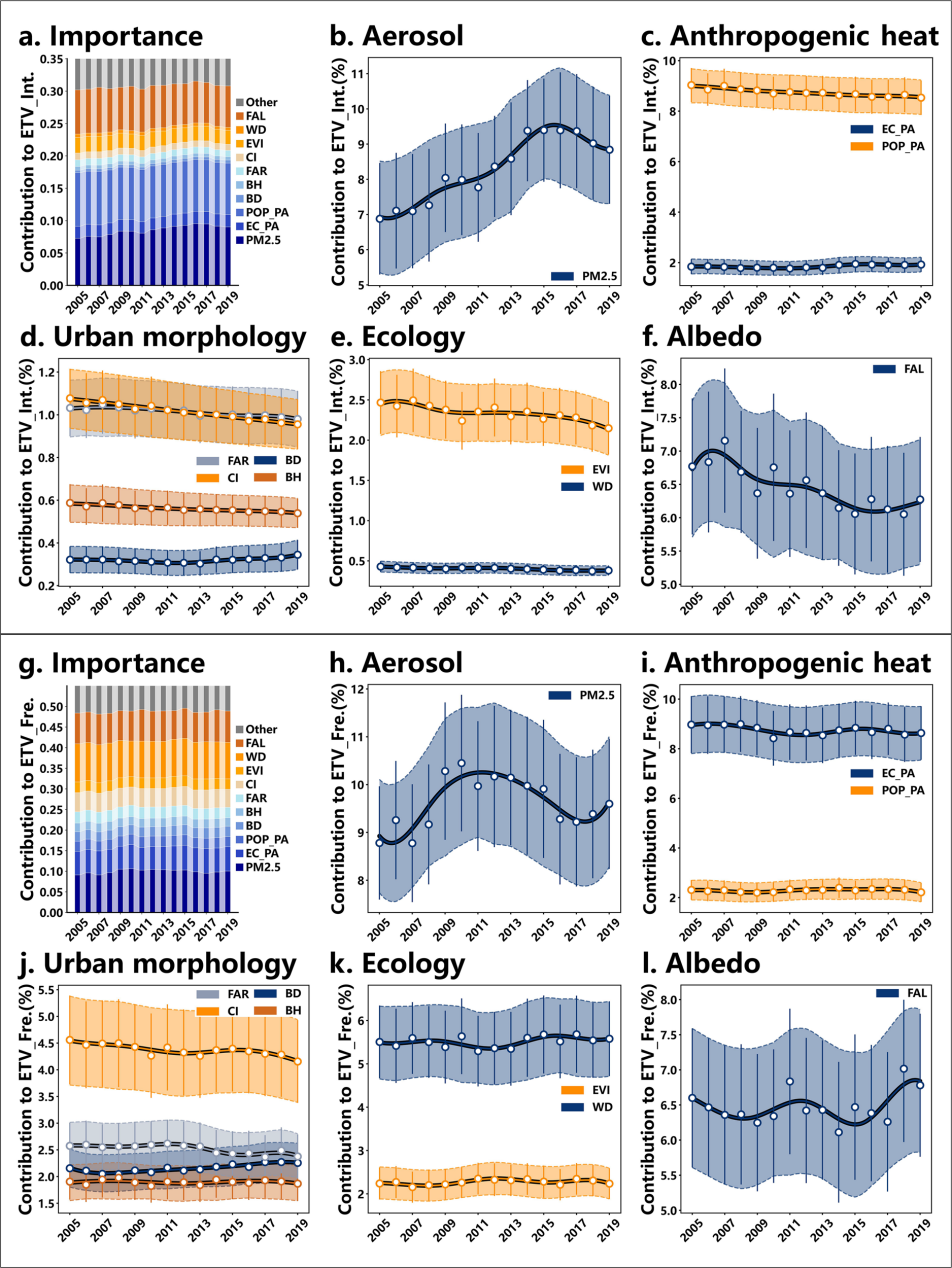
Temporal trends in the impact of urban factors on extreme temperature variability (ETV). **a**, **g** Annual changes in the overall importance of urban features on ETV intensity (ETV_Int) and event frequency (ETV_Fre.) from 2005 to 2019. **b**–**f**, **h**–**l** Trends in the annual marginal contributions for each urban variable. Noted: in these subfigures, the X-axis shows the year, and the Y-axis shows the SHAP value of the sample feature.

From 2005 to 2019, the explanatory power of urbanization processes for global ETV intensity increased by 1.34 percentage points, but the gains were uneven across factors (Fig. 3). PM₂.₅ accounted for almost the entire net rise, with its model weight rising from 7.28% to 9.55%. Over the same period, urban PM₂.₅ levels also increased slightly, making aerosols the most influential urban correlate of ETV amplitude in our models. By contrast, surface albedo dropped from 7.05% to 6.28%, the largest decline among all variables. The aggregate role of urban morphology contracted slightly, yet not uniformly; building density continued to gain weight. Ecological variables (EVI and WD) kept a stable negative association with intensity, but their combined weight slipped by 0.32 points over 15 years. The pattern suggests that expanding blue–green space helps, but it cannot offset the upward pressure on ETV amplitude on its own.

For the frequency dimension of ETV, urban variables still explain about 50%, but their ordering is shifting. Mirroring the intensity results, building density continues to gain weight, while the marginal effects of CI and FAR decline, and the suppressing role of WD weakens. These patterns suggest a combined strategy for urban heat-risk management. Particle abatement caps the amplitude of temperature variability, ventilation in dense districts lowers the probability of threshold exceedance, and blue–green space provides buffering. Together, these measures compress both the size and the count of ETV, shifting cities from the high-frequency, high-amplitude tail of risk into a safer operating space.

### Spatial differences in feature sensitivity on ETV

At the same time, the spatial shifts in variable importance vary sharply across space. Cities in different climate zones, at varying stages of development, and with distinct morphological profiles often exhibit opposite signs and significantly different magnitudes of sensitivity to the same feature. To make heterogeneity in both ETV intensity and frequency visible, we compute city-level local sensitivities for each indicator using a finite-difference perturbation and then assemble global sensitivity maps (see Methods).

**Fig. 4:**
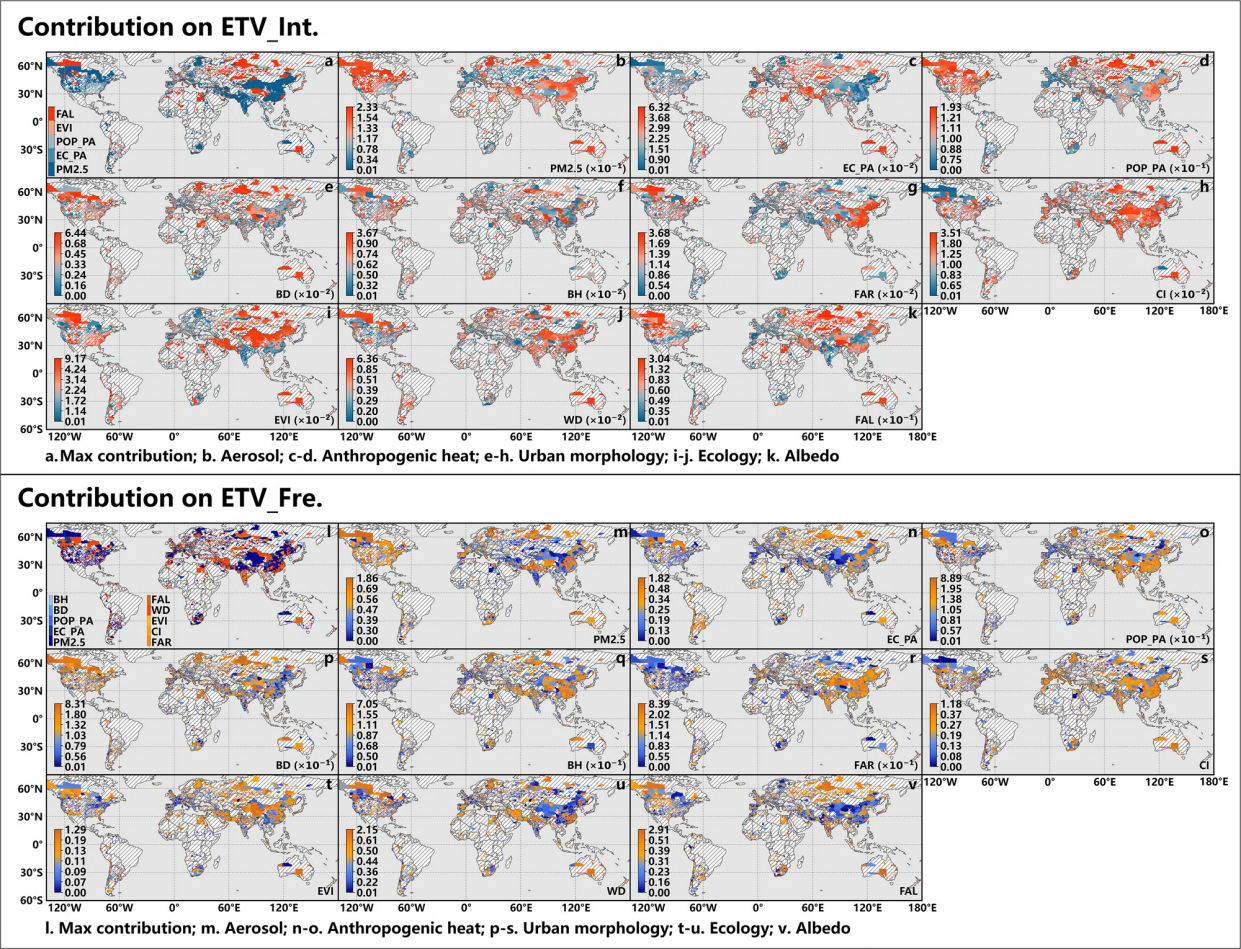
The sensitivity of urban factors on extreme temperature variability (ETV). **a**–**v** Average sensitivities of urban features with respect to ETV, 2005–2019. We assess spatial heterogeneity using partial-derivative analysis; details are provided in Methods.

Global cities exhibit strong spatial heterogeneity in ETV, and the association of intensity and frequency are not fully aligned (Fig. 4). For intensity, low to moderate aerosol levels across North America and Western Europe are dominated by shortwave scattering, which reflects part of the incoming solar radiation and lowers daytime peaks. The Indian subcontinent and East Asia appear to have crossed a concentration threshold. Urban morphology then amplifies this contrast. Dense street canyons suppress ventilation and slow heat removal, keeping high-density districts in South Asia and the Middle East on a trajectory of warming. On the ecological side, greater vegetation and water coverage in Eastern Europe, South Asia, and East Asia reduce intensity through evapotranspiration and the high heat capacity of water.

For ETV frequency, the pattern differs from that of intensity. In North America and Western Europe, PM₂.₅ does not notably reduce the number of threshold-exceedance days. In the Indian subcontinent and East Asia, heavy pollution is associated with a clear increase in frequency, underscoring the urgency of reducing emissions. Energy use, population, and FAR raise the likelihood of day-to-day fluctuations across South Asia, the Middle East, and the industrial belt of the United States. Along the East Asian coast and in European port regions, a compact urban layout can lower the frequency. For ecological variables, vegetation and water suppress frequency mainly where contiguous blue–green bases are well established, such as in the eastern United States and Western Europe.

## Discussion

ETV captures an underexamined dimension of urban climate-risk inequality. Recent work suggests that abrupt day-to-day temperature swings constitute a hazard dimension largely independent of standard ETCCDI temperature-extreme indices across most land areas^4^. Our results extend this perspective to the urban domain by showing that ETV has its own global geography, temporal divergence, and set of urban correlates. Using a 1950–2020 panel of 10,522 cities, we show that city-level ETV follows a coherent global structure shaped jointly by latitude, land–sea thermal contrasts, and development status. High-latitude and inland cities experience the largest weather-scale day-to-day temperature swings, indicating that climatic background sets the baseline geography of variability. Yet these controls do not fully explain recent divergence across cities. Whereas many low-HDI cities show weak declines or relative stability, high-HDI cities continue to intensify, yielding a current gap of about 1.66 °C in ETV intensity.

By decomposing cumulative ETV into event frequency and single-event intensity, we further show that recent divergence across cities is driven primarily by intensity, especially at high latitudes and in high-HDI settings. This matters because a small number of large day-to-day swings can impose disproportionate burdens on health, infrastructure, and energy systems^24–28^. Therefore, the high-HDI may increase intra-urban inequality because vulnerable residents, especially those in dense areas with limited blue–green buffers, face elevated health and energy burdens^29,30^. In that sense, focusing on event amplitude, in addition to event occurrence, provides a more relevant basis for early-warning thresholds, infrastructure design, and preparedness planning.

Aerosols are often treated as a by-product of urban growth^31^. Using data from more than 10,000 cities, we find that aerosols emerge as the leading predictor of ETV in our models. Their relative model importance has increased over the past 15 years and is about four times that of BGI. At low to moderate concentrations, shortwave scattering reduces daytime absorption, speeds the post-event cooldown, and decreases single-event variability in size^32,33^. At higher concentrations, especially where anthropogenic heat is large, enhanced nighttime longwave re-emission raises near-surface thermal inertia^34^. Warm anomalies then decay more slowly, and ETV intensity increases. We identify a global pivot near 20 µg m⁻³. Beyond this level, the damping benefit quickly fades and can even reverse. Even so, these results remain associational rather than causal, due to PM₂.₅ may covary with unobserved factors such as fuel structure, traffic intensity, building operations, or boundary-layer conditions. In addition, urban form and ventilation determine how quickly heat is removed^35^. A higher floor area ratio increases effective heat capacity and dampens both warming and cooling^36^. Taller buildings and more compact layouts, particularly under background winds or frontal passages, can enhance near-surface turbulence and short-term heat exchange^16^. From a policy perspective, this links air-quality management to thermal-volatility management. PM₂.₅ control remains a no-regret option because it yields immediate health benefits and may also help moderate ETV in polluted, energy-intensive settings, especially when paired with albedo management and ventilation-sensitive design.

BGI remains consistently negatively associated with ETV, but its contribution is smaller and more context-dependent than that of its well-established role in reducing mean heat exposure^16,22,26,37^. This suggests that ecological infrastructure can buffer thermal volatility, but its effectiveness depends strongly on surrounding urban form, ventilation, and climatic background. Morphology-related variables also show mixed signs, highlighting trade-offs among heat storage, roughness, shading, and turbulent exchange^16,37^. The policy implication is therefore a place-based portfolio approach rather than a one-size-fits-all intervention logic. High-pollution, high-density cities may benefit most from integrated packages combining emissions reduction, reflective-surface or envelope upgrades, ventilation corridors, and connected blue–green networks. Cities with already strong ecological bases may gain more from protecting continuity, reducing fragmentation, and targeting heat-vulnerable districts. Rapidly urbanizing cities may reduce future ETV lock-in by embedding ecological continuity, street ventilation, and surface-property management early in land-use planning^38,39^.

More broadly, the value of this analysis lies less in prescribing a universal intervention package than in informing policy sequencing and regional prioritization. In South Asia and East Asia, where heavy pollution is associated with a greater risk of fluctuations, air-quality control should be paired with albedo management and ventilation-sensitive design. In dense urban corridors of the Middle East and the United States, morphology-sensitive retrofits and ventilation corridors may be more effective than green expansion alone. By contrast, cities in Western Europe, Eastern Europe, and the eastern United States may gain more from protecting and connecting existing blue–green networks. Because the present study is observational and retrospective, these directions should be interpreted as management-relevant rather than as causal or scenario-tested prescriptions.

Finally, we acknowledge several limitations and outline the next steps. Our analysis is designed for the city scale. First, although the combined LightGBM–GAM framework improves the detection and interpretation of nonlinear associations, it does not fully overcome the limitations of data-driven modeling. The estimated relationships may also be affected by omitted variables, residual confounding, and limited extrapolation beyond the observed climatic and urban conditions. Future work should couple high-resolution atmospheric models with urban canopy, building-energy, and aerosol–radiation–boundary-layer schemes to explicitly resolve radiation, heat storage, anthropogenic heat, turbulent exchange, and hydrological buffering. Controlled factorial simulations under matched synoptic conditions, validated against station, flux-tower, and remote-sensing observations, would help determine whether the associations identified here reflect causal physical pathways. Hybrid physics-informed machine-learning approaches may further combine global scalability with stronger process constraints. Second, because ETV is derived from gridded daily near-surface air temperature, the dataset cannot fully resolve neighborhood- or canopy-scale thermal heterogeneity. Our conclusions should therefore be interpreted as city-level patterns rather than direct evidence of street-scale urban heat processes. Finally, future research should include future projections from CMIP6 models to assess trends in ETV across different scenarios. Despite these limitations, our harmonized analysis of 10,522 cities shows that urbanization is reshaping the intensity and frequency structure of temperature risk. By disentangling how urbanization processes interact with the climatic background, we outline a practical path to steer cities out of the high-frequency, large-amplitude tail of risk and back into a safer operating space.

## Methods

### Extreme temperature variability

In this study, the city is treated as the spatial unit of analysis with second-level administrative units from the GADM global database, and annual indicators from 1950 to 2020 are constructed to characterize city-level ETV. To quantify day-to-day temperature variability, we calculated daily temperature differences and smoothed these differences using a five-day rolling window. This window was chosen to represent weather-scale thermal fluctuations associated with synoptic variability while reducing sensitivity to isolated single-day noise. To assess the robustness of this choice, we additionally repeated the analysis using 3-day and 7-day rolling windows, and the main results remained qualitatively consistent across specifications. The complete calculation is given as follows:

1 $${\Delta T}_{c,d,y,W}=\frac{{\sum }_{x}^{{N}_{x}}{\sum }_{w=1}^{W}\left|{T}_{x,d,y}-{T}_{x,d+1,y}\right|}{W\cdot {N}_{x}}$$

The variable ${\Delta T}_{c,d,{y},W}$ quantifies the day-to-day temperature variability in a given city *c*, for a specific calendar day *d* and year *y*. This metric is derived from daily mean temperatures ${T}_{x,d,y}$ for each grid cell *x* within the city’s boundaries, obtained from the ERA5-Land dataset, covering historical daily 2-meter temperatures from 1950 to 2020. Here, *W* denotes the rolling window length (3, 5, and 7 days), and ${N}_{x}$ represents the total number of grid cells within the city.

We developed a consistent metric for monitoring and assessing ETV by integrating the TX90 extreme index, recommended by the Expert Team on Climate Change Detection and Indices (ETCCDI: https://www.climdex.org/learn/indices/), with the day-to-day temperature variability index ${\Delta T}_{c,d,{y},W}$. For clarity, we distinguish three related but conceptually different annual metrics derived from daily ETV events. We first calculate daily weather-scale temperature variability and define an ETV event day as a day when this daily value exceeds a calendar-day-specific 90th-percentile threshold. Based on these event days, we derive annual cumulative ETV, defined as the total exceedance load accumulated over a year; event frequency, defined as the number of event days per year; and event intensity, defined as the mean exceedance magnitude of event days. Annual cumulative ETV, therefore, reflects the combined outcome of how often ETV events occur and how large they are, whereas event frequency and single-event intensity isolate these two components. The complete calculation formula is defined as follows:

2 $${{ETV}}_{c,y} 	={\sum }_{d =1}^{{N}_{y}}{{ET}D}_{c,d,y,W},\,{{ETD}}_{c,d,y,W} \\ 	=\left\{\begin{array}{c}{\Delta T}_{c,d,y,W}-{\Delta T90}_{c,d,y,W},\,{\Delta T}_{c,d,y,W} > {\Delta T90}_{c,d,y,W}\\ 0,\,\,\,\,\,\,{\Delta T}_{c,d,y,W}\le {\Delta T90}_{c,d,y,W}\end{array}\right.$$

3 $${{ETV}\_{Fre}}_{c,y}=\frac{{\sum }_{d=1}^{{N}_{y}}{{EDay}}_{c,d,y,W}}{{N}_{y}},\,{{EDay}}_{c,d,y,W} \\=\left\{\begin{array}{c}1,\,\,\,\,\,\,\,{\Delta T}_{c,d,y,W} > {\Delta T90}_{c,d,y,W}\\ 0,\,\,\,\,\,\,\,{\Delta T}_{c,d,y,W}\le {\Delta T90}_{c,d,y,W}\end{array}\right.$$

4 $${{ETV}\_{Int}}_{c,y} 	=\frac{{\sum }_{d=1}^{{N}_{y}}{{ET}}_{c,d,y,W}}{{{ETV}}_{{Fre}}\cdot {N}_{y}},\,{{ET}}_{c,d,y,W} \\ 	=\left\{\begin{array}{c}{\Delta T}_{c,d,y,W},\,{\Delta T}_{c,d,y,W} > {\Delta T90}_{c,d,y,W}\\ 0,\,\,\,\,\,\,\,{\Delta T}_{c,d,y,W}\le {\Delta T90}_{c,d,y,W}\end{array}\right.$$

${{ETV}}_{c,y}$ measures the annual ETV for a given city *c* in year *y*. Specifically, ${\Delta T90}_{c,d,y,{W}}$ denotes the 90th percentile threshold computed from a seven-day rolling window centered on each calendar day *d* during the baseline period (1950–1990). For daily temperature differences exceeding this threshold, the excess difference is recorded as ${{ETD}}_{c,d,y,{W}}$ and ${N}_{y}$ is the total number of days in year *y*. The frequency-based metric, ${{ETV\_Fre}}_{c,y}$, quantifies the occurrence of ETV events within city *c* in year *y*. For each calendar day *d*, an event day indicator ${{EDay}}_{c,d,y,{W}}$ is assigned a value of 1 if ${\Delta T}_{c,d,{y},W}$ exceeds the threshold, and 0 otherwise. The intensity-based metric, ${{ETV\_Int}}_{c,y}$, measures the magnitude of ETV events within city *c* in year *y*. For each calendar day *d*, the daily intensity ${ET}_{c,d,y,{W}}$ is recorded as the value of ${\Delta T}_{c,d,{y},W}$ if the threshold is exceeded, and 0 otherwise.

### Urbanization-related Indicators

To systematically characterize urbanization in relation to ETV, the selection of urban indicators was guided by an urban surface energy-balance framework, in which cities influence the thermal environment through five major pathways: radiative forcing, anthropogenic heat input, aerodynamic and heat-storage effects of urban form, hydrological buffering by vegetation and water, and surface reflectance^14^. Therefore, we compiled five categories of raster-based indicators with global coverage and annual observations for 2005–2019, the period for which urban covariates are consistently available. For each year, these variables were aggregated to city-level means within each city’s contemporaneous built-up footprint and then used in subsequent urban-factor analyses. City boundaries were derived from annually updated built-up extents based on multi-source remote-sensing products^40^. Because our analysis targets city-level, weather-scale ETV rather than neighborhood-scale thermal heterogeneity, and to reduce the mismatch between dynamic urban polygons and the underlying meteorological grid, we retained only cities with a built-up area of at least 10 km² during the urban-factor analysis period. This filter excludes very small settlements and improves the global comparability of both urban extent and urban descriptors. A total of 10,522 cities met these criteria and were included in the analysis. Note that ETV itself was calculated for 1950–2020, whereas analyses linking ETV to urbanization-related factors were restricted to 2005–2019 owing to data availability.

Aerosols modulate surface net radiation via dual-directional effects of shortwave scattering and longwave warming, constituting a critical pathway through which urbanization influences ETV. We employed the SatPM2.5 global ground-level PM₂.₅ dataset developed by the Atmospheric Composition Analysis Group as a proxy for aerosols. The dataset integrates multi-source satellite observations with the GEOS-Chem chemical transport model, calibrated using ground monitoring stations worldwide via a residual convolutional neural network, providing a globally consistent PM2.5 raster dataset at a spatial resolution of 1 km^41^.

Anthropogenic heat emissions constitute explicit urban heat sources, primarily arising from sensible heat fluxes associated with human metabolism, daily activities, and energy production. We characterized anthropogenic heat using two complementary metrics: population density per unit area (POP_PA) and electricity consumption per unit area (EC_PA). Population data were derived from the WorldPop global population raster dataset, calibrated using official statistics from the United Nations Population Division, with a spatial resolution of 1 km at the equator^42^. Electricity consumption data were obtained from a global annual 1 km × 1 km gridded dataset for 1992–2019, based on calibrated nighttime light observations and a top-down estimation framework linked to national electricity statistics^43^. In this study, EC_PA was used as a city-level proxy for anthropogenic heat intensity. Because this dataset is model-derived, it is suitable for representing broad spatial differences in electricity use, but it may not fully capture sector-specific demand structure, end-use heterogeneity, or short-term local variation.

Urban morphology indicators directly determine urban surface thermodynamic characteristics, thereby influencing ETV. We utilized the Global Human Settlement Layer (GHSL) dataset (https://ghsl.jrc.ec.europa.eu/) to compute building density (BD), building height (BH), and floor area ratio (FAR). GHSL provides global built-up area distribution and three-dimensional morphological features at a high spatial resolution of 100 meters. Given the dataset’s five-year update cycle, we employed linear interpolation to fill annual-scale data gaps, ensuring temporal continuity. Furthermore, we introduced the compactness index (CI) to objectively quantify the spatial morphology of urban areas. The CI is calculated as follows:

5 $${{CI}}_{c,y}=\frac{{A}_{c,y}}{{CA}_{c,y}}$$

${A}_{c,y}$ denotes the built-up area for a given city *c* in year *y*, ${CA}_{c,y}$ represents the area of the smallest circle surrounding city *c* in year *y*. The closer the ${{CI}}_{c,y}$ value is to 1, the more compact and orderly the city is.

Regarding urban ecological factors, we used the Enhanced Vegetation Index (EVI) derived from the MODIS vegetation index product MOD13A1, which has a stable monthly update frequency and a spatial resolution of 1 km. Monthly EVI values were aggregated annually to compute the mean annual EVI within each city boundary, providing a comprehensive representation of urban vegetation levels throughout the study period. For blue infrastructure, we utilized the Global WaterPack dataset provided by the German Aerospace Center. This dataset tracks the global distribution and daily dynamics of water bodies with a high spatial resolution of 250 meters^44^. Based on this data, we calculated the annual average water density (WD) within each city boundary to assess the dynamic features of urban blue infrastructure.

For urban albedo, a critical attribute directly determining the absorption and reflection of incident shortwave radiation by urban surfaces, which can be rapidly modified through roofing, paving materials, and surface coatings, we adopted the forecast albedo (FAL) dataset from ERA5-Land. This dataset provides a daily time series of surface albedo with a spatial resolution of 10 km. We aggregated daily FAL values both annually and spatially to derive city-level annual mean albedo metrics, effectively capturing urban surface responses to incoming shortwave radiation.

### Climatic and geographical background

This study obtained total precipitation (TP), wind speed (WS), and downward surface shortwave radiation (DSSR) from the ERA5-Land dataset. These climate variables, available as daily time series at 0.1° spatial resolution, were aggregated to the city scale by computing mean values across each city’s grid cells. Additionally, elevation data were derived from the Shuttle Radar Topography Mission (SRTM) Digital Elevation Model (DEM) at 15-arc-seconds resolution^45^. Ocean distance (Ocean_D) was calculated as the distance from each city’s center to the nearest coastline.

### Empirical modeling approach

To effectively reveal the complex nonlinear interactions between urban characteristics and ETV, this study combines gradient boosting decision trees (LightGBM) and the Generalized Additive Model (GAM) to address our core research question^46–49^. LightGBM is used to capture nonlinear effects and high-order interactions, quantify feature importance, and visualize local response patterns, whereas GAM serves as a complementary semi-parametric framework to assess the statistical significance, functional direction, and robustness of the identified associations.

Two separate sets of models are constructed using ETV_Int. and ETV_Fre. as dependent variables, thereby capturing different dimensions of urban influence on ETV. Influencing factors are divided into two distinct categories: explanatory urban feature variables and background control variables. Urban feature variables, including PM₂.₅, POP_PA, EC_PA, BD, BH, FAR, CI, EVI, WD, and FAL, are introduced to characterize direct impacts on ETV arising from human activities, spatial morphology, and ecological infrastructure. Background control covariates, including Lat, Ocean_D, DEM, WS, TP, and DSSR, were incorporated to account for broad-scale geographic and climatic conditions and thereby reduce confounding.

During model training and optimization, we apply Bayesian optimization (BO), leveraging probabilistic surrogate models to approximate the complex relationship between LightGBM hyperparameters and model performance^50^. This approach enables rapid and efficient hyperparameter tuning, significantly reducing computational load, ensuring efficient convergence to optimal solutions within a complex parameter space, and enhancing model prediction performance and stability.

To enhance the robustness of the machine learning results and address inherent challenges related to interpretability, this study integrates the Shapley Additive Explanations (SHAP) algorithm and Monte Carlo Simulation (MCS) to systematically reveal feature importance and their nonlinear effects on ETV. SHAP was used to generate local explanations for the fitted LightGBM models by quantifying each feature’s contribution to individual predictions, thereby enabling both global importance ranking and visualization of local nonlinear associations with ETV^51^. Because SHAP values are conditional on the fitted predictive model, they are interpreted here as model-based associations rather than causal effects. To assess the stability of these interpretations, we conducted 500 independent MCS runs to capture uncertainty arising from repeated model fitting. Subsequently, the SHAP values obtained from all simulations are aggregated to construct robust distributions of feature importance, thereby reinforcing the reliability of model interpretation^52^. Finally, to quantitatively characterize the nonlinear relationships between each feature and ETV, we employ restricted cubic spline (RCS) fitting to the aggregated SHAP-based partial dependence plots, thereby clearly delineating the marginal association patterns and trend dynamics of different variables with respect to ETV.

As a complementary validation, we further fit GAMs for ETV_Int. and ETV_Fre. to evaluate whether the nonlinear associations identified by LightGBM-SHAP remain statistically significant under a more transparent semi-parametric specification.

6 $${ETV}={\beta }_{0}+{\sum }_{j=1}^{p}{{f}_{j}}(x_{{ij}})+\varepsilon$$

where ${x}_{{ij}}$ denotes the set of urban feature variables and background control variables. Because the relationships between explanatory variables and ETV may be strongly nonlinear, all continuous predictors enter the model as smooth functions ${f}_{j}(\cdot )$. As both ETV_Int. and ETV_Fre. are treated as annual city-level continuous outcomes, the GAMs are fitted using a Gaussian family with an identity link. To reduce the influence of extreme values and improve model stability, the strongly right-skewed variables POP_PA and EC_PA are transformed using log1p prior to model fitting. The fitted smooth terms are used to evaluate the direction, shape, and statistical significance of each association, and the estimated response curves are compared with the LightGBM-SHAP results to assess cross-model consistency.

### Sensitivity analysis

To systematically explore the sensitivity of model outputs to individual input features, we conducted a local sensitivity analysis to quantify the localized influence of each feature on the predictions^53^. Specifically, numerical partial derivatives of the model outputs with respect to each input feature were approximated using the finite-difference method, enabling the assessment of how variations in each feature affect predicted outcomes under given input conditions. To ensure that these numerical derivatives accurately reflect realistic variations in the features, we adopted the standard deviation of each feature as the perturbation magnitude. The rationale for this selection is that the standard deviation naturally captures the inherent variability within each feature’s observed data range, thereby avoiding bias introduced by arbitrarily fixed perturbation values and capturing sensitivity in a realistic context. The numerical partial derivative is calculated as follows:

7 $$\frac{\partial {y}_{i}^{p}}{\partial {X}_{i}^{p}}\approx \frac{f\left({X}_{i}^{p}+{\sigma }_{p}/100\right)-f({X}_{i}^{p}-{\sigma }_{p}/100)}{{\sigma }_{p}/50}$$

In the calculation, we perturb each feature slightly within its natural range of variation to get the amount of variation in the model output $\partial {y}\,_{i}^{p}$ to estimate the local sensitivity of the *p* feature. f(⋅) denotes the fitted SHAP-RCS model, ${X}_{i}^{p}$ is the value of *i* point in the *p* feature, and ${\sigma }_{p}$ represents the standard deviation of the *p* feature.

### Spatial Cross-Correlation of Exposure and Equity

Global Moran’s I was employed to assess spatial autocorrelation^54^, calculated as:

8 $$I=\frac{n}{{\sum }_{i=1}^{n}{\sum }_{j=1}^{n}{\omega }_{{ij}}}\times \frac{{\sum }_{i=1}^{n}{\sum }_{j=1}^{n}{\omega }_{{ij}}({x}_{i}-\bar{x})({x}_{j}-\bar{x})}{{\sum }_{i=1}^{n}{({x}_{i}-\bar{x})}^{2}}$$

In the calculation, ${x}_{i}$ and ${x}_{j}$ denote the values of the variable at cities i and j, respectively, and $\bar{x}$ represents the mean value across all cities. The elements ${\omega }_{{ij}}$ form the spatial weight matrix W, which encodes the spatial adjacency between city i and its neighboring cities j. To represent spatial adjacency, we constructed W using a *k*-nearest-neighbor scheme, so that each spatial unit is connected to its *k* geographically closest neighbors. In this study, we set k=100, meaning that each spatial cell is treated as adjacent to its 100 nearest cells, thereby capturing broad-scale spatial dependence in the gridded field.

To assess local spatial cross-correlation between two spatial variables, we further employed the Bivariate Local Moran’s I statistic to identify localized spatial associations across geographic units in the study area^55^. The basic formula is:

9 $${I}_{i}^{{pq}}={Z}_{i}^{p}{\sum }_{j=1}^{n}{\omega }_{{ij}}{Z}_{j}^{q}$$

where ${{{{\rm{Z}}}}}_{{{{\rm{i}}}}}^{{{{\rm{p}}}}}$ and ${{{{\rm{Z}}}}}_{{{{\rm{j}}}}}^{{{{\rm{q}}}}}$ are the standardized (zero-mean, unit-variance) values of variables p and q at locations i and j, respectively, and ${\omega }_{{ij}}$ are the elements of the spatial weight matrix defined above. A positive value of the local bivariate Moran’s I indicates that a high (or low) value of variable p at location i is surrounded by similarly high (or low) values of variable q in neighboring locations, forming high–high or low–low clusters. Conversely, a negative value implies spatial dissimilarity, where high values of p tend to be adjacent to low values of q (or vice versa), producing mixed high–low or low–high patterns.

## Supplementary information

Supplementary Information

Transparent Peer Review file

## Data Availability

Data used in this study are available on Zenodo at 10.5281/zenodo.20275372.

## Code availability

Code for the main modeling and analysis process is available on Zenodo at 10.5281/zenodo.20275372.
