# Supplementary material for: Urbanization processes widen global divergence in extreme temperature variability
Source: Nat Commun. 2026 Jul 8;17:8416. doi: 10.1038/s41467-026-75457-z (PMC13478471; doi:10.1038/s41467-026-75457-z)
Supplement: Supplementary file 1 [file 41467_2026_75457_MOESM1_ESM.pdf]

Urbanization processes widen global divergence in extreme temperature variability

SUPPLEMENTARY INFORMATION

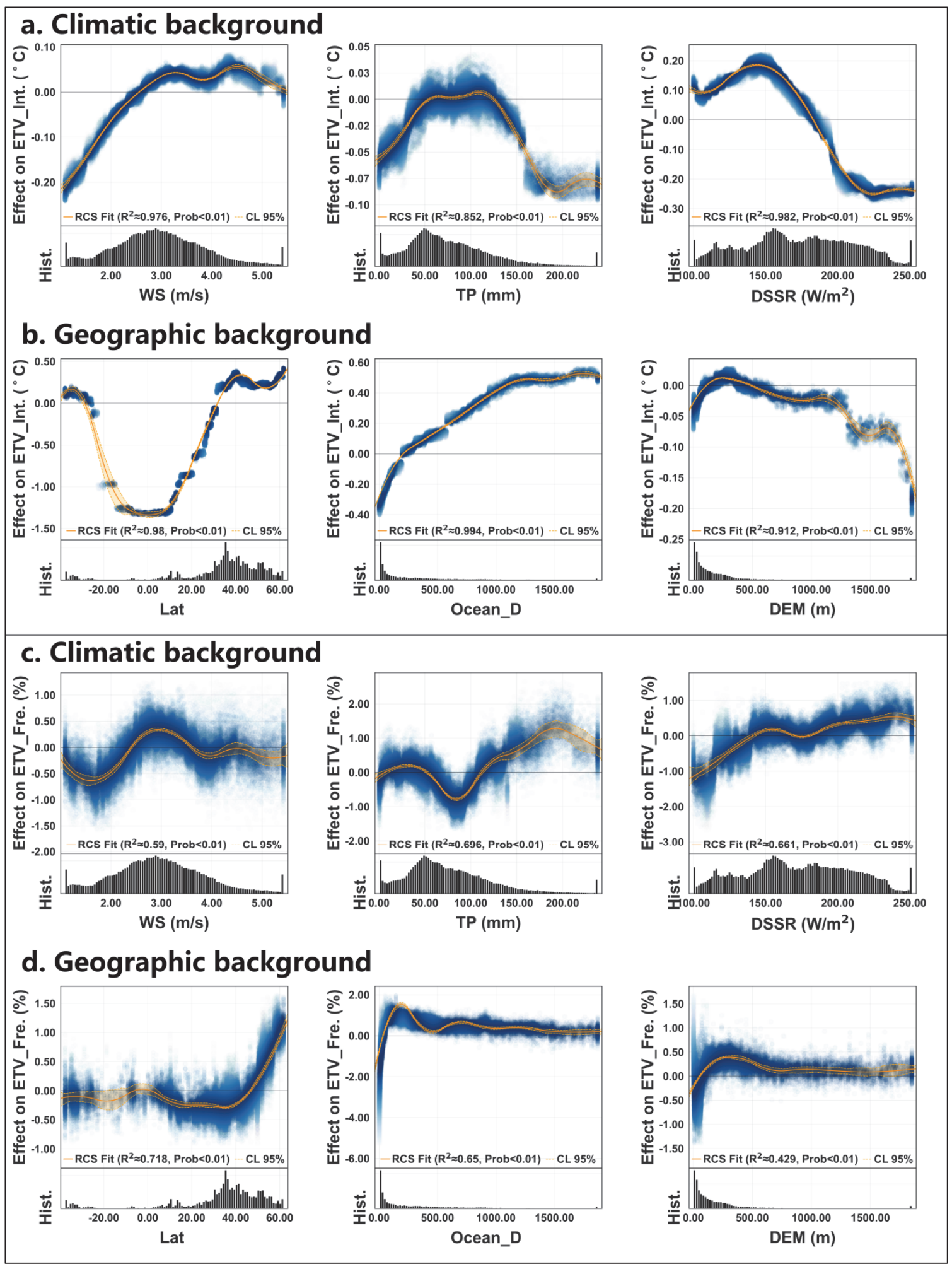

Supplementary Fig. 1 The effect of climatic and geographic background features on ETV. Local effect of each feature included in ensemble learning models on ETV_Int. (a and b) and ETV_Fre. (c and d) (n=157,830).

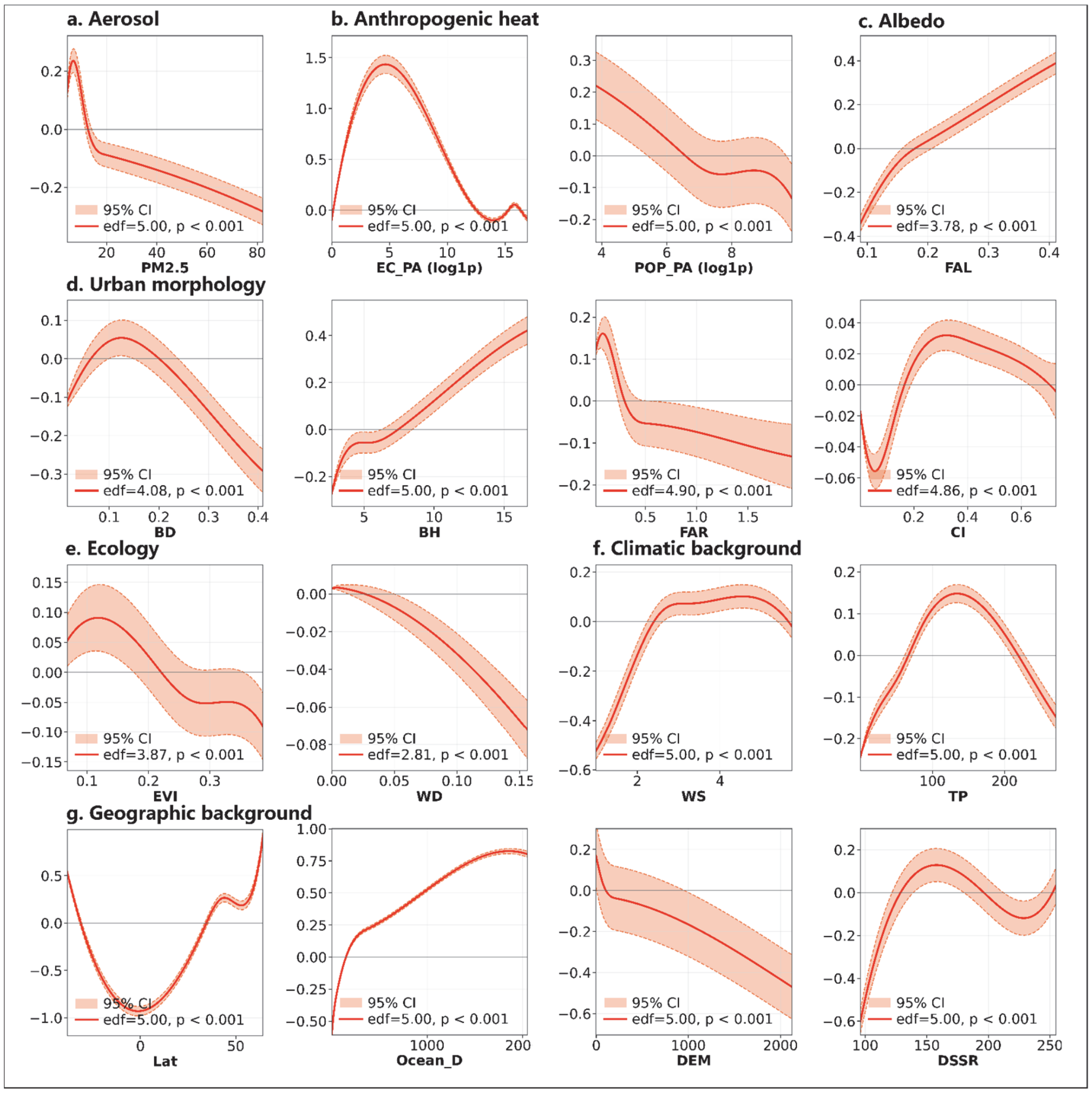

Supplementary Fig. 2 The GAM results on ETV_Int. Smooth effect curve and statistically significant level of each feature included in GAM on ETV_Int.(n=157,830).

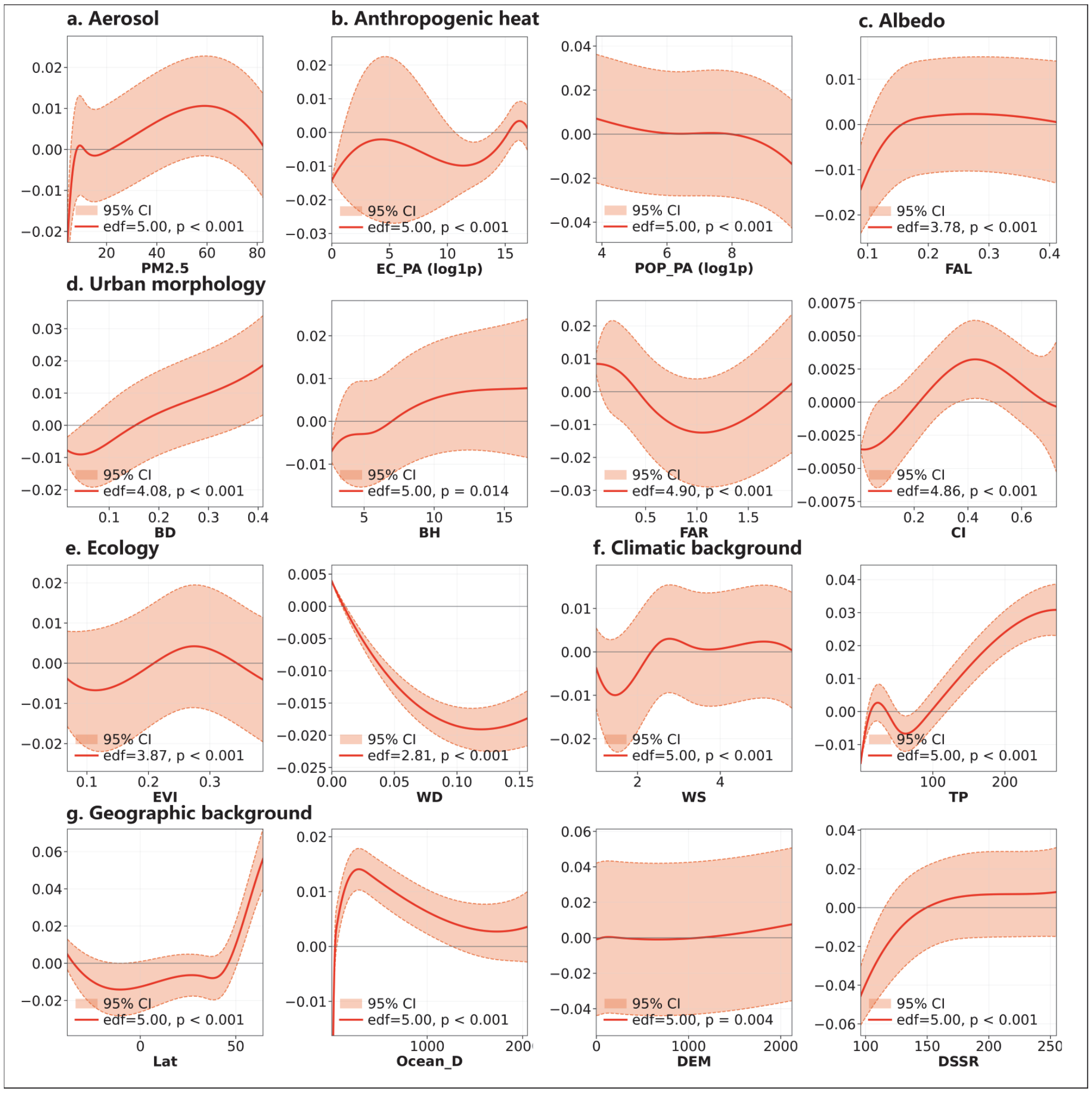

Supplementary Fig. 3 The GAM results on ETV_Fre. Smooth effect curve and statistically significant level of each feature included in GAM on ETV_Fre.(n=157,830).

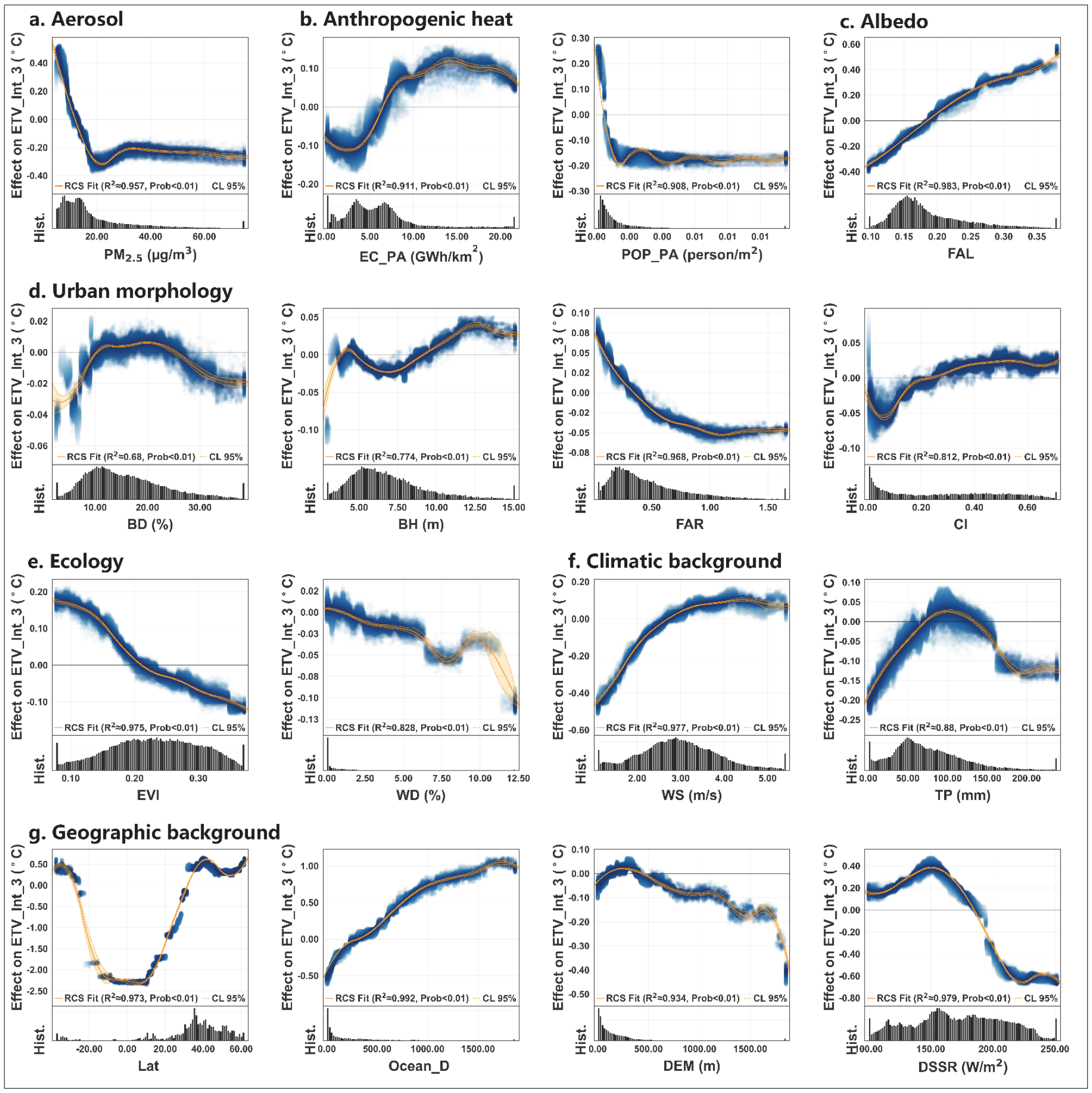

Supplementary Fig. 4 The results on ETV_Int. using a three-day rolling window. Local effect of each feature included in ensemble learning models on ETV_Int. using a three-day rolling window (n=157,830).

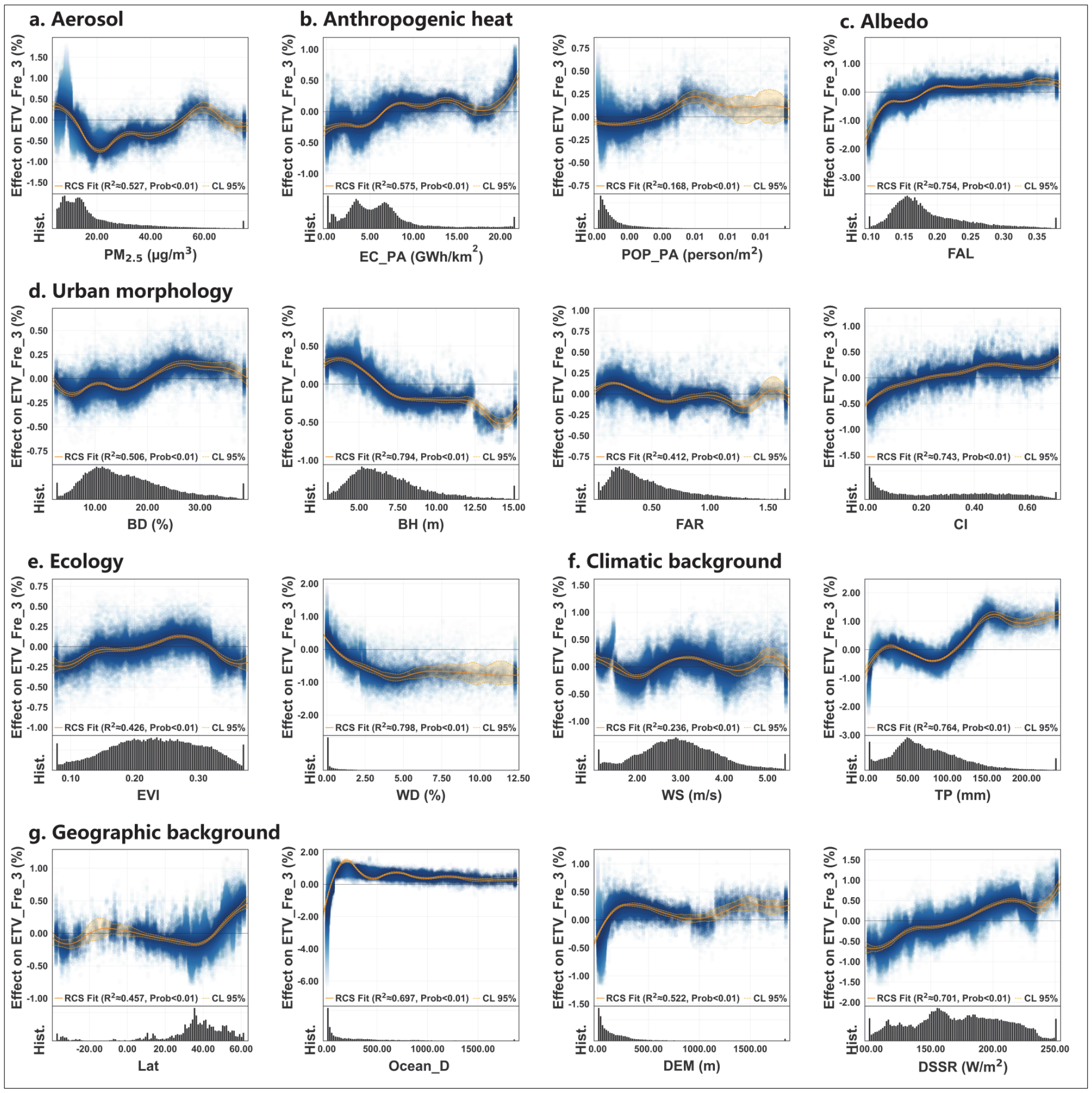

Supplementary Fig. 5 The results on ETV_Fre. using a three-day rolling window. Local effect of each feature included in ensemble learning models on ETV_Fre. using a three-day rolling window (n=157,830).

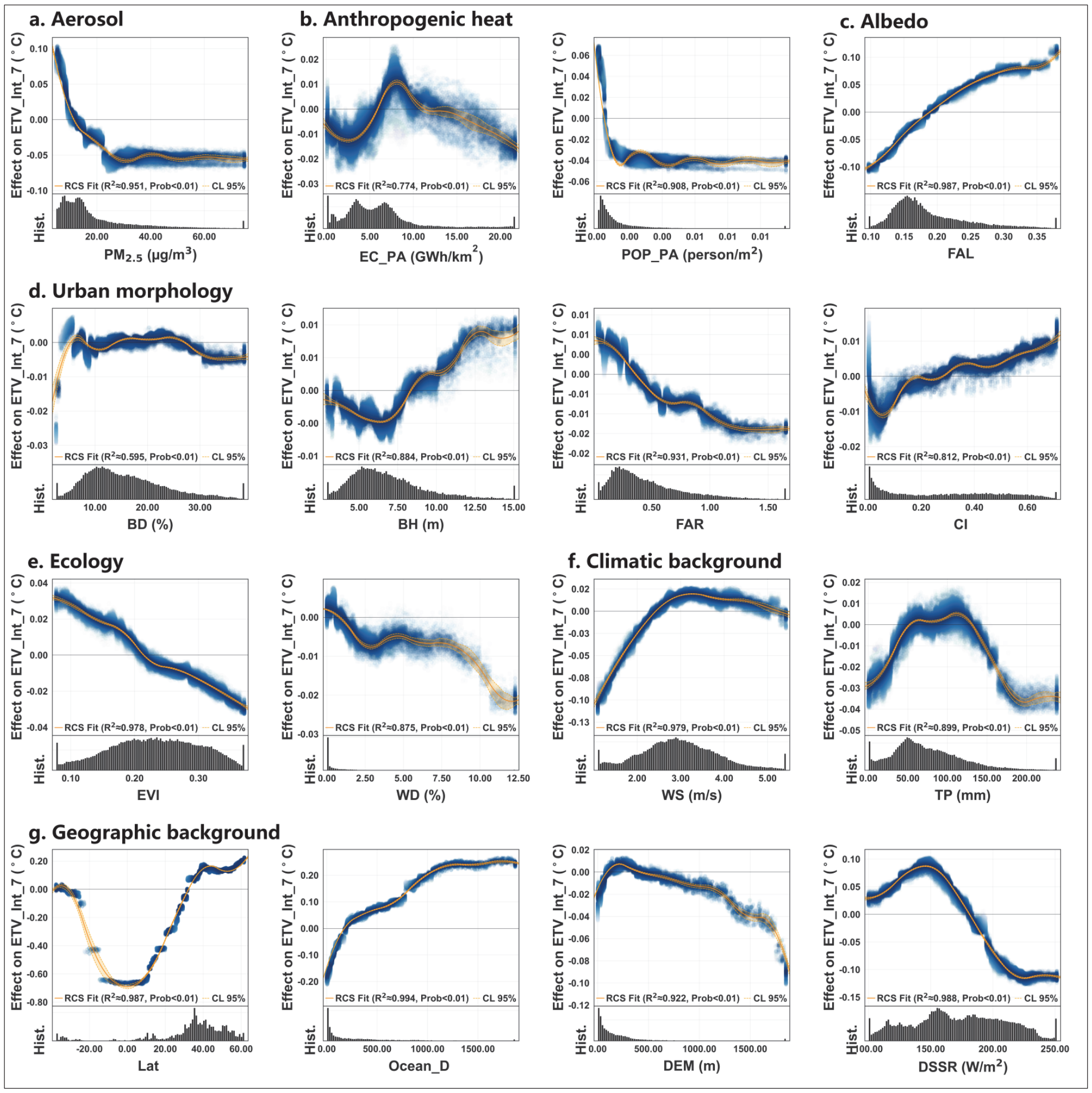

Supplementary Fig. 6 The results on ETV_Int. using a seven-day rolling window. Local effect of each feature included in ensemble learning models on ETV_Int. using a seven-day rolling window (n=157,830).

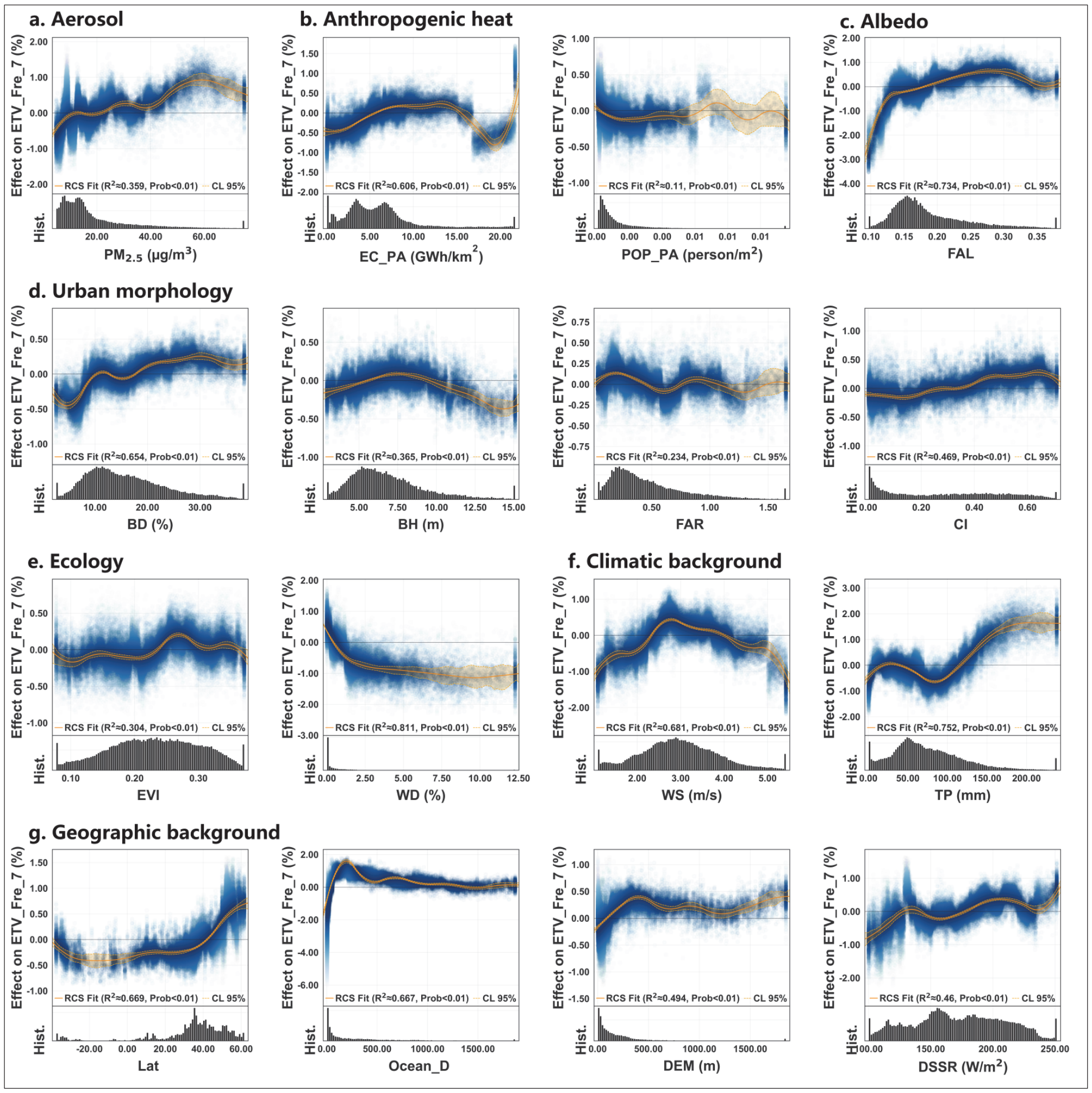

Supplementary Fig. 7 The results on ETV_Fre. using a seven-day rolling window. Local effect of each feature included in ensemble learning models on ETV_Fre. using a seven-day rolling window (n=157,830).

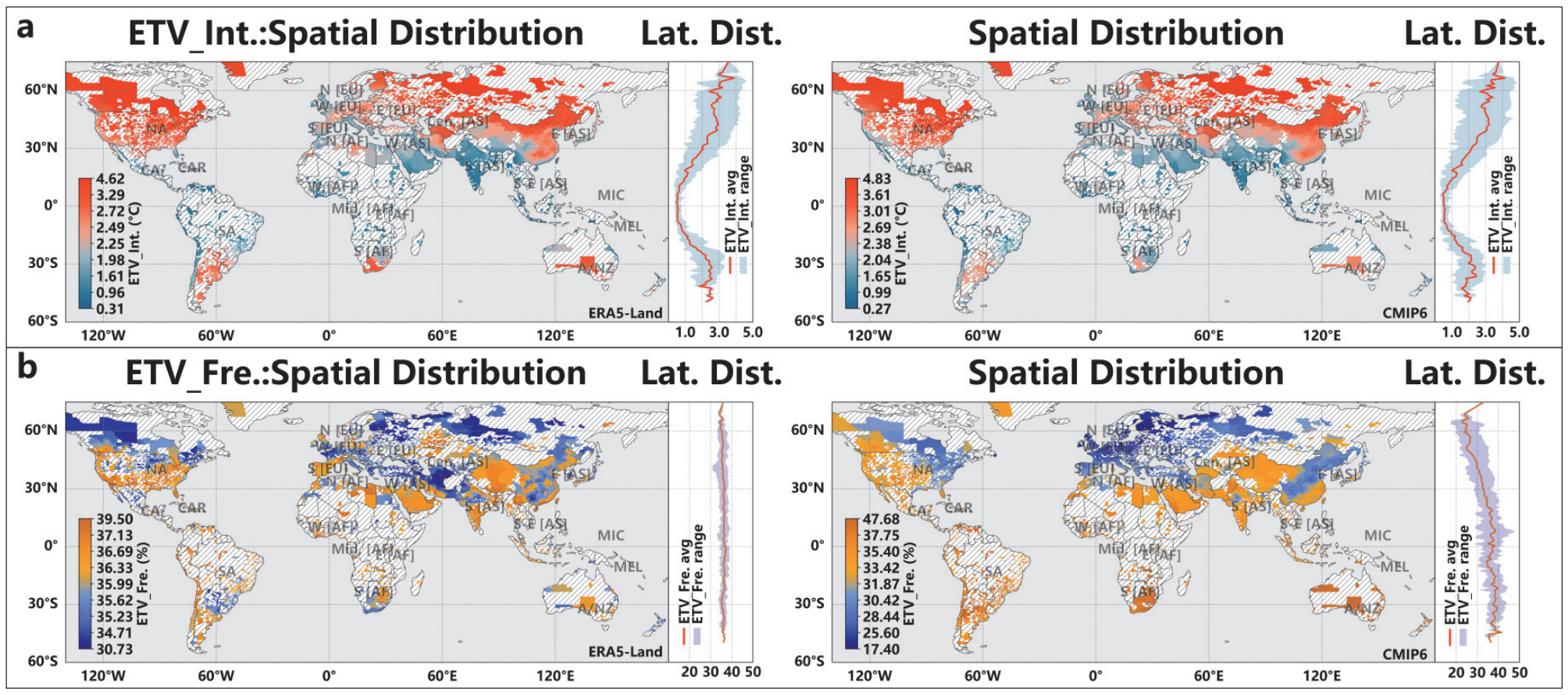

Supplementary Fig. 8 Spatio-temporal distribution of ETV between ERA5-Land and CMIP6. We used daily near-surface air temperature from the EC-Earth3 climate model, which participated in CMIP6, from 2005 to 2019.

Supplementary Table. 1

**Category**	**Variable**	**Unit**	**Theoretical motivation**
Aerosol	$PM_{2.5}$	μg/m^3	Modifies radiation balance and boundary-layer stability, thereby shaping thermal variability.
Anthropogenic heat	EC_PA	GWh/km^2	Proxies energy use and associated sensible heat release within cities.
	POP_PA	person/m^2	Captures human activity intensity and its associated heat and emission load.
Urban morphology	BD	%	Alters impervious cover, shading, ventilation resistance, and heat storage.
	BH	m	Regulates roughness, canyon trapping, and turbulent heat exchange.
	FAR	dimensionless	Reflects built volume intensity and the effective thermal capacity of urban form.
	CI	dimensionless	Captures urban compactness, with implications for ventilation and heat dispersion.
Ecology	EVI	dimensionless	Represents vegetation cover and its evaporative and shading effects.
	WD	%	Represents water coverage and its thermal buffering through high heat

			capacity.
Albedo	FAL	dimensionless	Controls shortwave reflection and surface heat absorption.
Climatic background	WS	m/s	Governs ventilation strength and near-surface heat transport.
	TP	mm	Reflects moisture conditions that modify evaporative cooling and thermal response.
	DSSR	W/m^2	Represents incoming solar energy available for daytime surface heating.
Geographic background	Lat	dimensionless	Captures large-scale gradients in seasonality, radiation, and circulation.
	Ocean_D	km	Represents the strength of marine moderation on day-to-day temperature swings.
	DEM	m	Captures topographic control on local thermal conditions and airflow.
