# Supplementary material for: Urbanization processes widen global divergence in extreme temperature variability
Source: Nat Commun. 2026 Jul 8;17:8416. doi: 10.1038/s41467-026-75457-z (PMC13478471; doi:10.1038/s41467-026-75457-z)
Supplement: Supplementary file 2 [file 41467_2026_75457_MOESM2_ESM.pdf]

nature portfolio

Peer Review File

Urbanization processes widen global divergence in extreme temperature variability

Corresponding Author: Dr Renlu Qiao

This file contains all reviewer reports in order by version, followed by all author rebuttals in order by version.

Version 0:

Reviewer comments:

Reviewer #1

(Remarks to the Author)
This manuscript investigates extreme temperature variability (ETV) across over 10,000 cities worldwide over the past seven decades and applies interpretable machine-learning methods to identify potential drivers. The topic is timely and relevant to urban climate and heat mitigation research. However, despite the impressive global scope of the dataset, I find that the study falls short of the standards for Nature Communications and would be better suited for a specialized journal in ecology and environmental sciences. My specific concerns are outlined below:
1.The analysis encompasses more than 10,000 cities, many of which are spatially small—often only a few square kilometers. The study primarily uses reanalysis data to characterize urban-scale ETV and to infer urbanization effects. Given the coarse spatial resolution of reanalysis products, it is unlikely they can adequately resolve genuine urban thermal signals at such fine scales, casting doubt on the robustness of the inferred urbanization effects and overall validity of the results.
2.The finding that ETV is higher in mid- to high-latitude regions and continental interiors compared to low-latitude and coastal areas is largely expected, as it reflects background climatic variability. Attributing these large-scale patterns primarily to urban development introduces a conceptual gap. Moreover, ETV involves complex physical mechanisms related to surface energy balance, atmospheric circulation, and radiative processes. The manuscript relies almost exclusively on explanatory machine-learning models without adequate theoretical grounding or mechanistic validation, leaving the conclusions largely correlational and vulnerable to “black-box” interpretation.
3.The discussion and policy implications remain underdeveloped. The core conclusions—that aerosols and blue-green infrastructure (BGI) influence temperature variability—are already well-established in urban climate literature. Thus, the study does not offer substantial conceptual advancement or a new theoretical framework; rather, it provides a large-scale quantitative synthesis of previously recognized relationships. Although the authors briefly suggest interventions such as aerosol reduction, expanded BGI, and improved urban form, these recommendations lack empirical validation or scenario-based assessment, limiting the study’s utility for actionable urban climate governance.
4.The heatmaps in Fig. 1a and 1b, intended to illustrate interannual ETV variability across regions, suffer from poor visual clarity. The regional subdivision is overly granular, making it difficult to discern coherent patterns. A coarser regional classification or alternative visualization approach would improve interpretability.
5.In Fig. 4, the individual global maps are presented at too small a scale to allow meaningful interpretation. The multi-panel layout hinders the reader’s ability to identify spatial structures or gradients. A redesign with fewer panels and larger maps would significantly enhance visual communication.
6.The manuscript lacks sufficient methodological detail on how city boundaries were defined and harmonized over the 1950–2020 period. Given the rapid urban expansion—especially in the Global South—using static boundaries may introduce systematic bias into long-term ETV trend estimates. This issue requires clearer documentation and discussion.
7.In lines 317–319, the spatial resolution of the datasets should be explicitly stated. Additionally, further clarification is needed regarding the accuracy, temporal coverage, and representativeness of the electricity-consumption data used.
8.Day-to-day temperature variability is calculated using a five-day rolling window. It is unclear whether this choice aligns with typical synoptic weather time scales or other considerations. The authors should justify this parameter selection and discuss the sensitivity of results to alternative window lengths.
In summary, while the manuscript addresses an important and timely topic, the current version does not meet the high bar for publication in Nature Communications. I hope these comments prove constructive. With substantial revisions—including clearer articulation of scope and limitations, stronger mechanistic and conceptual grounding, and improved data presentation—the study may find a better fit in a more specialized journal.

(Remarks on code availability)

Reviewer #2

(Remarks to the Author)
This manuscript presents a comprehensive global analysis of extreme temperature variability (ETV) across 10,522 cities from 1950 to 2020 using an interpretable machine-learning framework. By shifting the focus from mean temperature change to day-to-day variability and decomposing ETV into intensity and frequency components, the study makes a valuable and timely contribution to urban climate science. The identification of aerosols as a dominant urban factor and the finding that high-HDI cities experience intensifying ETV are particularly novel and policy-relevant. Overall, the data integration effort is impressive, the structure is clear, and the paper is suitable for publication after minor revision.

Several clarifications would substantially improve transparency and readability:
(1) the definitions and relationships among “annual mean ETV,” “event intensity,” and “event frequency” should be summarized in a unified framework (e.g., at the beginning of the Methods or Results) to avoid confusion between cumulative and single-event metrics.
(2) the numerous urbanization-related variables would benefit from a consolidated table summarizing their definitions, units, data sources, and theoretical motivations. The interpretation of blue–green infrastructure should also be clarified, as the current indicators capture average coverage rather than spatial connectivity, while the text sometimes implies the latter.
(3) the subsection title “Urbanization mitigates ETV” appears stronger than what the results support, given the documented divergence among cities and the intensification in high-HDI regions, and may warrant rephrasing.
(4) Regarding presentation, Figures 1, 2, and 4 are overly dense. Reducing the number of subpanels, clearly explaining all abbreviations, standardizing color bars, and moving secondary results to the Supplementary Information would improve readability.
(5) For the CMIP6 comparison in Figure 1, clarifying the time period and/or including future projections or multiple models would strengthen its relevance.
(6) A short paragraph summarizing the main take-home message and its concrete implications for urban climate governance would help bridge the technical analysis and policy discussion.
Subject to these revisions, the manuscript will be significantly strengthened.

(Remarks on code availability)
The code is generally well structured and the results appear reproducible in principle. However, in-line comments and documentation are limited. Adding more comments and a clearer README with installation and usage instructions would improve transparency and usability for the community.

Reviewer #3

(Remarks to the Author)
This manuscript presents an interesting study on extreme temperature variability (ETV) across global cities, offering a novel perspective on urban climate risk beyond mean temperature and conventional extremes. The large-scale dataset and the integration of urban factors with climate background provide valuable insights into the spatial heterogeneity and potential drivers of temperature variability. However, the following points still need further consideration, which will help further clarify the choice of methods and enhance the reliability and interpretability of the results.

This study proposes an interesting metric of Extreme Temperature Variability (ETV), which provides a useful perspective beyond mean temperature and traditional extremes. However, the construction of ETV, particularly the use of a 5-day rolling window and the 90th percentile threshold, would benefit from further justification. It is not entirely clear whether these parameter choices are theoretically grounded or empirically driven.

The decomposition of ETV into intensity and frequency is helpful for understanding temperature variability, and the finding that intensity dominates is insightful. However, it would be useful to briefly clarify the rationale for this decomposition and its practical or physical significance. It would also be helpful to indicate whether this framework is based on prior studies or newly proposed in this work.

The selection of urban indicators in this study is comprehensive and represents a clear strength of the paper. However, the rationale behind the selection of these variables is not yet entirely clear. It is recommended that the authors provide further clarification on the logic behind the choice of these indicators. For example, whether it is based on a theoretical framework, data availability, or empirical support from existing literature. Such explanations could be included in the Methods or Discussion sections to enhance the transparency of the analysis and the soundness of the interpretation of the results.

The use of machine learning is appropriate for capturing nonlinear relationships and provides useful interpretability. However, it would be helpful for the authors to further clarify why this approach was chosen over more traditional statistical or econometric models. In particular, what are the specific advantages of this framework in the context of this study? Furthermore, it should be noted that SHAP essentially reflects the association between variables and outcomes, rather than

a causal relationship; this point could be made clearer in the interpretation of the results.

The policy implications discussed in the manuscript are relevant and potentially valuable, especially regarding aerosol control and urban design strategies. However, since the analysis is primarily based on observational data and machine learning models, the results should be interpreted as associative rather than causal. Some of the policy recommendations may therefore need to be presented more cautiously. Strengthening the discussion on causality, such as acknowledging possible reverse causality or omitted variables, would further enhance the credibility of the conclusions.

I would encourage the authors to revise the manuscript and resubmit, as the study is interesting and has the potential to make a valuable contribution.

(Remarks on code availability)
It would be helpful if the authors could provide a README or clear documentation to facilitate code usability and reproducibility.

Version 1:

Reviewer comments:

Reviewer #1

(Remarks to the Author)
The authors have satisfactorily addressed my previous concerns and made substantial revisions to the manuscript. However, I believe that two issues still require clarification before publication.
1. The study argues that the newly proposed extreme temperature variability metric captures hazard-related information that is not adequately reflected by conventional temperature-extreme metrics. However, the manuscript does not clearly articulate the specific advantages of extreme temperature variability, nor does it explicitly explain which limitations of existing metrics it overcomes.
2. Although the authors claim that combining LightGBM and GAM provides significant advantages for identifying nonlinear relationships between urban characteristics and extreme temperature variability, the approach is fundamentally based on statistical and machine learning models. Therefore, it cannot fully escape the inherent limitations associated with black box modelling. The authors should explicitly acknowledge these limitations and discuss them in more detail. Additionally, outlining potential physics- or process-based modelling frameworks that could address these limitations in future research and provide a more mechanistic understanding of the observed relationships would be valuable.

(Remarks on code availability)
The code provides a README file that contains sufficient instructions for installing and running the application.

Reviewer #2

(Remarks to the Author)
The authors have adequately addressed my concerns. The manuscript now presents a clearer and more balanced contribution to urban climate science, and I believe it is suitable for publication.

(Remarks on code availability)
I have reviewed the provided code, and overall it appears to be well organized and usable. The code includes sufficient instructions for installation and execution, and it seems to provide a reasonable basis for reproducing the main results reported in the paper. In my view, the code can serve as a useful resource for the community.

Reviewer #3

(Remarks to the Author)
The author has addressed my comments.

(Remarks on code availability)
The author has improved the code's README.

Open Access This Peer Review File is licensed under a Creative Commons Attribution 4.0 International License, which permits use, sharing, adaptation, distribution and reproduction in any medium or format, as long as you give appropriate credit to the original author(s) and the source, provide a link to the Creative Commons license, and indicate if changes were made.
In cases where reviewers are anonymous, credit should be given to 'Anonymous Referee' and the source.
The images or other third party material in this Peer Review File are included in the article's Creative Commons license, unless indicated otherwise in a credit line to the material. If material is not included in the article's Creative Commons license and your intended use is not permitted by statutory regulation or exceeds the permitted use, you will need to obtain permission directly from the copyright holder.
To view a copy of this license, visit https://creativecommons.org/licenses/by/4.0/

Responses

No.: NCOMMS-25-104270-T

Urbanization processes widen global divergence in extreme temperature variability

Thank you for the comments and suggestions concerning our manuscript entitled: "Urbanization processes widen global divergence in extreme temperature variability" (No.: NCOMMS-25-104270-T). These comments are valuable in revising our paper and improving its quality.

In response, we have revised the manuscript extensively in line with your guidance by clarifying the city-level scale of inference, expanding methodological detail, redesigning key figures, softening causal claims, and strengthening the discussion of policy relevance and limitations. We now explicitly position the study as a global, city-scale assessment of weather-scale ETV, supported by additional robustness checks and a more cautious interpretation of urban-factor associations.

The amendments in the paper and a point-by-point response to the comments and suggestions are as follows. We believe that these revisions have substantially improved the manuscript's rigor, transparency, and interpretability, and we are grateful for your guidance in strengthening it. We trust that the revised paper meets your expectations.

<u>Responses to Reviewer #1's comments:</u>

This manuscript investigates extreme temperature variability (ETV) across over 10,000 cities worldwide over the past seven decades and applies interpretable machine-learning methods to identify potential drivers. The topic is timely and relevant to urban climate and heat mitigation research. However, despite the impressive global scope of the dataset, I find that the study falls short of the standards for Nature Communications and would be better suited for a specialized journal in ecology and environmental sciences.

<u>Response:</u>

We thank you for recognizing the timeliness and relevance of this study. We agree that, in the original submission, the broader significance of the work was not articulated with sufficient clarity. In response to your concern, we have revised the manuscript to strengthen its conceptual framing and clarify its broader implications.

Specifically, this study addresses a question of general relevance: how urbanization reshapes short-term climate risk across cities under diverse climatic and developmental contexts. Our results reveal a global divergence in the trajectories of ETV-related impacts between cities at different development levels. We further show that the recent increase in cumulative ETV is driven primarily by event intensity rather than event frequency, and that this divergence cannot be accounted for by climatic and geographic factors alone. In addition, by applying an interpretable machine-learning framework, we identify distinct associations of aerosols, blue–green infrastructure, and urban form with different dimensions of ETV risk. We therefore believe that these findings are relevant not only to urban climate research but also to the broader discussions of climate risk inequality, adaptation, and urban governance highlighted by Nature Communications.

We are grateful for your constructive feedback, which has helped us improve the manuscript, and we thank you again for your support in this work.

Corresponding modified content:

INTRODUCTION

……

Here, we show a global, city-level assessment of extreme temperature variability (ETV), defined as day-to-day differences in near-surface air temperature (see Methods). We compile ERA5-Land daily data from ECMWF and track 10,522 cities from 1950 to 2020. We harmonize these records with

climatic, geographic, urban-form, and socioeconomic covariates. We demonstrate that atmospheric circulation, land–sea thermal contrasts, and development levels collectively influence the spatial pattern and temporal evolution of ETV. For 2005–2019, when annual urban covariates are available, we integrate climatic, geographic, urban-form, and socioeconomic indicators and apply an interpretable machine-learning framework to assess the associations between city-level factors and ETV across climate zones and development stages. We show that ETV trajectories diverge by city development status, and that recent cumulative ETV is driven more by event intensity than by event frequency. Those urban features, such as aerosols, blue–green infrastructure, and surface properties, are associated with distinct dimensions of ETV. Together, these findings position ETV as an underexamined axis of urban climate-risk inequality and advance Sustainable Development Goal 11 and climate–health adaptation targets.

DISCUSSION

ETV captures an underexamined dimension of urban climate-risk inequality. Recent work suggests that abrupt day-to-day temperature swings constitute a hazard dimension largely independent of standard ETCCDI temperature-extreme indices across most land areas[4]. Our results extend this perspective to the urban domain by showing that ETV has its own global geography, temporal divergence, and set of urban correlates. Using a 1950–2020 panel of 10,522 cities, we show that city-level ETV follows a coherent global structure shaped jointly by latitude, land–sea thermal contrasts, and development status. High-latitude and inland cities experience the largest weather-scale day-to-day temperature swings, indicating that climatic background sets the baseline geography of variability. Yet these controls do not fully explain recent divergence across cities. Whereas many low-HDI cities show weak declines or relative stability, high-HDI cities continue to intensify, yielding a current gap of about 1.66 °C in ETV intensity.

By decomposing cumulative ETV into event frequency and single-event intensity, we further show that recent divergence across cities is driven primarily by intensity, especially at high latitudes and in high-HDI settings. This matters because a small number of large day-to-day swings can impose disproportionate burdens on health, infrastructure, and energy systems[24-28]. Therefore, the high-HDI may increase intra-urban inequality because vulnerable residents, especially those in dense areas with limited blue–green buffers, face elevated health and energy burdens[29,30]. In that sense, focusing on event amplitude, in addition to event occurrence, provides a more relevant basis for early-warning thresholds, infrastructure design, and preparedness planning.

Comment 1:

The analysis encompasses more than 10,000 cities, many of which are spatially small—often only a few square kilometers. The study primarily uses reanalysis data to characterize urban-scale ETV and to infer urbanization effects. Given the coarse spatial resolution of reanalysis products, it is unlikely they can adequately resolve genuine urban thermal signals at such fine scales, casting doubt on the robustness of

the inferred urbanization effects and overall validity of the results.

Response:

Thank you for raising this important concern. We agree that coarse-resolution reanalysis products cannot fully resolve neighborhood-scale urban thermal heterogeneity, and we acknowledge that the original manuscript did not clearly state the intended scale of analysis. However, we would like to clarify that our study does not include settlements with built-up areas of only a few square kilometers. The sample was restricted to cities with built-up areas of at least 10 km^2, and the urban-factor analysis was conducted only for this size-constrained sample.

Our objective is not to resolve urban heat signals directly from the reanalysis, but to characterize city-scale, weather-scale ETV. The ETV metric is derived from daily near-surface air temperature using a five-day rolling window and is intended to capture robust day-to-day thermal fluctuations. In this framework, the reanalysis data serve as a spatially consistent meteorological baseline for global comparisons, rather than as direct measurements of local urban thermal signals.

Importantly, our inference regarding urbanization is not based solely on the reanalysis product. In the subsequent analysis, after controlling for macro-climatic and geographic background factors such as latitude, distance to the coast, elevation, wind speed, precipitation, and downward surface shortwave radiation, we linked city-level ETV metrics to a set of independent, higher-resolution urban descriptors, including $PM_{2.5}$, population density, electricity consumption, urban morphology, vegetation, and water.

We agree that the phrase "urbanization effects" may overstate the spatial precision and causal strength of the analysis. In the revised manuscript, we have adopted more cautious wording, such as "urbanization-related associations" or "urban factors associated with ETV," rather than implying that the reanalysis directly resolves all urban thermal signals or supports strict causal inference.

In addition, we performed a cross-product robustness check using CMIP6 EC-Earth3 with the same urban units and the same ETV definition. This analysis reproduced the key coastal–inland contrast and the intensification pattern in high-latitude inland regions, indicating that the main patterns reported in the manuscript are robust at the large-scale, city level rather than being artifacts of a single gridded product.

Accordingly, we have revised the manuscript to clarify the scale of inference, state the city-size threshold and its rationale more explicitly, and expand the limitations section to acknowledge the potential effects

of grid–city scale mismatch.

Corresponding modified content:

Urban factors associated with ETV

The overall magnitude of urban ETV has decreased, yet the gaps between cities have widened. Climate and geography account for much of the baseline spatial pattern, but they cannot explain this pattern of "mean convergence with stretched extremes." Urbanization processes are driving the divergence16. The pace of surface sealing, the strength of sensible heat emissions, the extent of blue–green infrastructure, and block-scale morphology that governs ventilation and heat storage are reshaping local ETV trends[20-22]. To assess whether city-level urban characteristics are associated with this divergence, we developed an interpretable machine-learning framework for the 2005–2019 period using 10,522 cities with built-up areas exceeding 10 km^2, the sample for which annual urban descriptors are available. After controlling for macro-climatic and geographic background, five urban factors, including aerosol concentration, anthropogenic heat, urban morphology, blue–green infrastructure, and surface albedo, explain 30.83% of the spatiotemporal variation in ETV intensity and 48.85% in ETV frequency. The models achieve R^2 values of 0.97 for intensity and 0.84 for frequency. Crucially, the association patterns of these factors differ significantly between intensity and frequency, indicating distinct levers for risk management. Sensitivity analyses based on GAMs and 3/7-day rolling windows are provided in Supplementary Fig. 1, 2, 3, 4, 5, 6, and 7.

DISCUSSION

……

Finally, we acknowledge several limitations and outline the next steps. Our analysis is designed for the city scale. Because ETV is derived from gridded daily near-surface air temperature, the dataset cannot fully resolve neighborhood- or canopy-scale thermal heterogeneity. Our conclusions should therefore be interpreted as city-level patterns rather than direct evidence of street-scale urban heat processes. Second, the next-step research should employ counterfactual designs or instrumental-variable strategies to disentangle the roles of emissions, morphology, and meteorology, clarify causal effects, and strengthen threshold estimates for the urban-process variable. Finally, future research should include future projections from CMIP6 models to assess trends in ETV across different scenarios. Despite these limitations, our harmonized analysis of 10,522 cities shows that urbanization is reshaping the intensity and frequency structure of temperature risk. By disentangling how urbanization processes interact with the climatic background, we outline a practical path to steer cities out of the high-frequency, large-amplitude tail of risk and back into a safer operating space.

METHODS

……

Urbanization-related Indicators

To systematically characterize urbanization in relation to ETV, the selection of urban indicators was guided by an urban surface energy-balance framework, in which cities influence the thermal environment through five major pathways: radiative forcing, anthropogenic heat input, aerodynamic and heat-storage effects of urban form, hydrological buffering by vegetation and water, and surface

reflectance14. Therefore, we compiled five categories of raster-based indicators with global coverage and annual observations for 2005-2019, the period for which urban covariates are consistently available. For each year, these variables were aggregated to city-level means within each city's contemporaneous built-up footprint and then used in subsequent urban-factor analyses. City boundaries were derived from annually updated built-up extents based on multi-source remote-sensing products40. Because our analysis targets city-level, weather-scale ETV rather than neighborhood-scale thermal heterogeneity, and to reduce the mismatch between dynamic urban polygons and the underlying meteorological grid, we retained only cities with a built-up area of at least 10 km^2 during the urban-factor analysis period. This filter excludes very small settlements and improves the global comparability of both urban extent and urban descriptors. A total of 10,522 cities met these criteria and were included in the analysis. Note that ETV itself was calculated for 1950-2020, whereas analyses linking ETV to urbanization-related factors were restricted to 2005-2019 owing to data availability.

METHODS

……

Empirical Modelling Approach

To effectively reveal the complex nonlinear interactions between urban characteristics and ETV, this study combines gradient boosting decision trees (LightGBM) and the Generalized Additive Model (GAM) to address our core research question[46-49]. LightGBM is used to capture nonlinear effects and high-order interactions, quantify feature importance, and visualize local response patterns, whereas GAM serves as a complementary semi-parametric framework to assess the statistical significance, functional direction, and robustness of the identified associations.

Two separate sets of models are constructed using ETV_Int. and ETV_Fre. as dependent variables, thereby capturing different dimensions of urban influence on ETV. Influencing factors are divided into two distinct categories: explanatory urban feature variables and background control variables. Urban feature variables, including $PM_{2.5}$, POP_PA, EC_PA, BD, BH, FAR, CI, EVI, WD, and FAL, are introduced to characterize direct impacts on ETV arising from human activities, spatial morphology, and ecological infrastructure. Background control covariates, including Lat, Ocean_D, DEM, WS, TP, and DSSR, were incorporated to account for broad-scale geographic and climatic conditions and thereby reduce confounding.

Comment 2:

The finding that ETV is higher in mid- to high-latitude regions and continental interiors compared to low-latitude and coastal areas is largely expected, as it reflects background climatic variability. Attributing these large-scale patterns primarily to urban development introduces a conceptual gap. Moreover, ETV involves complex physical mechanisms related to surface energy balance, atmospheric circulation, and radiative processes. The manuscript relies almost exclusively on explanatory

machine-learning models without adequate theoretical grounding or mechanistic validation, leaving the conclusions largely correlational and vulnerable to “black-box” interpretation.

Response:

We thank you for this important comment. We agree that the higher ETV observed in mid to high-latitude and inland cities is primarily a first-order expression of background climatic variability, and we did not intend to attribute this baseline spatial pattern mainly to urban development. Rather, our point is that climate and geography define the broad global envelope of ETV, whereas development status and urban characteristics help explain the divergence among cities after controlling for macro-climatic and geographic background. We have revised the manuscript to make this distinction explicit and to avoid overstating the role of urbanization in shaping the global gradient.

We also agree that explanatory machine-learning models alone do not establish causality. Our analysis is therefore intended as a theory-informed, global assessment of city-level associations rather than a fully process-resolving attribution. The LightGBM models were constructed with physically motivated predictors representing aerosol, radiative effects, anthropogenic heat, morphology, ecology, and albedo, and they show strong performance at the global scale ($R^2 = 0.97$ for ETV intensity and 0.84 for ETV frequency). To further reduce concerns about “black-box” interpretation, we additionally conducted generalized additive model (GAM) analyses. The GAM results show that both the urban features and the background controls are jointly significant, and the estimated response patterns are consistent with the trends identified by the LightGBM-SHAP analysis. This cross-model consistency increases our confidence. We have revised the text accordingly and now frame these results more cautiously as physically grounded and globally robust associations, rather than definitive causal mechanisms.

**Corresponding modified content:**
**RESULT**<br>**Urban factors associated with ETV**<br>The overall magnitude of urban ETV has decreased, yet the gaps between cities have widened. Climate and geography account for much of the baseline spatial pattern, but they cannot explain this pattern of "mean convergence with stretched extremes." Urbanization processes are driving the divergence16. The pace of surface sealing, the strength of sensible heat emissions, the extent of blue - green infrastructure, and block-scale morphology that governs ventilation and heat storage are reshaping local ETV trends[20-22]. To assess whether city-level urban characteristics are associated with this

divergence, we developed an interpretable machine-learning framework for the 2005–2019 period using 10,522 cities with built-up areas exceeding 10 km², the sample for which annual urban descriptors are available. After controlling for macro-climatic and geographic background, five urban factors, including aerosol concentration, anthropogenic heat, urban morphology, blue–green infrastructure, and surface albedo, explain 30.83% of the spatiotemporal variation in ETV intensity and 48.85% in ETV frequency. The models achieve R^2 values of 0.97 for intensity and 0.84 for frequency. Crucially, the association patterns of these factors differ significantly between intensity and frequency, indicating distinct levers for risk management. The association patterns of climatic and geographic background, sensitivity analyses based on GAMs, and 3/7-day rolling windows are provided in Supplementary Fig. 1, 2, 3, 4, 5, 6, and 7.

DISCUSSION

ETV captures an underexamined dimension of urban climate-risk inequality. Recent work suggests that abrupt day-to-day temperature swings constitute a hazard dimension largely independent of standard ETCCDI temperature-extreme indices across most land areas[4]. Our results extend this perspective to the urban domain by showing that ETV has its own global geography, temporal divergence, and set of urban correlates. Using a 1950–2020 panel of 10,522 cities, we show that city-level ETV follows a coherent global structure shaped jointly by latitude, land–sea thermal contrasts, and development status. High-latitude and inland cities experience the largest weather-scale day-to-day temperature swings, indicating that climatic background sets the baseline geography of variability. Yet these controls do not fully explain recent divergence across cities. Whereas many low-HDI cities show weak declines or relative stability, high-HDI cities continue to intensify, yielding a current gap of about 1.66 °C in ETV intensity.

By decomposing cumulative ETV into event frequency and single-event intensity, we further show that recent divergence across cities is driven primarily by intensity, especially at high latitudes and in high-HDI settings. This matters because a small number of large day-to-day swings can impose disproportionate burdens on health, infrastructure, and energy systems[24-28]. Therefore, the high-HDI may increase intra-urban inequality because vulnerable residents, especially those in dense areas with limited blue–green buffers, face elevated health and energy burdens[29,30]. In that sense, focusing on event amplitude, in addition to event occurrence, provides a more relevant basis for early-warning thresholds, infrastructure design, and preparedness planning.

METHODS

……

Empirical Modeling Approach

To effectively reveal the complex nonlinear interactions between urban characteristics and ETV, this study combines gradient boosting decision trees (LightGBM) and the Generalized Additive Model (GAM) to address our core research question[46-49]. LightGBM is used to capture nonlinear effects and high-order interactions, quantify feature importance, and visualize local response patterns, whereas GAM serves as a complementary semi-parametric framework to assess the statistical significance, functional direction, and robustness of the identified associations.

Two separate sets of models are constructed using ETV_Int. and ETV_Fre. as dependent variables, thereby capturing different dimensions of urban influence on ETV. Influencing factors are divided into two distinct categories: explanatory urban feature variables and background control variables. Urban feature variables, including $PM_{2.5}$, POP_PA, EC_PA, BD, BH, FAR, CI, EVI, WD, and FAL, are introduced to characterize direct impacts on ETV arising from human activities, spatial morphology, and ecological infrastructure. Background control covariates, including Lat, Ocean_D, DEM, WS, TP, and DSSR, were incorporated to account for broad-scale geographic and climatic conditions and thereby reduce confounding.

During model training and optimization, we apply Bayesian optimization (BO), leveraging probabilistic surrogate models to approximate the complex relationship between LightGBM hyperparameters and model performance[50]. This approach enables rapid and efficient hyperparameter tuning, significantly reducing computational load, ensuring efficient convergence to optimal solutions within a complex parameter space, and enhancing model prediction performance and stability.

To enhance the robustness of the machine learning results and address inherent challenges related to interpretability, this study integrates the Shapley Additive Explanations (SHAP) algorithm and Monte Carlo Simulation (MCS) to systematically reveal feature importance and their nonlinear effects on ETV. SHAP was used to generate local explanations for the fitted LightGBM models by quantifying each feature's contribution to individual predictions, thereby enabling both global importance ranking and visualization of local nonlinear associations with ETV[51]. Because SHAP values are conditional on the fitted predictive model, they are interpreted here as model-based associations rather than causal effects. To assess the stability of these interpretations, we conducted 500 independent MCS runs to capture uncertainty arising from repeated model fitting. Subsequently, the SHAP values obtained from all simulations are aggregated, constructing robust distributions of feature importance and reinforcing the reliability of model interpretation[52]. Finally, to quantitatively characterize the nonlinear relationships between each feature and ETV, we employ restricted cubic spline (RCS) fitting to the aggregated SHAP-based partial dependence plots, thereby clearly delineating the marginal association patterns and trend dynamics of different variables with respect to ETV.

As a complementary validation, we further fit GAMs for ETV_Int. and ETV_Fre. to evaluate whether the nonlinear associations identified by LightGBM-SHAP remain statistically significant under a more transparent semi-parametric specification.

$$ETV = \beta_0 + \sum_{j=1}^{p} f_j(x_{ij}) + \varepsilon \quad (6)$$

where x_{ij} denotes the set of urban feature variables and background control variables. Because the relationships between explanatory variables and ETV may be strongly nonlinear, all continuous predictors enter the model as smooth functions $f_j(\cdot)$. As both ETV_Int. and ETV_Fre. are treated as annual city-level continuous outcomes, the GAMs are fitted using a Gaussian family with an identity link. To reduce the influence of extreme values and improve model stability, the strongly right-skewed variables POP_PA and EC_PA are transformed using log1p prior to model fitting. The fitted smooth terms are used to evaluate the direction, shape, and statistical significance of each association, and the estimated response curves are compared with the LightGBM-SHAP results to assess cross-model

consistency.

SUPPLEMENTARY INFORMATION

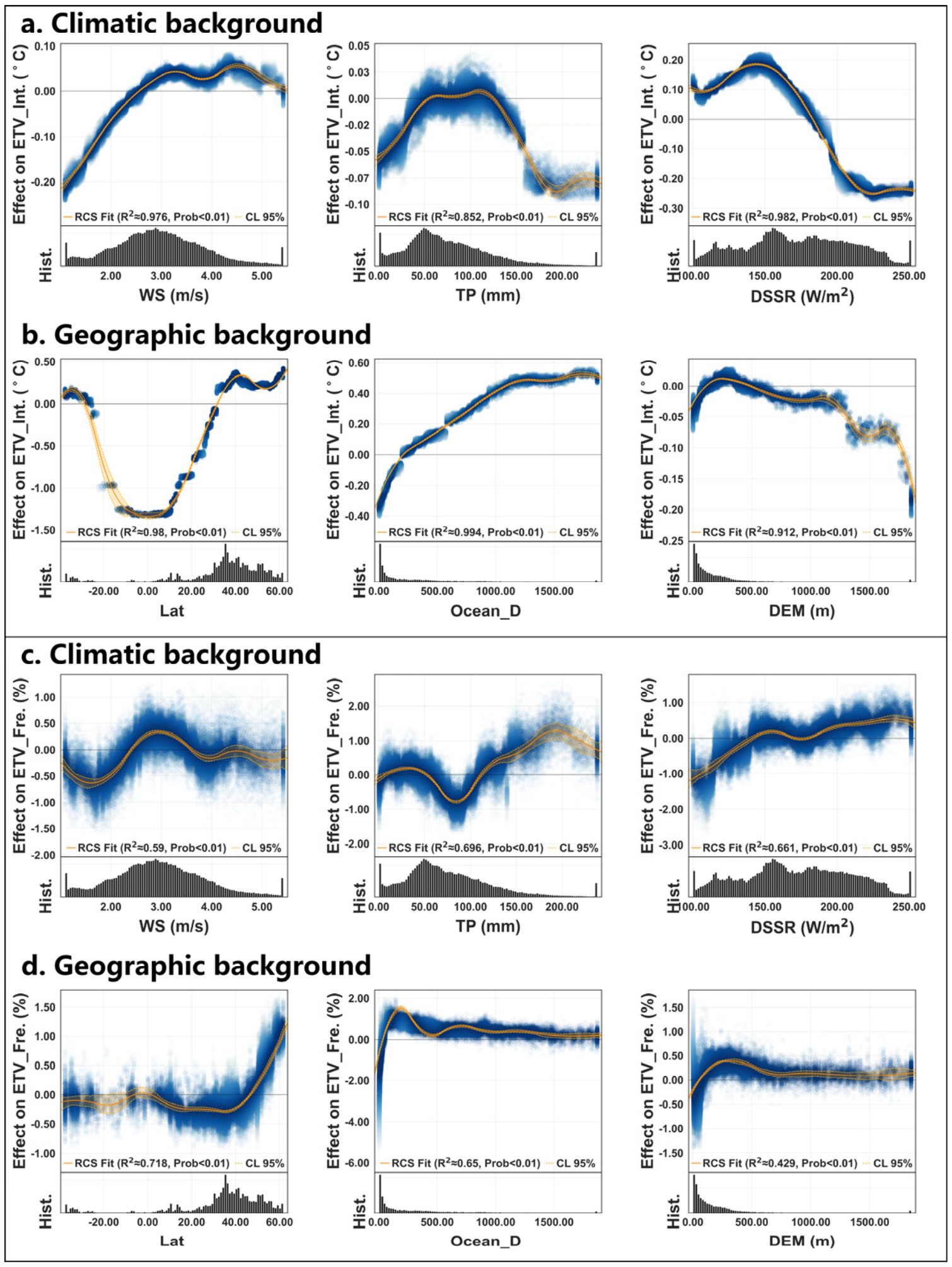

Supplementary Fig. 1 **The effect of climatic and geographic background features on ETV**. Local effect of each feature included in ensemble learning models on ETV_Int. (a and b) and ETV_Fre. (c and d) (n=157,830).

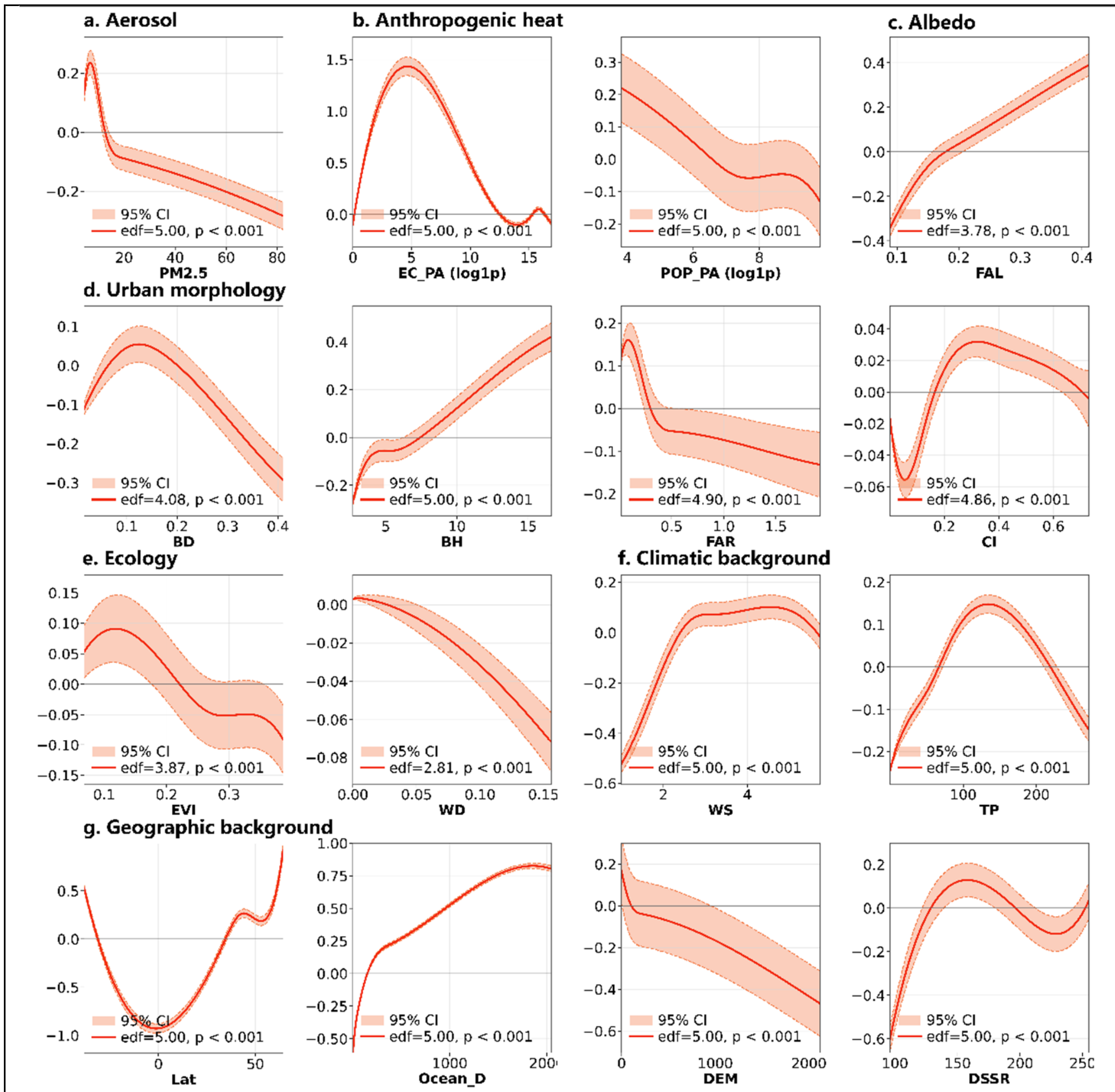

Supplementary Fig. 2 **The GAM results on ETV_Int.** Smooth effect curve and statistically significant level of each feature included in GAM on ETV_Int.(n=157,830).

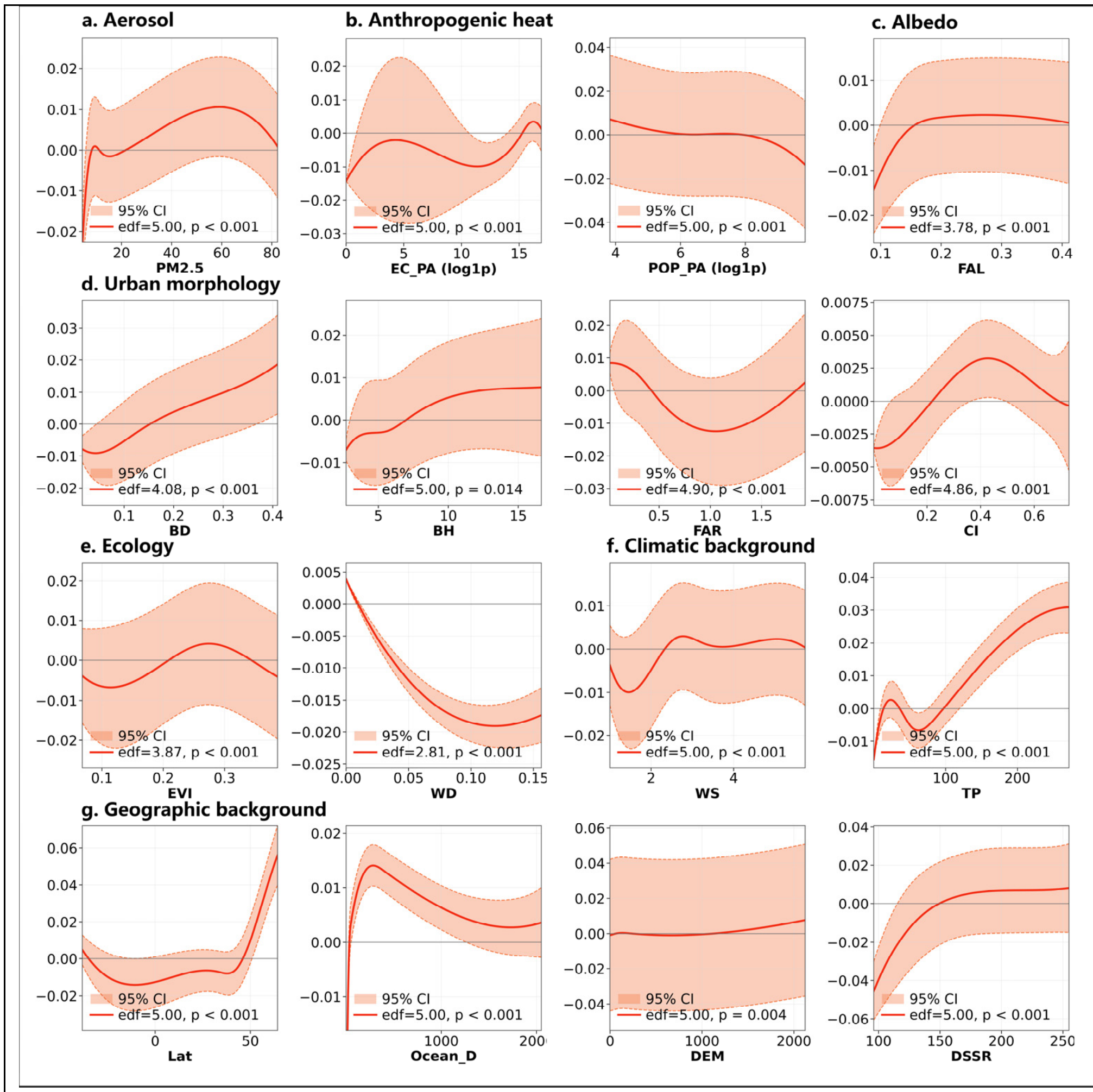

Supplementary Fig. 3 **The GAM results on ETV_Fre.** Smooth effect curve and statistically significant level of each feature included in GAM on ETV_Fre.(n=157,830).

Comment 3:

The discussion and policy implications remain underdeveloped. The core conclusions—that aerosols and blue-green infrastructure (BGI) influence temperature variability—are already well-established in urban climate literature. Thus, the study does not offer substantial conceptual advancement or a new theoretical framework; rather, it provides a large-scale quantitative synthesis of previously recognized relationships. Although the authors briefly suggest interventions such as aerosol reduction, expanded BGI, and improved urban form, these recommendations lack empirical validation or scenario-based assessment, limiting the study's utility for actionable urban climate governance.

Response:

We appreciate your concern that some of the individual urban controls discussed here, such as aerosols and BGI, have been considered previously in the urban climate literature. We apologize that the original manuscript did not make our intended contribution sufficiently clear. The conceptual contribution of this study is not to re-identify these factors in isolation, but to regard ETV as a unique dimension of climate risk and to examine its global variability and its correlation with urban factors within a unified framework. A recent work in *Nature Climate Change* on extreme day-to-day temperature changes (DTDT), a closely related weather-scale metric, shows that these extremes are largely independent of 15 ETCCDI temperature-related extreme indices across nearly all global land areas (Liu, Q., Fu, C., Xu, Z. & Ding, A. Global warming intensifies extreme day-to-day temperature changes in mid–low latitudes. Nature Climate Change 16, 69–76 (2026). https://doi.org/10.1038/s41558-025-02486-9). This supports the view that abrupt temperature swings represent a distinct hazard dimension not captured by conventional temperature-extreme metrics.

Building on this distinct and long-overlooked dimension of extreme risk, our study extends the analysis to the urban domain by quantifying city-level ETV, decomposing ETV into event intensity and event frequency, and comparing the relative associations of multiple urban factors under different climatic and development contexts. In this sense, the manuscript is intended as a comparative risk diagnosis and prioritization framework, rather than a direct intervention evaluation study. In addition, the pronounced spatial heterogeneity in local sensitivities indicates that the same measure is unlikely to perform uniformly across cities. We therefore interpret aerosol control, BGI, and morphology-sensitive design not as universally validated prescriptions, but as management-relevant directions.

Because the present study is observational and retrospective, it identifies robust associations after controlling for major climatic and geographic backgrounds, but it does not quantify policy effects. Future work should therefore determine which interventions reduce ETV, for whom, and under what climatic and developmental conditions. We have revised the Introduction and Discussion to more clearly distinguish between empirically supported associations and intervention efficacy, and to frame the study's policy relevance as identifying priority risk dimensions and candidate management directions for local validation, rather than providing scenario-tested prescriptions.

**Corresponding modified contents:**
**INTRODUCTION**<br>Global climate change and rapid urbanization are reshaping daily life worldwide. Human-driven warming not only raises average temperatures but also shifts temperature variability and the frequency and intensity of extremes[1]. Short-term temperature variability (TV) is now recognized as a critical risk, as people and systems have little time to adapt[2,3]. Recent evidence further suggests that abrupt day-to-day temperature swings constitute a distinct hazard dimension that is largely independent of conventional ETCCDI temperature-extreme indices across almost all global land areas[4]. Sudden swings in temperature strain health services, disrupt energy demand, and stress ecosystems[5-7]. The World Health Organization (WHO) reports that frequent temperature fluctuations exacerbate heat stress and heat-related illnesses, thereby increasing the risk for urban residents. Urban TV, therefore, matters for public health, reliable energy systems, and ecological stability. It also matters for progress toward the United Nations' Sustainable Development Goals (SDGs), specifically those related to sustainable cities and human well-being.<br>……<br>Here, we show a global, city-level assessment of extreme temperature variability (ETV), defined as day-to-day differences in near-surface air temperature (see Methods). We compile ERA5-Land daily data from ECMWF and track 10,522 cities from 1950 to 2020. We harmonize these records with climatic, geographic, urban-form, and socioeconomic covariates. We demonstrate that atmospheric circulation, land – sea thermal contrasts, and development levels collectively influence the spatial pattern and temporal evolution of ETV. For 2005 – 2019, when annual urban covariates are available, we integrate climatic, geographic, urban-form, and socioeconomic indicators and apply an interpretable machine-learning framework to assess the associations between city-level factors and ETV across climate zones and development stages. We show that ETV trajectories diverge by city development status, and that recent cumulative ETV is driven more by event intensity than by event frequency. Those urban features, such as aerosols, blue – green infrastructure, and surface properties, are associated with distinct dimensions of ETV. Together, these findings position ETV as an underexamined axis of urban climate-risk inequality and advance Sustainable Development Goal 11 and climate – health adaptation targets.
**DISCUSSION**<br>ETV captures an underexamined dimension of urban climate-risk inequality. Recent work suggests that abrupt day-to-day temperature swings constitute a hazard dimension largely independent of standard ETCCDI temperature-extreme indices across most land areas[4]. Our results extend this perspective to the urban domain by showing that ETV has its own global geography, temporal divergence, and set of urban correlates. Using a 1950 – 2020 panel of 10,522 cities, we show that city-level ETV follows a coherent global structure shaped jointly by latitude, land – sea thermal contrasts, and development status. High-latitude and inland cities experience the largest weather-scale day-to-day temperature swings, indicating that climatic background sets the baseline geography of variability. Yet these controls do not fully explain recent divergence across cities. Whereas many low-HDI cities

show weak declines or relative stability, high-HDI cities continue to intensify, yielding a current gap of about 1.66 ° C in ETV intensity.

By decomposing cumulative ETV into event frequency and single-event intensity, we further show that recent divergence across cities is driven primarily by intensity, especially at high latitudes and in high-HDI settings. This matters because a small number of large day-to-day swings can impose disproportionate burdens on health, infrastructure, and energy systems[24-28]. Therefore, the high-HDI may increase intra-urban inequality because vulnerable residents, especially those in dense areas with limited blue－green buffers, face elevated health and energy burdens[29,30]. In that sense, focusing on event amplitude, in addition to event occurrence, provides a more relevant basis for early-warning thresholds, infrastructure design, and preparedness planning.

Aerosols are often treated as a by-product of urban growth[31]. Using data from more than 10,000 cities, we find that aerosols emerge as the leading predictor of ETV in our models. Their relative model importance has increased over the past 15 years and is about four times that of BGI. At low to moderate concentrations, shortwave scattering reduces daytime absorption, speeds the post-event cooldown, and decreases single-event variability in size[32,33]. At higher concentrations, especially where anthropogenic heat is large, enhanced nighttime longwave re-emission raises near-surface thermal inertia[34]. Warm anomalies then decay more slowly, and ETV intensity increases. We identify a global pivot near 20 $\mu g\ m^{-3}$. Beyond this level, the damping benefit quickly fades and can even reverse. Even so, these results remain associational rather than causal, due to $PM_{2.5}$ may covary with unobserved factors such as fuel structure, traffic intensity, building operations, or boundary-layer conditions. In addition, urban form and ventilation determine how quickly heat is removed[35]. A higher floor area ratio increases effective heat capacity and dampens both warming and cooling[36]. Taller buildings and more compact layouts, particularly under background winds or frontal passages, can enhance near-surface turbulence and short-term heat exchange[16]. From a policy perspective, this links air-quality management to thermal-volatility management. $PM_{2.5}$ control remains a no-regret option because it yields immediate health benefits and may also help moderate ETV in polluted, energy-intensive settings, especially when paired with albedo management and ventilation-sensitive design.

BGI remains consistently negatively associated with ETV, but its contribution is smaller and more context-dependent than that of its well-established role in reducing mean heat exposure[16,22,26,37]. This suggests that ecological infrastructure can buffer thermal volatility, but its effectiveness depends strongly on surrounding urban form, ventilation, and climatic background. Morphology-related variables also show mixed signs, highlighting trade-offs among heat storage, roughness, shading, and turbulent exchange[16,37]. The policy implication is therefore a place-based portfolio approach rather than a one-size-fits-all intervention logic. High-pollution, high-density cities may benefit most from integrated packages combining emissions reduction, reflective-surface or envelope upgrades, ventilation corridors, and connected blue－green networks. Cities with already strong ecological bases may gain more from protecting continuity, reducing fragmentation, and targeting heat-vulnerable districts. Rapidly urbanizing cities may reduce future ETV lock-in by embedding ecological continuity, street ventilation, and surface-property management early in land-use planning[38,39].

More broadly, the value of this analysis lies less in prescribing a universal intervention package than in informing policy sequencing and regional prioritization. In South Asia and East Asia, where heavy pollution is associated with a greater risk of fluctuations, air-quality control should be paired with albedo management and ventilation-sensitive design. In dense urban corridors of the Middle East and the United States, morphology-sensitive retrofits and ventilation corridors may be more effective than green expansion alone. By contrast, cities in Western Europe, Eastern Europe, and the eastern United States may gain more from protecting and connecting existing blue–green networks. Because the present study is observational and retrospective, these directions should be interpreted as management-relevant rather than as causal or scenario-tested prescriptions.

Finally, we acknowledge several limitations and outline the next steps. Our analysis is designed for the city scale. Because ETV is derived from gridded daily near-surface air temperature, the dataset cannot fully resolve neighborhood- or canopy-scale thermal heterogeneity. Our conclusions should therefore be interpreted as city-level patterns rather than direct evidence of street-scale urban heat processes. Second, the next-step research should employ counterfactual designs or instrumental-variable strategies to disentangle the roles of emissions, morphology, and meteorology, clarify causal effects, and strengthen threshold estimates for the urban-process variable. Finally, future research should include future projections from CMIP6 models to assess trends in ETV across different scenarios. Despite these limitations, our harmonized analysis of 10,522 cities shows that urbanization is reshaping the intensity and frequency structure of temperature risk. By disentangling how urbanization processes interact with the climatic background, we outline a practical path to steer cities out of the high-frequency, large-amplitude tail of risk and back into a safer operating space.

149

Comment 4:

The heatmaps in Fig. 1a and 1b, intended to illustrate interannual ETV variability across regions, suffer from poor visual clarity. The regional subdivision is overly granular, making it difficult to discern coherent patterns. A coarser regional classification or alternative visualization approach would improve interpretability.

Response:

We thank you for this constructive suggestion. We agree that the original heatmaps were visually too dense to clearly reveal coherent regional patterns. In the revised Fig. 1, we improved readability by redesigning the heatmap presentation and assigning distinct color bands to individual geographic subregions, which increases visual contrast within each subregion while preserving the overall large-scale pattern.

Corresponding modified content:

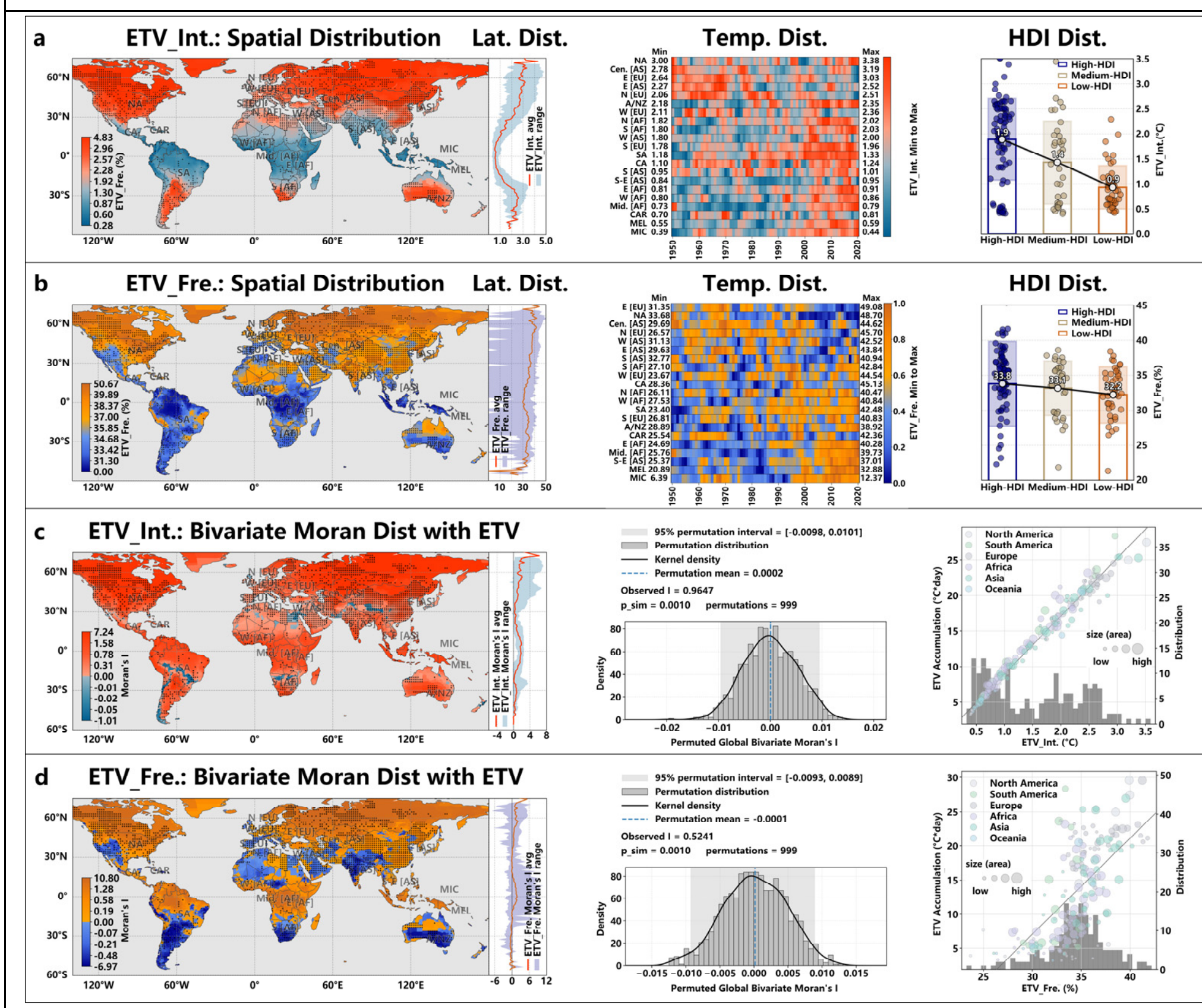

Fig. 1. **Spatio-temporal distribution of ETV.**

Comment 5:

In Fig. 4, the individual global maps are presented at too small a scale to allow meaningful interpretation. The multi-panel layout hinders the reader's ability to identify spatial structures or gradients. A redesign with fewer panels and larger maps would significantly enhance visual communication.

Response:

Thank you for your helpful suggestion. The original multi-panel layout of Fig. 4 made the global sensitivity patterns difficult to interpret. In response, we have redesigned Fig. 4 to improve visual communication. Specifically, we aggregated the original results to the second-level administrative unit from GADM, thereby reducing visual fragmentation and making regional patterns easier to identify. We also aligned the latitude–longitude axes across panels and enlarged the map panels, allowing readers to compare spatial gradients more directly across variables. We believe that these changes substantially improve the readability of Fig. 4 and the visibility of the main spatial patterns.

Corresponding modified content:

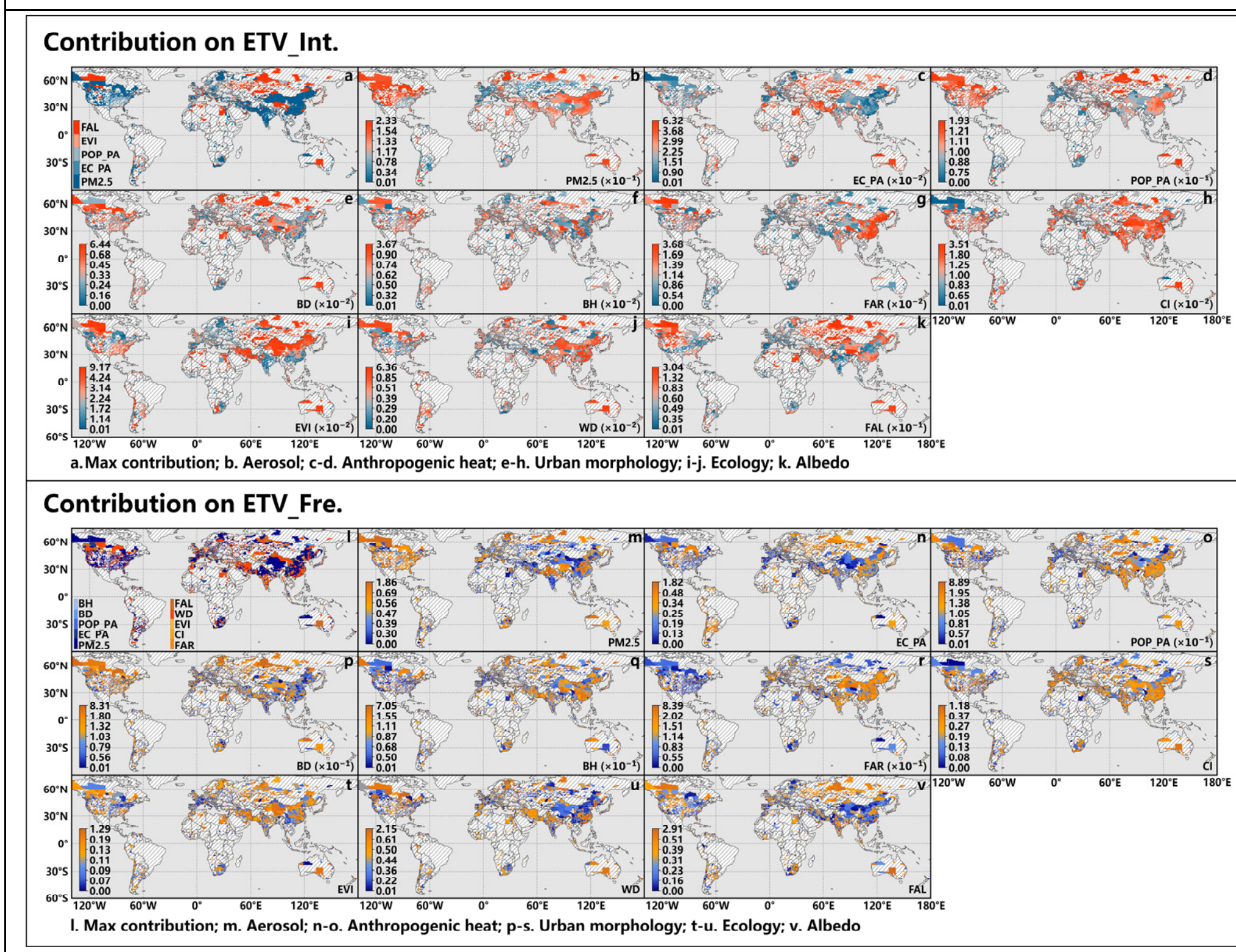

Fig. 4. **The sensitivity of urban factors on ETV.**

Comment 6:

The manuscript lacks sufficient methodological detail on how city boundaries were defined and harmonized over the 1950–2020 period. Given the rapid urban expansion—especially in the Global South—using static boundaries may introduce systematic bias into long-term ETV trend estimates. This issue requires clearer documentation and discussion.

Response:

We thank you for raising this important point. We agree that the definition and temporal harmonization of city boundaries should be described more clearly, especially in a long-term analysis spanning 1950–2020. We would like to clarify that our study does not rely on a fully static urban boundary for the entire period. Instead, city boundaries were constructed by integrating annual built-up extents derived from multi-source remote-sensing imagery with second-level administrative units from the GADM database. For the period with annual built-up data availability, the urban extent was therefore treated dynamically rather than as a fixed footprint.

Corresponding modified content:

METHODS

……

Urbanization-related Indicators

To systematically characterize urbanization in relation to ETV, the selection of urban indicators was guided by an urban surface energy-balance framework, in which cities influence the thermal environment through five major pathways: radiative forcing, anthropogenic heat input, aerodynamic and heat-storage effects of urban form, hydrological buffering by vegetation and water, and surface reflectance[14]. Therefore, we compiled five categories of raster-based indicators with global coverage and annual observations for 2005-2019, the period for which urban covariates are consistently available. For each year, these variables were aggregated to city-level means within each city's contemporaneous built-up footprint and then used in subsequent urban-factor analyses. City boundaries were derived from annually updated built-up extents based on multi-source remote-sensing products[40]. Because our analysis targets city-level, weather-scale ETV rather than neighborhood-scale thermal heterogeneity, and to reduce the mismatch between dynamic urban polygons and the underlying meteorological grid, we retained only cities with a built-up area of at least 10 km^2 during the urban-factor analysis period. This filter excludes very small settlements and improves the global comparability of both urban extent and urban descriptors. A total of 10,522 cities met these criteria and were included in the analysis. Note that ETV itself was calculated for 1950-2020, whereas analyses linking ETV to urbanization-related factors were restricted to 2005-2019 owing to data availability.

Comment 7:

In lines 317–319, the spatial resolution of the datasets should be explicitly stated. Additionally, further clarification is needed regarding the accuracy, temporal coverage, and representativeness of the electricity-consumption data used.

Response:

We thank you for this helpful comment. In the revised manuscript, we now specify that elevation was derived from the DEM at 15-arc-seconds resolution. We also clarify that the electricity-consumption data are annual 1 km × 1 km gridded estimates for 1992–2019, derived from calibrated nighttime-light observations using a PSO-BP top-down framework tied to electricity statistics. The source study reports very high national-level agreement between predicted and observed normalized electricity consumption, supporting its utility for broad spatial comparisons, but we also acknowledge that it is an indirect proxy and may not fully capture sectoral structure, end-use composition, or short-term local variability. We therefore use this dataset as a city-level proxy for anthropogenic heat intensity rather than as a direct measurement of electricity demand. The revised manuscript now clarifies its spatial resolution, temporal coverage, and representativeness, and explicitly notes this limitation in the Methods.

**Corresponding modified content:**
**Urbanization-related Indicators**<br>……<br>Anthropogenic heat emissions constitute explicit urban heat sources, primarily arising from sensible heat fluxes associated with human metabolism, daily activities, and energy production. We characterized anthropogenic heat using two complementary metrics: population density per unit area (POP_PA) and electricity consumption per unit area (EC_PA). Population data were derived from the WorldPop global population raster dataset, calibrated using official statistics from the United Nations Population Division, with a spatial resolution of 1 km at the equator[42]. Electricity consumption data were obtained from a global annual 1 km × 1 km gridded dataset for 1992 – 2019, based on calibrated nighttime light observations and a top-down estimation framework linked to national electricity statistics[43]. In this study, EC_PA was used as a city-level proxy for anthropogenic heat intensity. Because this dataset is model-derived, it is suitable for representing broad spatial differences in electricity use, but it may not fully capture sector-specific demand structure, end-use heterogeneity, or short-term local variation.<br>……<br>**Climatic and Geographical Background**<br>This study obtained total precipitation (TP), wind speed (WS), and downward surface shortwave

radiation (DSSR) from the ERA5-Land dataset. These climate variables, available as daily time series at 0.1° spatial resolution, were aggregated to the city scale by computing mean values across each city's grid cells. Additionally, elevation data were derived from the Shuttle Radar Topography Mission (SRTM) Digital Elevation Model (DEM) at 15-arc-seconds resolution[45]. Ocean distance (Ocean_D) was calculated as the distance from each city's center to the nearest coastline.

Comment 8:

Day-to-day temperature variability is calculated using a five-day rolling window. It is unclear whether this choice aligns with typical synoptic weather time scales or other considerations. The authors should justify this parameter selection and discuss the sensitivity of results to alternative window lengths.

Response:

We thank you for this important comment. In this study, the rolling window was introduced to smooth day-to-day temperature differences and to capture weather-scale thermal fluctuations rather than isolated single-day noise. We selected a five-day window because it is broadly consistent with the characteristic time scale of synoptic weather variability and provides a balanced representation of short-term temperature swings without being overly sensitive to transient daily anomalies.

To address this concern, we have revised the Methods section to clarify the rationale for this parameter choice. In addition, we conducted sensitivity analyses using alternative rolling windows of 3 and 7 days. Specifically, we recomputed the ETV metrics and repeated the corresponding modeling analyses under both alternative specifications. The results show that the main spatial patterns and the core conclusions of the study remain qualitatively consistent across window lengths, indicating that our findings are not driven by the particular choice of a five-day window. We now report these additional results in the revised manuscript and refer to them in the Methods/Results as robustness checks. We hope this clarification addresses the reviewer's concern.

Corresponding modified content:

METHODS

Extreme Temperature Variability

In this study, the city is treated as the spatial unit of analysis with second-level administrative units from the GADM global database, and annual indicators from 1950 to 2020 are constructed to characterize city-level ETV. To quantify day-to-day temperature variability, we calculated daily temperature differences and smoothed these differences using a five-day rolling window. This window

was chosen to represent weather-scale thermal fluctuations associated with synoptic variability while reducing sensitivity to isolated single-day noise. To assess the robustness of this choice, we additionally repeated the analysis using 3-day and 7-day rolling windows, and the main results remained qualitatively consistent across specifications. The complete calculation is given as follows:

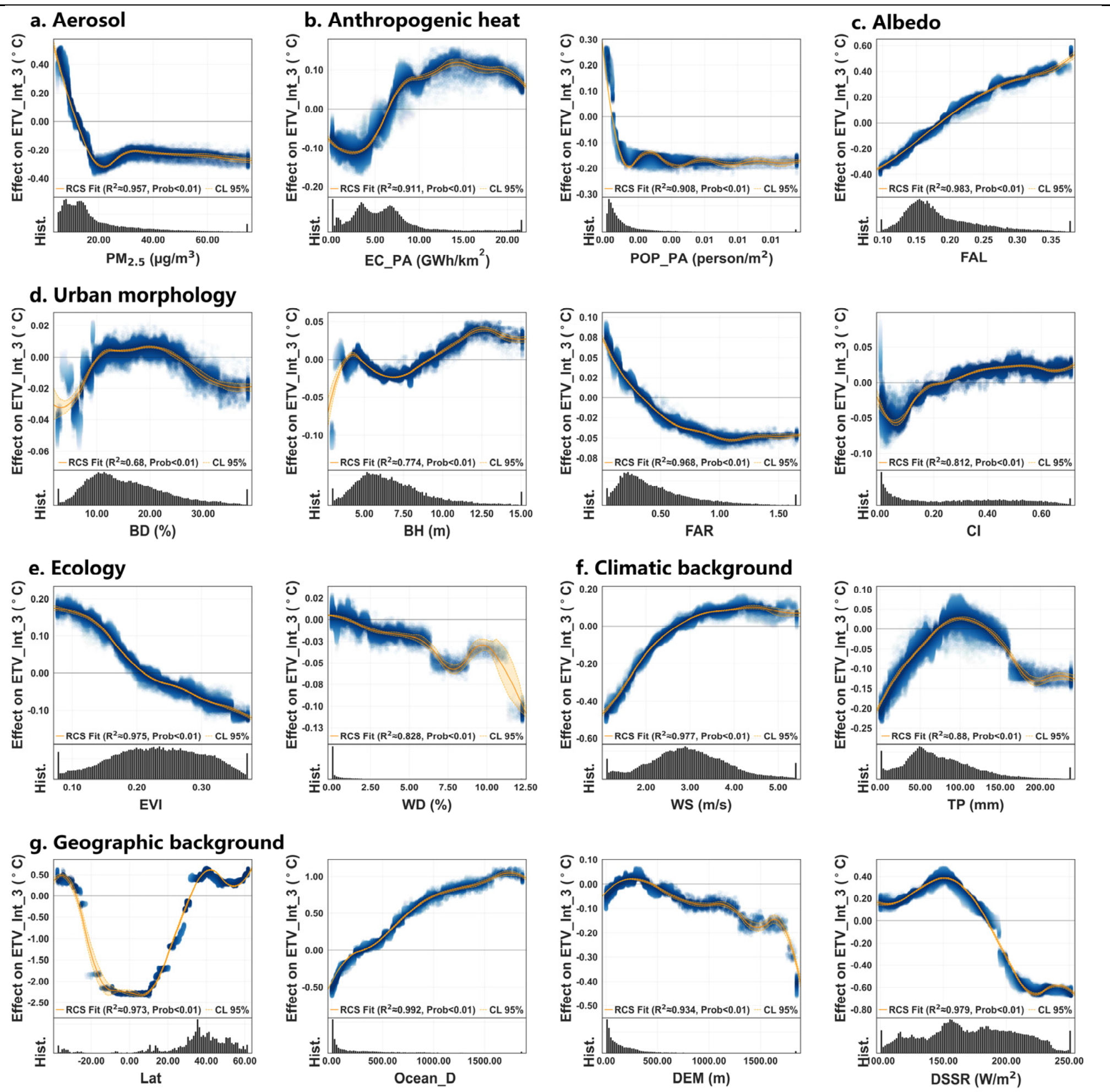

Supplementary Fig. 4 **The results on ETV_Int. using a three-day rolling window.** Local effect of each feature included in ensemble learning models on ETV_Int. using a three day rolling window (n=157,830).

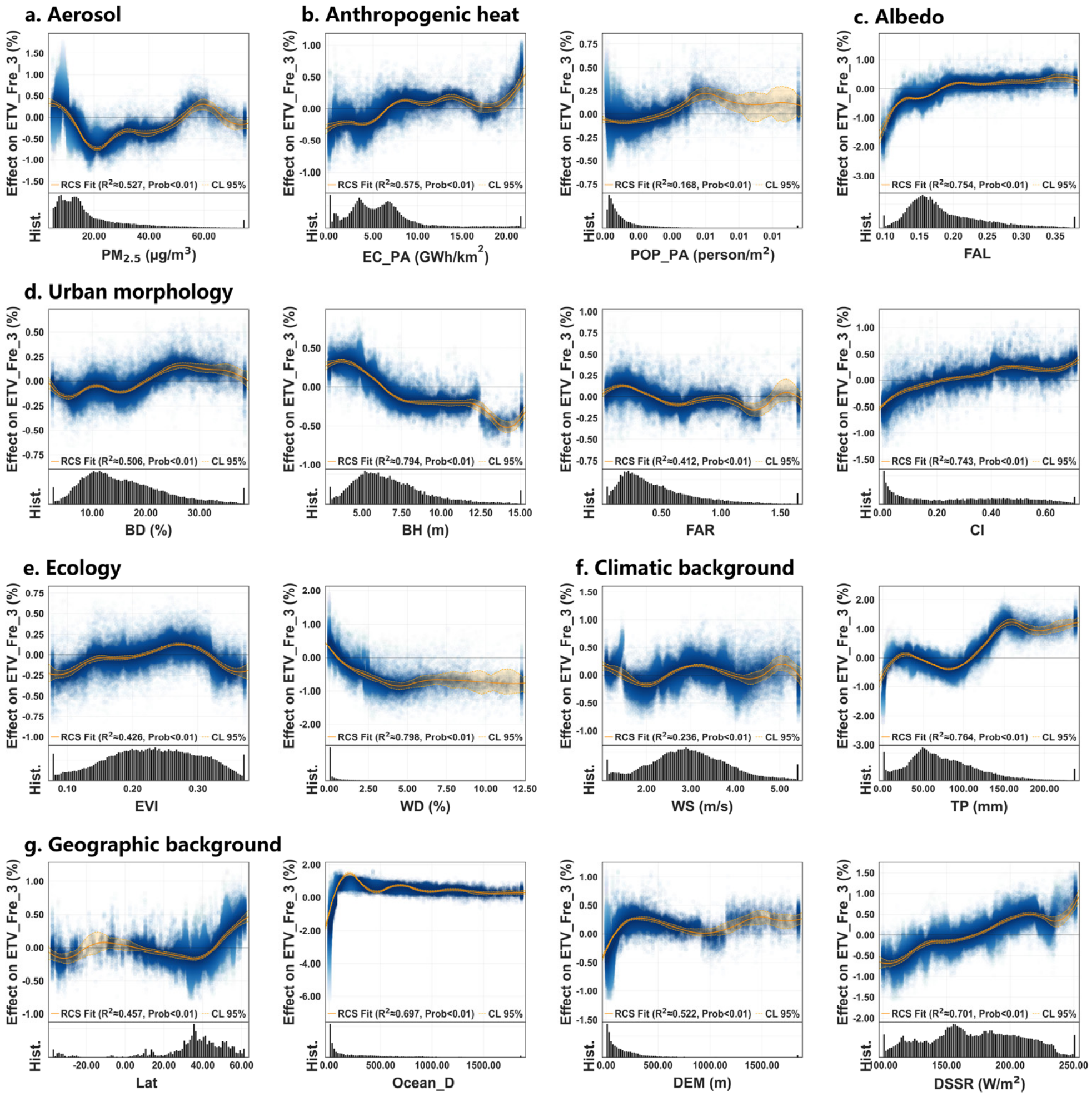

Supplementary Fig. 5 **The results on ETV_Fre. using a three day rolling window.** Local effect of each feature included in ensemble learning models on ETV_Fre. using a three day rolling window (n=157,830).

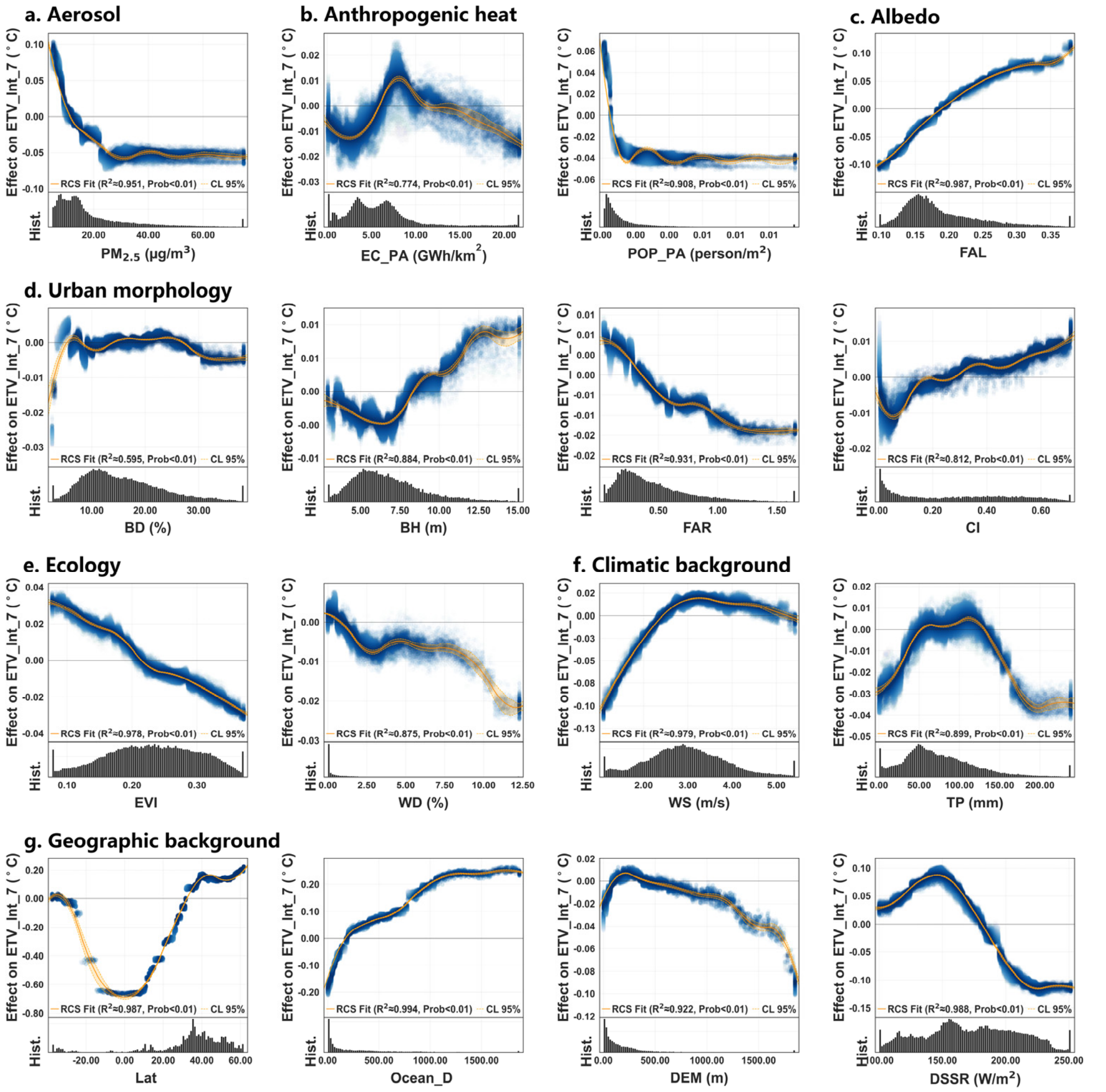

Supplementary Fig. 6 **The results on ETV_Int. using a seven day rolling window.** Local effect of each feature included in ensemble learning models on ETV_Int. using a seven day rolling window (n=157,830).

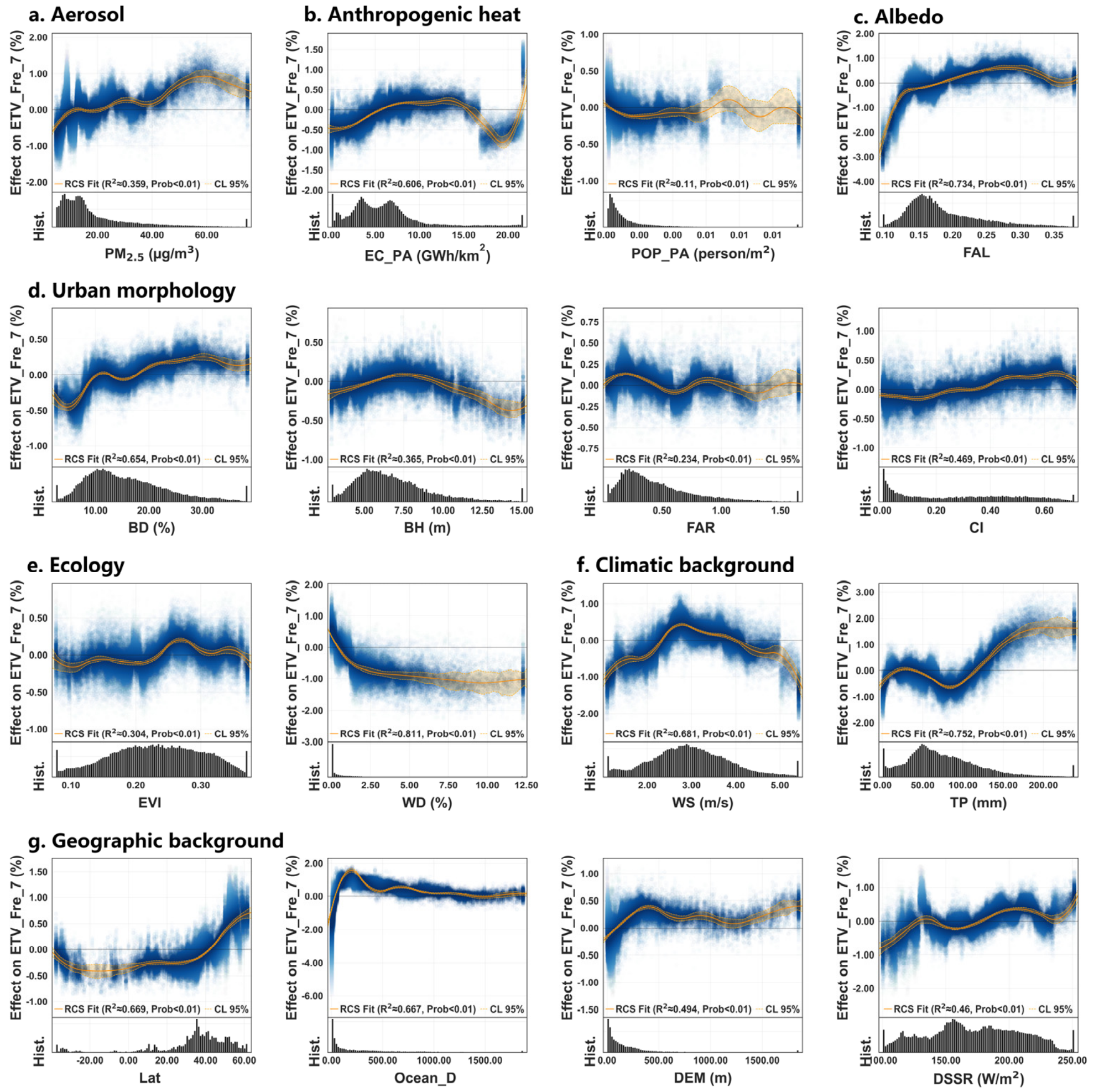

Supplementary Fig. 7 **The results on ETV_Fre. using a seven day rolling window.** Local effect of each feature included in ensemble learning models on ETV_Fre. using a seven day rolling window (n=157,830).

Responses to Reviewer #2's comments:

This manuscript presents a comprehensive global analysis of extreme temperature variability (ETV) across 10,522 cities from 1950 to 2020 using an interpretable machine-learning framework. By shifting the focus from mean temperature change to day-to-day variability and decomposing ETV into intensity and frequency components, the study makes a valuable and timely contribution to urban climate science. The identification of aerosols as a dominant urban factor and the finding that high-HDI cities experience intensifying ETV are particularly novel and policy-relevant. Overall, the data integration effort is impressive, the structure is clear, and the paper is suitable for publication after minor revision.

Response:

Thank you for your concurrence on the significance of our study on hydrological risks and for acknowledging the timeliness of this work. We appreciate your feedback and suggestions on improving the presentation of our results. Following your recommendation, we have carefully revised the manuscript to further improve methodological transparency, figure readability, and the discussion of limitations and policy implications. We believe these revisions have strengthened the manuscript and improved its overall clarity and rigor.

Thank you again for your constructive assessment and for supporting the publication of this work after minor revision.

Comment 1:

The definitions and relationships among “annual mean ETV,” “event intensity,” and “event frequency” should be summarized in a unified framework (e.g., at the beginning of the Methods or Results) to avoid confusion between cumulative and single-event metrics.

Response:

We thank you for this helpful suggestion. We agree that the distinctions among “annual mean ETV,” “event frequency,” and “event intensity” were not sufficiently explicit in the original manuscript. In the revised manuscript, we have added a brief, unified conceptual framework at the beginning of the Methods section. Annual cumulative ETV represents the total yearly variability load, event frequency captures how often variability events occur, and event intensity characterizes how large those events are when

they occur.

**Corresponding modified content:**
**METHODS**<br>**Extreme Temperature Variability**<br>……<br>We developed a consistent metric for monitoring and assessing ETV by integrating the TX90 extreme index, recommended by the Expert Team on Climate Change Detection and Indices (ETCCDI: https://www.climdex.org/learn/indices/), with the day-to-day temperature variability index $\Delta T_{c,d,y,W}$. For clarity, we distinguish three related but conceptually different annual metrics derived from daily ETV events. We first calculate daily weather-scale temperature variability and define an ETV event day as a day when this daily value exceeds a calendar-day-specific 90th-percentile threshold. Based on these event days, we derive annual cumulative ETV, defined as the total exceedance load accumulated over a year; event frequency, defined as the number of event days per year; and event intensity, defined as the mean exceedance magnitude of event days. Annual cumulative ETV, therefore, reflects the combined outcome of how often ETV events occur and how large they are, whereas event frequency and event intensity isolate these two components. The complete calculation formula is defined as follows:

Comment 2:

The numerous urbanization-related variables would benefit from a consolidated table summarizing their definitions, units, data sources, and theoretical motivations. The interpretation of blue – green infrastructure should also be clarified, as the current indicators capture average coverage rather than spatial connectivity, while the text sometimes implies the latter.

Response:

Thank you for your valuable feedback. In the revised manuscript, we have added a summary table that lists each urban variable, its definition, unit, and theoretical motivation. We believe this addition improves the transparency of the variable design and makes the modeling framework easier to interpret. We also appreciate your point regarding the interpretation of BGI. You are correct that the indicators, mean annual vegetation index and water density within city boundaries, capture average vegetation and water coverage rather than spatial configuration or connectivity. In the original text, some wording could indeed be read as implying connected or contiguous blue–green networks more directly than the data support.

**Corresponding modified content:**

METHODS

……

Urbanization-related Indicators

……

Regarding urban ecological factors, we used the Enhanced Vegetation Index (EVI) derived from the MODIS vegetation index product MOD13A1 (https://lpdaac.usgs.gov/products/mod13a1v061/), which has a stable monthly update frequency and a spatial resolution of 1 km. Monthly EVI values were aggregated annually to compute the mean annual EVI within each city boundary, providing a comprehensive representation of urban vegetation levels throughout the study period. For blue infrastructure, we utilized the Global WaterPack dataset provided by the German Aerospace Center. This dataset tracks the global distribution and daily dynamics of water bodies with a high spatial resolution of 250 meters[44]. Based on this data, we calculated the annual average water density (WD) within each city boundary to assess the dynamic features of urban blue infrastructure.

SUPPLEMENTARY INFORMATION

……

Supplementary Table. 1

Category	Variable	Unit	Theoretical motivation
Aerosol	$PM_{2.5}$	$\mu g/m^3$	Modifies radiation balance and boundary-layer stability, thereby shaping thermal variability.
Anthropogenic heat	EC_PA	GWh/km^2	Proxies energy use and associated sensible heat release within cities.
	POP_PA	$person/m^2$	Captures human activity intensity and its associated heat and emission load.
Urban morphology	BD	%	Alters impervious cover, shading, ventilation resistance, and heat storage.
	BH	m	Regulates roughness, canyon trapping, and turbulent heat exchange.
	FAR	dimensionless	Reflects built volume intensity and the effective thermal capacity of urban form.
	CI	dimensionless	Captures urban compactness, with implications for ventilation and heat dispersion.
Ecology	EVI	dimensionless	Represents vegetation cover and its evaporative and shading effects.
	WD	%	Represents water coverage and its thermal buffering through high heat capacity.
Albedo	FAL	dimensionless	Controls shortwave reflection and surface heat absorption.
Climatic background	WS	m/s	Governs ventilation strength and near-surface heat transport.
	TP	mm	Reflects moisture conditions that modify evaporative cooling and thermal response.
	DSSR	W/m^2	Represents incoming solar energy available for daytime surface heating.
Geographic background	Lat	dimensionless	Captures large-scale gradients in seasonality, radiation, and circulation.
	Ocean_D	km	Represents the strength of marine

			moderation on day-to-day temperature swings.
	DEM	m	Captures topographic control on local thermal conditions and airflow.

Comment 3:

The subsection title “Urbanization mitigates ETV” appears stronger than what the results support, given the documented divergence among cities and the intensification in high-HDI regions, and may warrant rephrasing.

Response:

We thank you for this helpful comment. We agree that the original subsection title, “Urbanization mitigates ETV,” was overly strong relative to the results. In the revised manuscript, we have therefore replaced this subsection title with a more neutral and accurate formulation: “Urban factors associated with ETV.” This revised title better reflects the scope of the analysis, namely that we assess how multiple urban characteristics are associated with ETV after controlling for climatic and geographic background, rather than claiming that urbanization mitigates ETV.

Corresponding modified content:

RESULTS

……

Urban factors associated with ETV

The overall magnitude of urban ETV has decreased, yet the gaps between cities have widened. Climate and geography account for much of the baseline spatial pattern, but they cannot explain this pattern of "mean convergence with stretched extremes." Urbanization processes are driving the divergence16. The pace of surface sealing, the strength of sensible heat emissions, the extent of blue－green infrastructure, and block-scale morphology that governs ventilation and heat storage are reshaping local ETV trends[20-22]. To assess whether city-level urban characteristics are associated with this divergence, we developed an interpretable machine-learning framework for the 2005－2019 period using 10,522 cities with built-up areas exceeding 10 km^2, the sample for which annual urban descriptors are available. After controlling for macro-climatic and geographic background, five urban factors, including aerosol concentration, anthropogenic heat, urban morphology, blue－green infrastructure, and surface albedo, explain 30.83% of the spatiotemporal variation in ETV intensity and 48.85% in ETV frequency. The models achieve R^2 values of 0.97 for intensity and 0.84 for frequency. Crucially, the association patterns of these factors differ significantly between intensity and frequency, indicating distinct levers for risk management. The association patterns of climatic and geographic background, sensitivity analyses based on GAMs, and 3/7-day rolling windows are

provided in Supplementary Figs. 1, 2, 3, 4, 5, 6, and 7.

Comment 4:

Regarding presentation, Figures 1, 2, and 4 are overly dense. Reducing the number of subpanels, clearly explaining all abbreviations, standardizing color bars, and moving secondary results to the Supplementary Information would improve readability.

Response:

We thank you for this helpful suggestion. We agree that the original presentation of Figs. 1, 2, and 4 were visually too dense, and the large number of subpanels reduced readability. In the revised manuscript, we have simplified the figure presentation in several ways. For Fig. 1 and 2, we redesigned the layout and revised the regional heatmap presentation to improve visual clarity. For Fig. 4, we restructured the spatial maps by summarizing the results at the second-level administrative unit, aligning the coordinate framework across panels, and enlarging the maps to make major spatial gradients more clearly identifiable. We hope these changes substantially improve the readability and visual consistency of the manuscript.

Corresponding modified content:

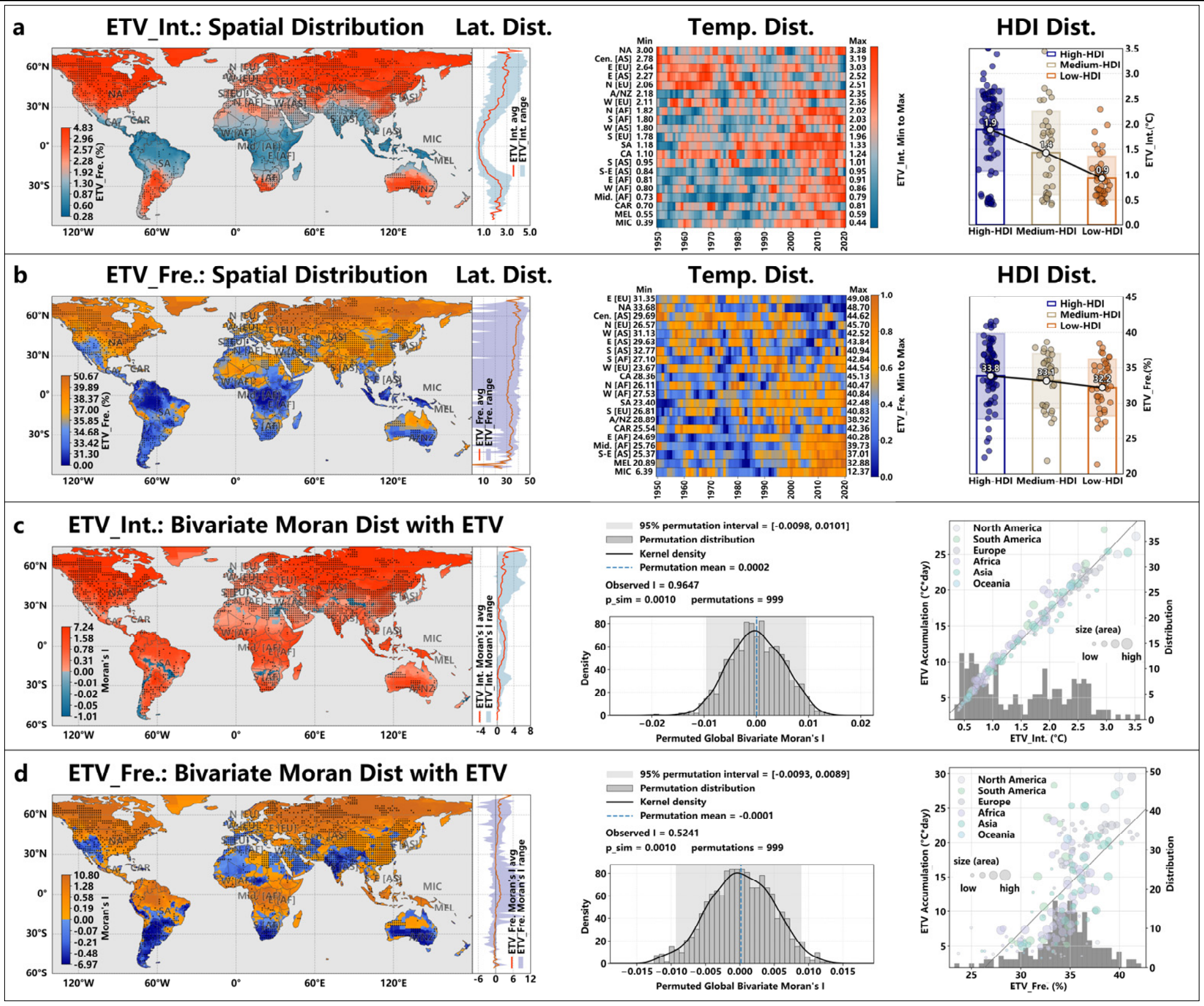

Fig. 1. **Spatio-temporal distribution of ETV.**

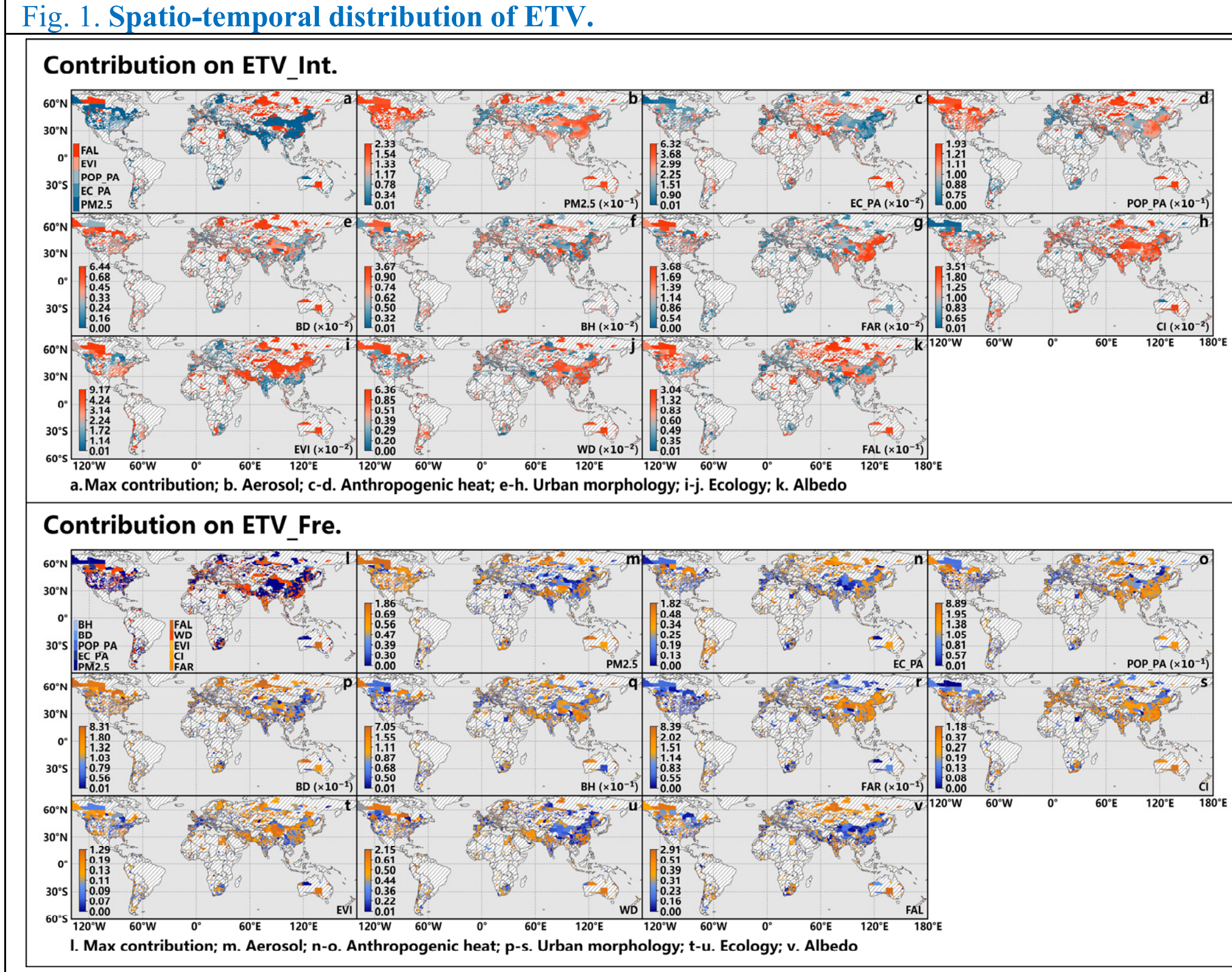

Fig. 4. **The sensitivity of urban factors on ETV.**

Comment 5:

For the CMIP6 comparison in Figure 1, clarifying the time period and/or including future projections or multiple models would strengthen its relevance.

Response:

We thank you for this valuable suggestion. In the revised manuscript, we now explicitly clarify the time period used for the EC-Earth3 comparison in the figure caption, so that readers can more readily interpret its relationship to the ERA5-Land analysis.

We agree that extending the analysis to multiple CMIP6 models and future scenarios would further strengthen the broader relevance of this comparison. To address this point, we have added explicit discussion in the limitations section, noting that future work should evaluate the robustness of these patterns using multi-model ensembles and projected climate scenarios, particularly to assess how the divergence of urban ETV may evolve under continued warming and urbanization.

Corresponding modified content:

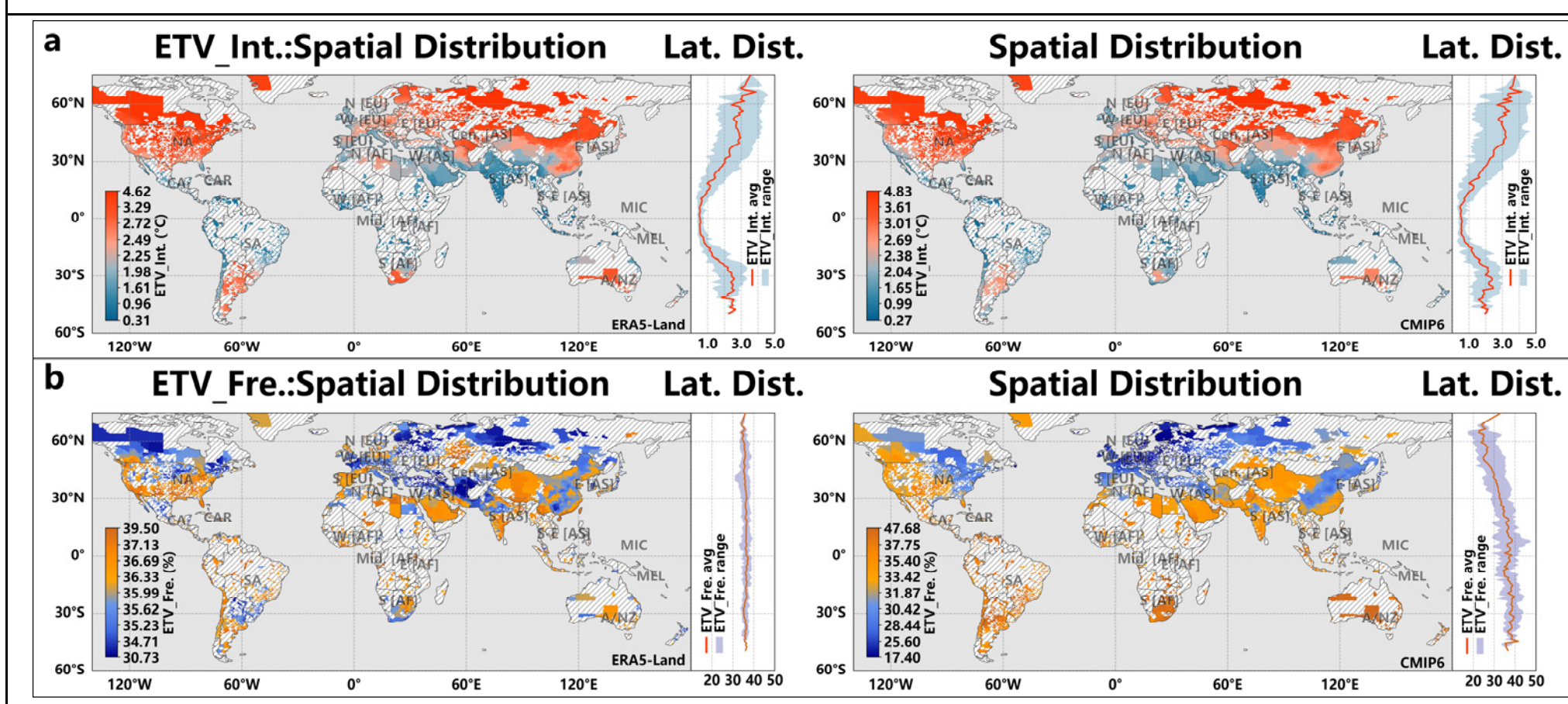

Supplementary Fig. 8 **Spatio-temporal distribution of ETV between ERA5-Land and CMIP6.** We used daily near-surface air temperature from the EC-Earth3 climate model, which participated in CMIP6, from 2005 to 2019.

DISCUSSION

……

Finally, we acknowledge several limitations and outline the next steps. Our analysis is designed for the city scale. Because ETV is derived from gridded daily near-surface air temperature, the dataset cannot fully resolve neighborhood- or canopy-scale thermal heterogeneity. Our conclusions should therefore be interpreted as city-level patterns rather than direct evidence of street-scale urban heat processes. Second, the next-step research should employ counterfactual designs or instrumental-

variable strategies to disentangle the roles of emissions, morphology, and meteorology, clarify causal effects, and strengthen threshold estimates for the urban-process variable. Finally, future research should include future projections from CMIP6 models to assess trends in ETV across different scenarios. Despite these limitations, our harmonized analysis of 10,522 cities shows that urbanization is reshaping the intensity and frequency structure of temperature risk. By disentangling how urbanization processes interact with the climatic background, we outline a practical path to steer cities out of the high-frequency, large-amplitude tail of risk and back into a safer operating space.

<u>Comment 6:</u>

A short paragraph summarizing the main take-home message and its concrete implications for urban climate governance would help bridge the technical analysis and policy discussion.

<u>Response:</u>

We thank you for this helpful suggestion. In response to your feedback, we have added a concise take-home paragraph to the Discussion to summarize the study's main message and clarify its practical relevance. We also use this paragraph to clarify the concrete policy relevance of aerosol control, ecological coverage, and morphology-sensitive design as management-relevant directions that require local validation. We hope this addition improves the bridge between the analytical results and the policy discussion.

Corresponding modified content:

DISCUSSION

……

More broadly, the value of this analysis lies less in prescribing a universal intervention package than in informing policy sequencing and regional prioritization. In South Asia and East Asia, where heavy pollution is associated with a greater risk of fluctuations, air-quality control should be paired with albedo management and ventilation-sensitive design. In dense urban corridors of the Middle East and the United States, morphology-sensitive retrofits and ventilation corridors may be more effective than green expansion alone. By contrast, cities in Western Europe, Eastern Europe, and the eastern United States may gain more from protecting and connecting existing blue–green networks. Because the present study is observational and retrospective, these directions should be interpreted as management-relevant rather than as causal or scenario-tested prescriptions.

<u>Comment 7:</u>

The code is generally well structured and the results appear reproducible in principle. However, in-line

comments and documentation are limited. Adding more comments and a clearer README with installation and usage instructions would improve transparency and usability for the community.

<u>Response:</u>

We thank you for this helpful suggestion. In response, we have also added a dedicated README file with detailed installation and usage instructions. The README now describes the required Python environment and packages, the repository structure, the input data format, the main steps for running the code, and the expected outputs. In addition, we provide a sample dataset to illustrate the required data structure and help users more easily reproduce the workflow.

Responses to Reviewer #3's comments:

This manuscript presents an interesting study on extreme temperature variability (ETV) across global cities, offering a novel perspective on urban climate risk beyond mean temperature and conventional extremes. The large-scale dataset and the integration of urban factors with climate background provide valuable insights into the spatial heterogeneity and potential drivers of temperature variability. However, the following points still need further consideration, which will help further clarify the choice of methods and enhance the reliability and interpretability of the results.

Response:

We sincerely thank you for your careful reading of our manuscript and for your constructive overall assessment. We greatly appreciate your recognition that the study offers a novel perspective on urban climate risk by focusing on ETV, and that the large-scale dataset and the integration of urban factors with climatic background provide valuable insights into the spatial heterogeneity and potential drivers of urban temperature variability.

We also appreciate your thoughtful comments on the remaining issues that require further clarification. Following your suggestions, we have carefully revised the manuscript to improve methodological transparency, strengthen the interpretation of the results, and better define the scope of our conclusions. These comments have helped us improve the rigor, clarity, and readability of the manuscript. Below, we respond to each of your comments in detail.

Comment 1:

This study proposes an interesting metric of Extreme Temperature Variability (ETV), which provides a useful perspective beyond mean temperature and traditional extremes. However, the construction of ETV, particularly the use of a 5-day rolling window and the 90th percentile threshold, would benefit from further justification. It is not entirely clear whether these parameter choices are theoretically grounded or empirically driven.

Response:

We thank you for this important comment. We agree that the rationale for the ETV construction, particularly the use of the 5-day rolling window and the 90th-percentile threshold, should be stated more

explicitly. Specifically, the 5-day rolling window was used to represent weather-scale thermal fluctuations associated with synoptic variability while reducing sensitivity to isolated single-day noise. Our intention was to capture short-term temperature swings relevant to urban exposure and infrastructure stress, without overreacting to transient daily anomalies. To assess the robustness of this choice, we additionally repeated the analysis using 3-day and 7-day rolling windows. The main spatial patterns and core conclusions remained qualitatively consistent across these alternative specifications.

The 90th percentile threshold was selected to identify the upper tail of local temperature-variability events in a manner comparable across diverse climatic regimes. By using a calendar-day-specific percentile threshold, the method accounts for seasonality and local climatology, following the general logic of percentile-based extreme indices such as those recommended by ETCCDI, while adapting that framework to day-to-day variability.

Accordingly, we have revised the Methods section to clarify the conceptual basis of both parameters and added the 3-day and 7-day window results as robustness checks. We hope this revision better explains the methodological choices and strengthens the interpretability of the ETV framework.

Corresponding modified content:

METHODS

Extreme Temperature Variability

In this study, the city is treated as the spatial unit of analysis with second-level administrative units from the GADM global database, and annual indicators from 1950 to 2020 are constructed to characterize city-level ETV. To quantify day-to-day temperature variability, we calculated daily temperature differences and smoothed these differences using a five-day rolling window. This window was chosen to represent weather-scale thermal fluctuations associated with synoptic variability while reducing sensitivity to isolated single-day noise. To assess the robustness of this choice, we additionally repeated the analysis using 3-day and 7-day rolling windows, and the main results remained qualitatively consistent across specifications. The complete calculation is given as follows:

……

We developed a consistent metric for monitoring and assessing ETV by integrating the TX90 extreme index, recommended by the Expert Team on Climate Change Detection and Indices (ETCCDI: https://www.climdex.org/learn/indices/), with the day-to-day temperature variability index $\Delta T_{c,d,y,W}$. For clarity, we distinguish three related but conceptually different annual metrics derived from daily ETV events. We first calculate daily weather-scale temperature variability and define an ETV event day as a day when this daily value exceeds a calendar-day-specific 90th-percentile threshold. Based on these event days, we derive annual cumulative ETV, defined as the total exceedance load accumulated over a year; event frequency, defined as the number of event days per

year; and event intensity, defined as the mean exceedance magnitude of event days. Annual cumulative ETV, therefore, reflects the combined outcome of how often ETV events occur and how large they are, whereas event frequency and event intensity isolate these two components. The complete calculation formula is defined as follows:

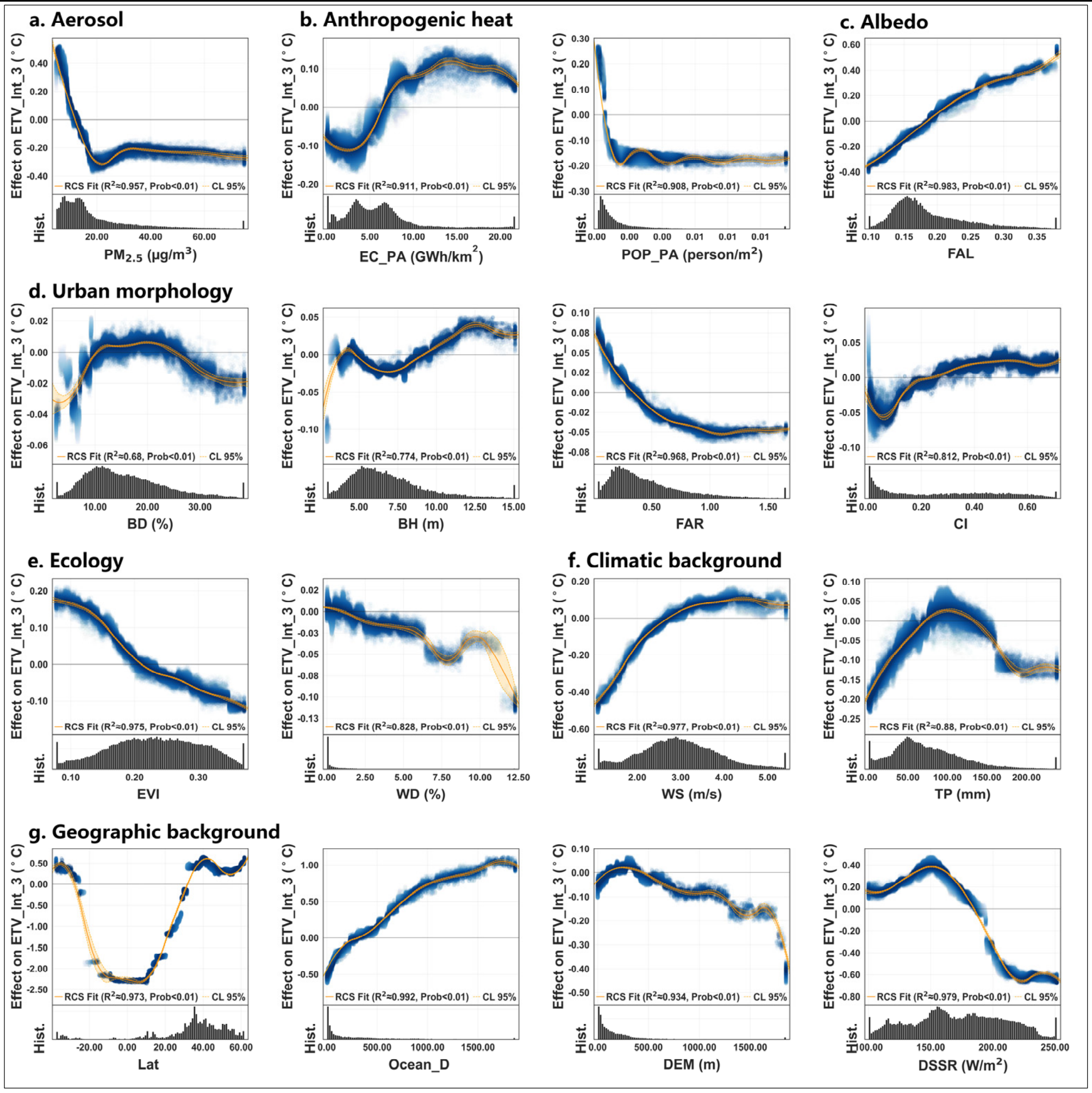

Supplementary Fig. 4 **The results on ETV_Int. using a three-day rolling window.** Local effect of each feature included in ensemble learning models on ETV_Int. using a three day rolling window (n=157,830).

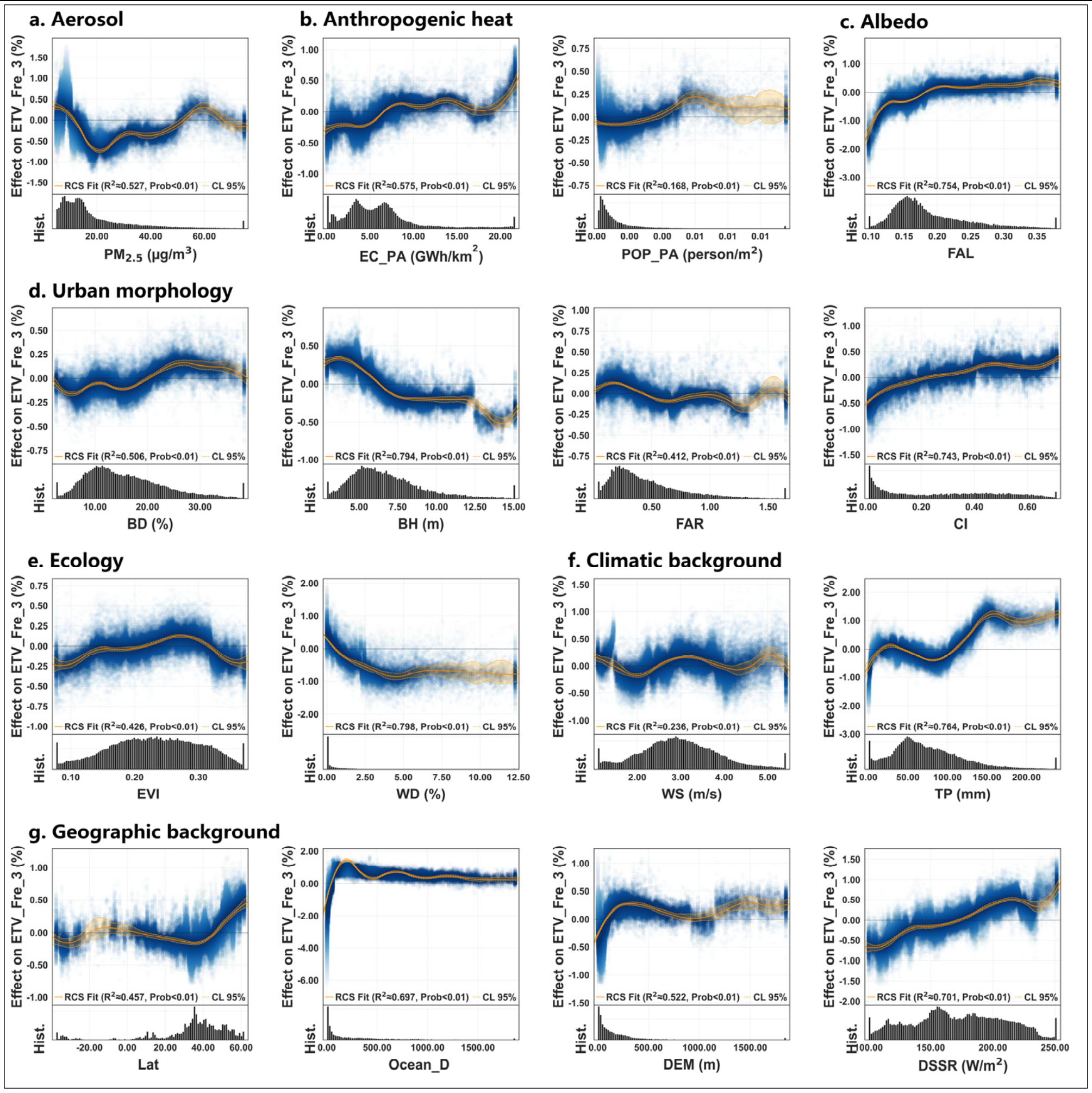

Supplementary Fig. 5 **The results on ETV_Fre. using a three day rolling window.** Local effect of each feature included in ensemble learning models on ETV_Fre. using a three day rolling window (n=157,830).

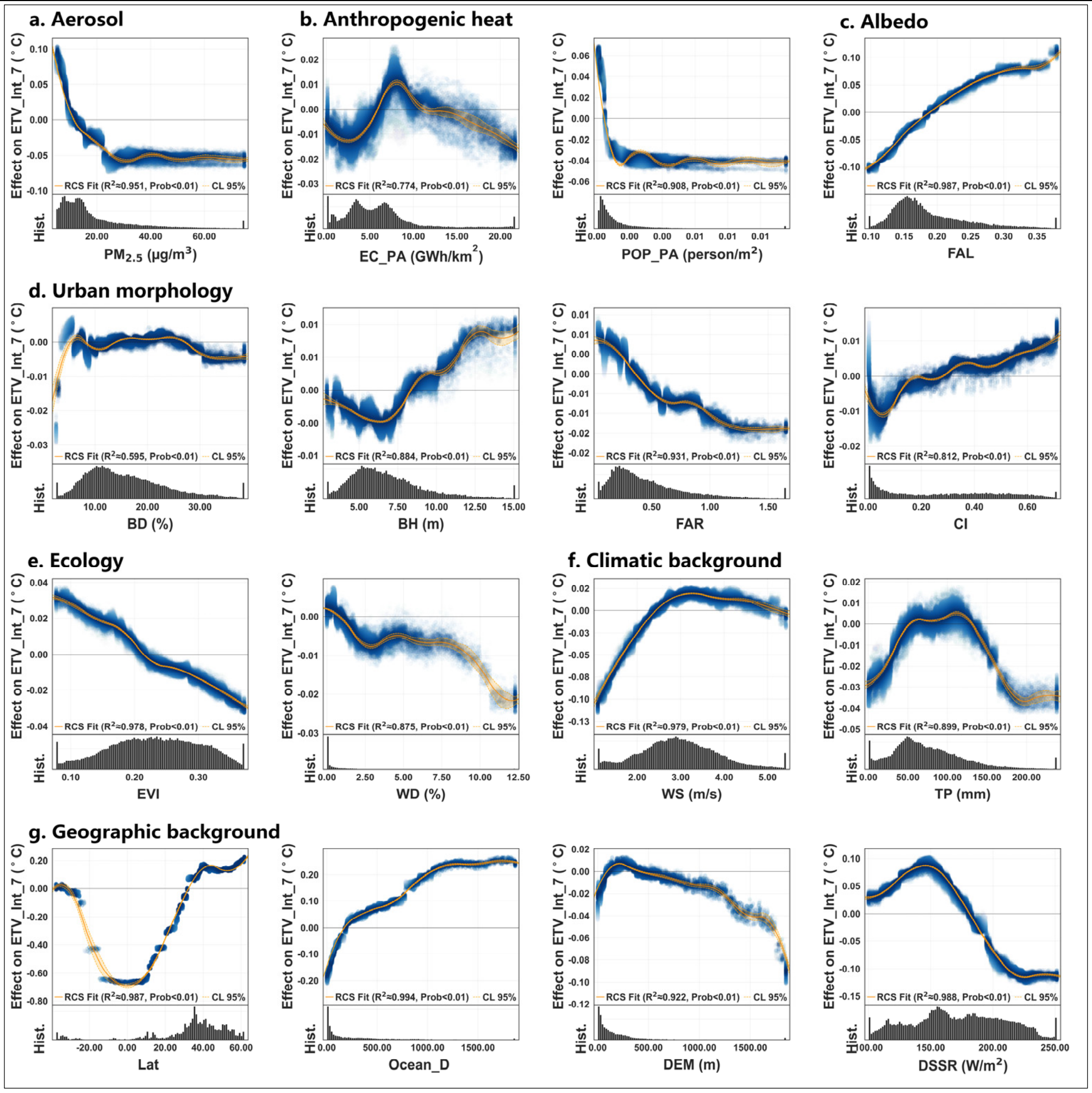

Supplementary Fig. 6 **The results on ETV_Int. using a seven day rolling window.** Local effect of each feature included in ensemble learning models on ETV_Int. using a seven day rolling window (n=157,830).

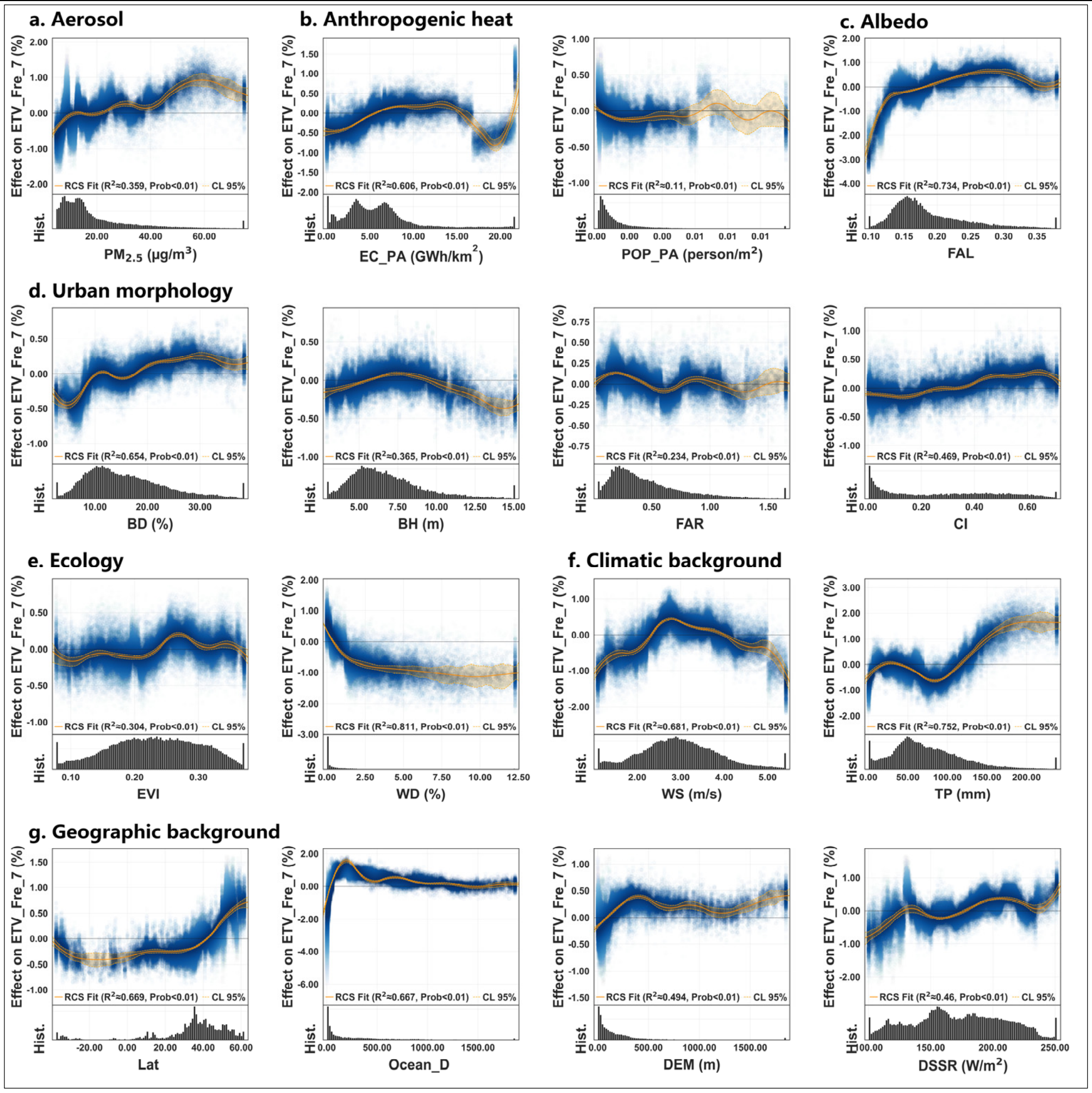

Supplementary Fig. 7 **The results on ETV_Fre. using a seven day rolling window.** Local effect of each feature included in ensemble learning models on ETV_Fre. using a seven day rolling window (n=157,830).

Comment 2:

The decomposition of ETV into intensity and frequency is helpful for understanding temperature variability, and the finding that intensity dominates is insightful. However, it would be useful to briefly clarify the rationale for this decomposition and its practical or physical significance. It would also be helpful to indicate whether this framework is based on prior studies or newly proposed in this work.

Response:

We thank you for this helpful comment. We agree that the practical and physical significance of decomposing ETV into event frequency and single-event intensity should be stated more explicitly. We use bivariate Moran's I to identify which component more closely explains the spatial pattern of annual

cumulative ETV. This analysis shows that cumulative ETV is spatially much more concordant with event intensity (Moran's I 0.96) than with event frequency (Moran's I 0.53), indicating that recent divergence across cities is primarily intensity driven.

The practical significance is equally important. Many moderate events and a few severe events may produce similar annual cumulative ETV, but they imply very different impacts and management priorities. Intensity-dominant risk means that a small number of large swings can generate disproportionate burdens for health, energy demand, infrastructure performance, and emergency response. It also implies that adaptation should focus not only on reducing the number of event days, but also on limiting event amplitude and shortening post-event recovery. In this sense, identifying intensity as the dominant component helps explain why annual ETV is a meaningful risk metric and why event amplitude is a relevant target for urban climate governance.

Corresponding modified content:

RESULTS

ETV's spatio-temporal change trends

……

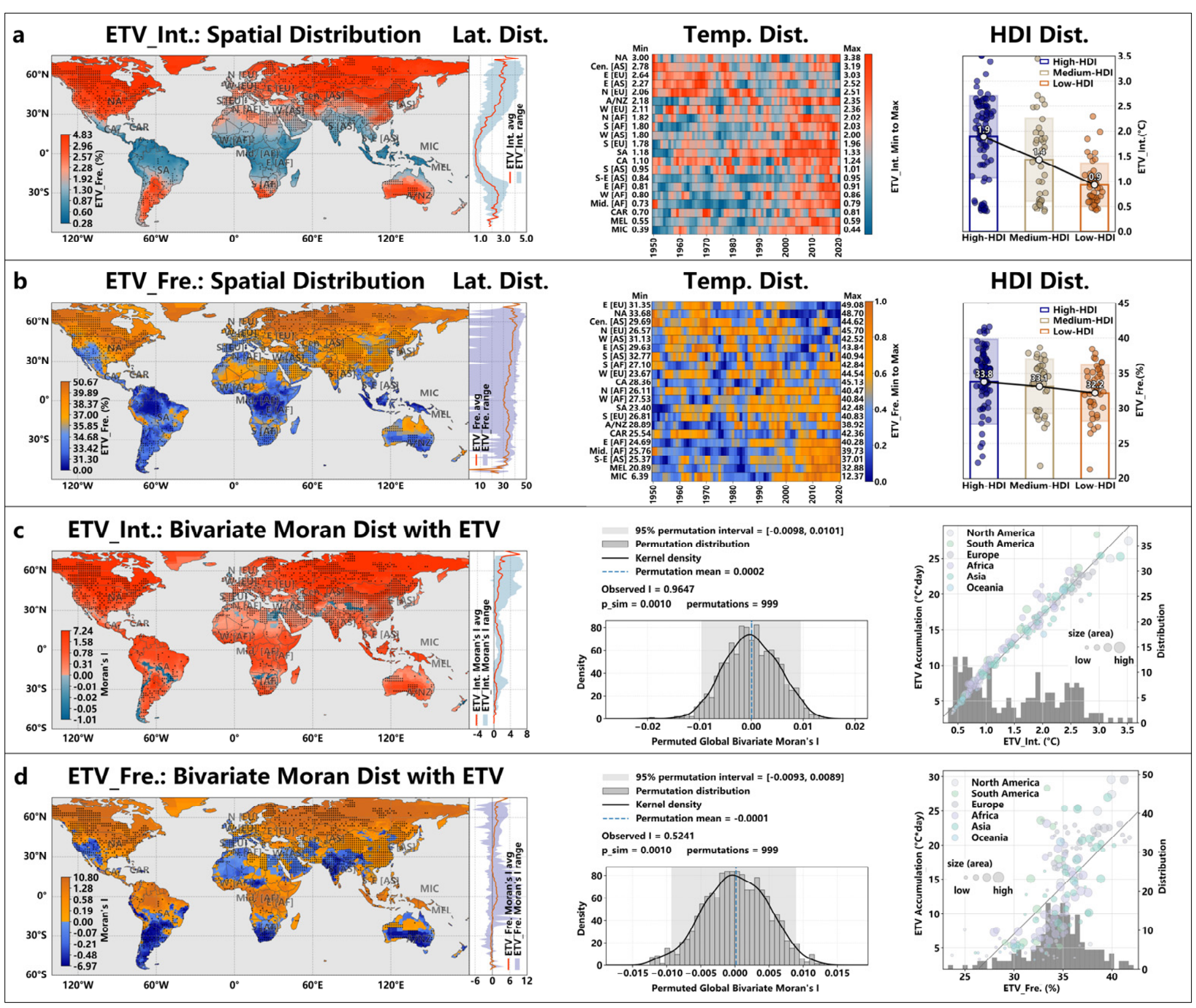

Fig. 1. **Spatio-temporal distribution of ETV.** The subfigures a and b are based on ERA5-Land. The

average geographical distribution of ETV from 1950 to 2020, with points representing built-up areas, a heatmap by geographic subregions, and a distribution by human development index (HDI) groups. The subfigures c and d show the Bivariate Moran' s I between ETV_Int. and ETV, as well as ETV_Fre. and ETV. The positive indicates that locations with high (low) values in ETV_Int. or ETV_Fre. tend to be surrounded by high (low) values in ETV as well.

……

We decompose the annual ETV (1950–2020) into two metrics, event frequency (the number of variability days per year) and event intensity (the magnitude of each variability). To identify which component dominates the annual cumulative ETV pattern, we compare its spatial correspondence with the frequency and intensity fields using bivariate Moran's I. The result shows that the spatial pattern of cumulative ETV mirrors the intensity field (Moran's I 0.96) and differs markedly from the frequency field (Moran's I 0.53). In the high-latitude belt, the number of variability days changes little, but larger per-event swings push annual totals to global maxima. Many low-latitude coastal and humid green-belt cities exhibit modest increases in variability days, yet maintain low cumulative loads because evapotranspiration and oceanic heat capacity mitigate the amplitudes[19]. This means that similar annual ETV loads can arise from very different event structures, but in intensity-dominant regimes, risk is concentrated in a relatively small number of high-amplitude events. Such events can impose disproportionate burdens on health, energy systems, and infrastructure. Focusing on event amplitude, therefore, provides a more relevant basis for early warning, design thresholds, and risk management than event counts alone. Reducing the upper tail of event amplitude may be especially important in high-latitude inland cities, where cumulative ETV is most strongly concentrated.

<u>Comment 3:</u>

The selection of urban indicators in this study is comprehensive and represents a clear strength of the paper. However, the rationale behind the selection of these variables is not yet entirely clear. It is recommended that the authors provide further clarification on the logic behind the choice of these indicators. For example, whether it is based on a theoretical framework, data availability, or empirical support from existing literature. Such explanations could be included in the Methods or Discussion sections to enhance the transparency of the analysis and the soundness of the interpretation of the results.

<u>Response:</u>

We thank you for this helpful comment. We agree that the rationale for selecting the urban indicators should be stated more explicitly. In the revised manuscript, we now clarify that the indicator selection was guided by three considerations rather than by data availability alone. First, it was anchored in an urban surface energy-balance framework, in which cities modify thermal variability through five main

pathways: radiative forcing, anthropogenic heat input, aerodynamic and heat-storage effects of urban form, hydrological buffering by vegetation and water, and surface reflectance. Second, we retained only variables with globally consistent coverage and annual observations for 2005–2019, the period for which urban covariates were comparably available across cities. Third, the selected indicators are supported by prior empirical literature linking these urban processes to thermal conditions and heat-related risk. We have added this rationale to the Methods section and provided a consolidated table summarizing each variable, its unit, and theoretical motivation. We hope this revision improves the transparency of the analytical design and the interpretation of the results.

Corresponding modified content:

METHODS

……

Urbanization-related Indicators

To systematically characterize urbanization in relation to ETV, the selection of urban indicators was guided by an urban surface energy-balance framework, in which cities influence the thermal environment through five major pathways: radiative forcing, anthropogenic heat input, aerodynamic and heat-storage effects of urban form, hydrological buffering by vegetation and water, and surface reflectance[14]. Therefore, we compiled five categories of raster-based indicators with global coverage and annual observations for 2005-2019, the period for which urban covariates are consistently available. For each year, these variables were aggregated to city-level means within each city's contemporaneous built-up footprint and then used in subsequent urban-factor analyses. City boundaries were derived from annually updated built-up extents based on multi-source remote-sensing products[40]. Because our analysis targets city-level, weather-scale ETV rather than neighborhood-scale thermal heterogeneity, and to reduce the mismatch between dynamic urban polygons and the underlying meteorological grid, we retained only cities with a built-up area of at least 10 km^2 during the urban-factor analysis period. This filter excludes very small settlements and improves the global comparability of both urban extent and urban descriptors. A total of 10,522 cities met these criteria and were included in the analysis. Note that ETV itself was calculated for 1950-2020, whereas analyses linking ETV to urbanization-related factors were restricted to 2005-2019 owing to data availability.

SUPPLEMENTARY INFORMATION

……

Supplementary Table. 1

Category	Variable	Unit	Theoretical motivation
Aerosol	$PM_{2.5}$	$\mu g/m^3$	Modifies radiation balance and boundary-layer stability, thereby shaping thermal variability.
Anthropogenic heat	EC_PA	GWh/km^2	Proxies energy use and associated sensible heat release within cities.

	POP_PA	person/m^2	Captures human activity intensity and its associated heat and emission load.
Urban morphology	BD	%	Alters impervious cover, shading, ventilation resistance, and heat storage.
	BH	m	Regulates roughness, canyon trapping, and turbulent heat exchange.
	FAR	dimensionless	Reflects built volume intensity and the effective thermal capacity of urban form.
	CI	dimensionless	Captures urban compactness, with implications for ventilation and heat dispersion.
Ecology	EVI	dimensionless	Represents vegetation cover and its evaporative and shading effects.
	WD	%	Represents water coverage and its thermal buffering through high heat capacity.
Albedo	FAL	dimensionless	Controls shortwave reflection and surface heat absorption.
Climatic background	WS	m/s	Governs ventilation strength and near-surface heat transport.
	TP	mm	Reflects moisture conditions that modify evaporative cooling and thermal response.
	DSSR	W/m^2	Represents incoming solar energy available for daytime surface heating.
Geographic background	Lat	dimensionless	Captures large-scale gradients in seasonality, radiation, and circulation.
	Ocean_D	km	Represents the strength of marine moderation on day-to-day temperature swings.
	DEM	m	Captures topographic control on local thermal conditions and airflow.

Comment 4:

The use of machine learning is appropriate for capturing nonlinear relationships and provides useful interpretability. However, it would be helpful for the authors to further clarify why this approach was chosen over more traditional statistical or econometric models. In particular, what are the specific advantages of this framework in the context of this study? Furthermore, it should be noted that SHAP essentially reflects the association between variables and outcomes, rather than a causal relationship; this point could be made clearer in the interpretation of the results.

Response:

We thank you for this important comment. Our choice was driven by the structure of the research question and the data. Specifically, the relationships between ETV and urban characteristics are expected to be nonlinear, threshold-dependent, and potentially interactive, and to vary across diverse climatic and

developmental contexts. In this setting, conventional linear or econometric models are useful for estimating pre-specified marginal relationships, but they require stronger functional-form assumptions and become difficult to parameterize when multiple nonlinearities and higher-order interactions are expected.

To address this, we now clarify in the revised manuscript that our analytical strategy combines LightGBM and GAM, rather than treating machine learning as a substitute for statistical modelling. LightGBM is used to flexibly capture nonlinear responses and higher-order interactions without imposing a priori parametric forms, and to identify the relative importance and local response patterns of urban variables. GAM is used as a complementary semi-parametric framework to assess the statistical significance, functional direction, and robustness of the major associations identified by LightGBM. In this sense, LightGBM serves primarily as a discovery and ranking tool for complex nonlinear associations, whereas GAM provides a more transparent statistical check on the main smooth relationships.

We also agree that SHAP values should not be interpreted causally. In the revised manuscript, we now state this more explicitly in both the Methods and Discussion. Specifically, we clarify that SHAP quantifies model-based feature contributions within the fitted predictive framework and therefore reflects conditional associations with ETV rather than causal effects. We have accordingly revised the relevant wording throughout the manuscript to avoid overinterpreting SHAP-based results as evidence of causation.

Corresponding modified content:

METHODS

……

Empirical Modeling Approach

To effectively reveal the complex nonlinear interactions between urban characteristics and ETV, this study combines gradient boosting decision trees (LightGBM) and the Generalized Additive Model (GAM) to address our core research question[46-49]. LightGBM is used to capture nonlinear effects and high-order interactions, quantify feature importance, and visualize local response patterns, whereas GAM serves as a complementary semi-parametric framework to assess the statistical significance, functional direction, and robustness of the identified associations.

……

To enhance the robustness of the machine learning results and address inherent challenges related to interpretability, this study integrates the Shapley Additive Explanations (SHAP) algorithm and Monte

Carlo Simulation (MCS) to systematically reveal feature importance and their nonlinear effects on ETV. SHAP was used to generate local explanations for the fitted LightGBM models by quantifying each feature's contribution to individual predictions, thereby enabling both global importance ranking and visualization of local nonlinear associations with ETV[51]. Because SHAP values are conditional on the fitted predictive model, they are interpreted here as model-based associations rather than causal effects. To assess the stability of these interpretations, we conducted 500 independent MCS runs to capture uncertainty arising from repeated model fitting. Subsequently, the SHAP values obtained from all simulations are aggregated, constructing robust distributions of feature importance and reinforcing the reliability of model interpretation[52]. Finally, to quantitatively characterize the nonlinear relationships between each feature and ETV, we employ restricted cubic spline (RCS) fitting to the aggregated SHAP-based partial dependence plots, thereby clearly delineating the marginal association patterns and trend dynamics of different variables with respect to ETV.

As a complementary validation, we further fit GAMs for ETV_Int. and ETV_Fre. to evaluate whether the nonlinear associations identified by LightGBM-SHAP remain statistically significant under a more transparent semi-parametric specification.

$$ETV = \beta_0 + \sum_{j=1}^{p} f_j(x_{ij}) + \varepsilon \tag{6}$$

where x_{ij} denotes the set of urban feature variables and background control variables. Because the relationships between explanatory variables and ETV may be strongly nonlinear, all continuous predictors enter the model as smooth functions $f_j(\cdot)$. As both ETV_Int. and ETV_Fre. are treated as annual city-level continuous outcomes, the GAMs are fitted using a Gaussian family with an identity link. To reduce the influence of extreme values and improve model stability, the strongly right-skewed variables POP_PA and EC_PA are transformed using log1p prior to model fitting. The fitted smooth terms are used to evaluate the direction, shape, and statistical significance of each association, and the estimated response curves are compared with the LightGBM-SHAP results to assess cross-model consistency.

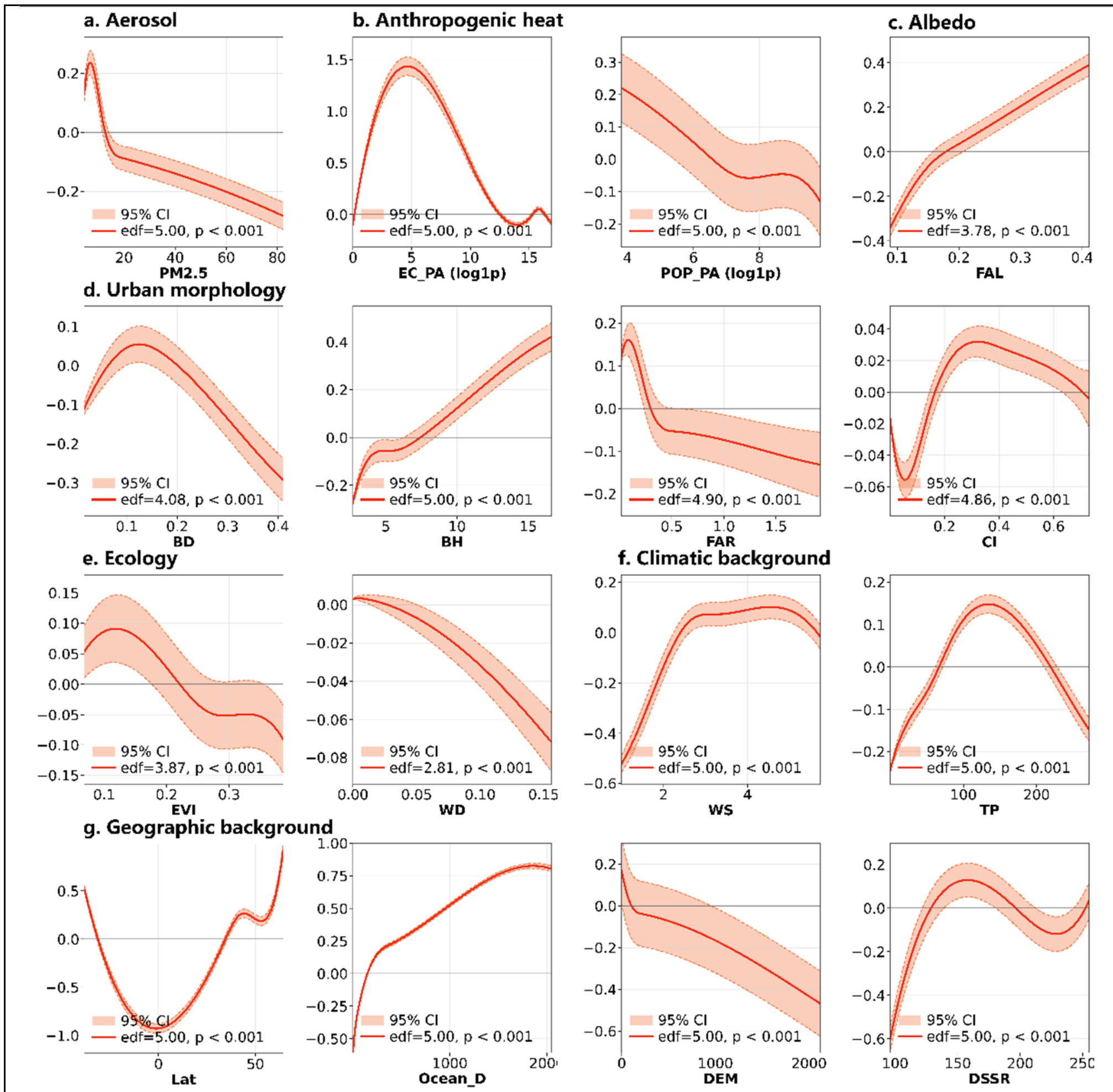

Supplementary Fig. 4 **The GAM results on ETV_Int.** Smooth effect curve and statistically significant level of each feature included in GAM on ETV_Int.(n=157,830).

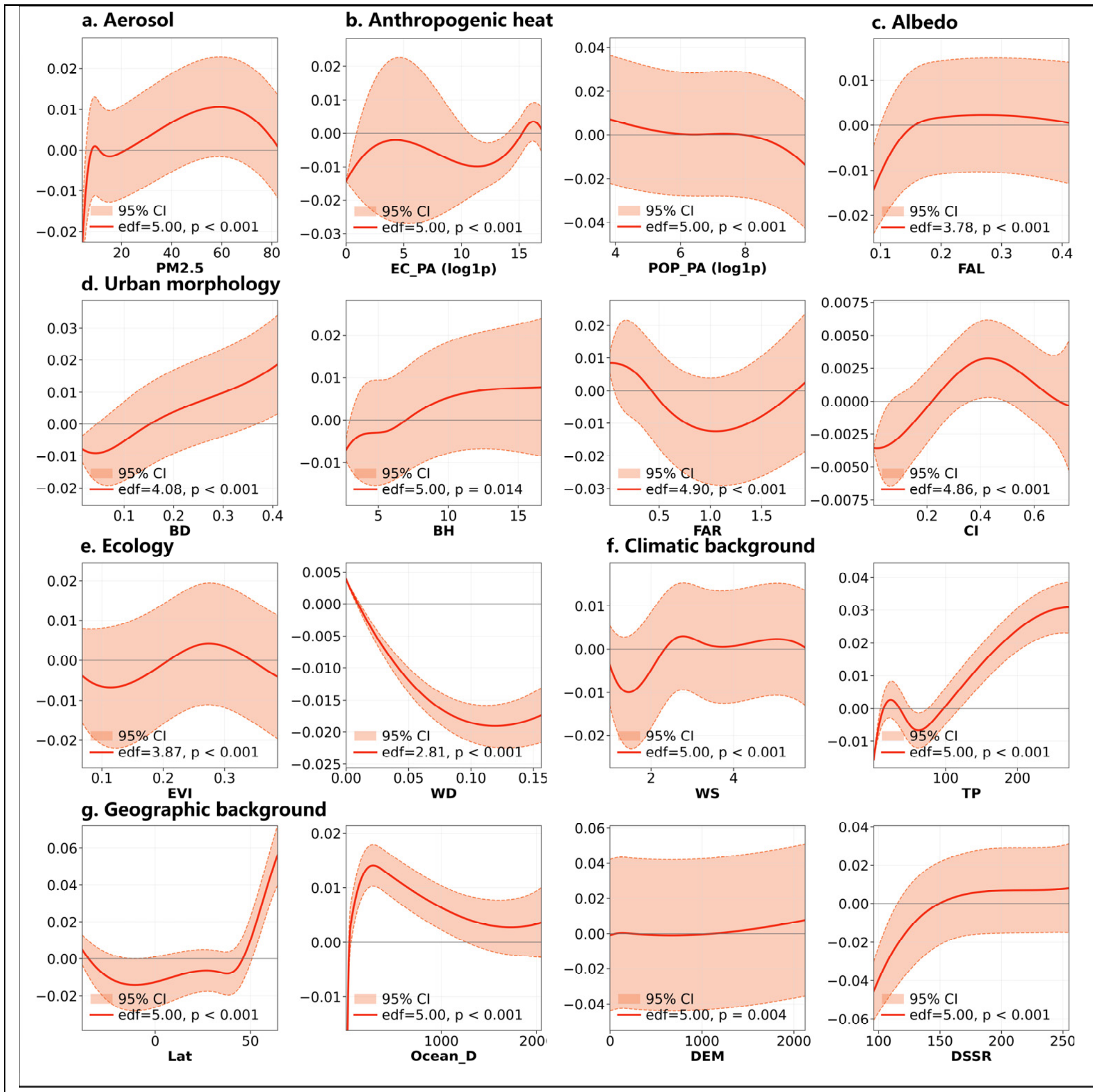

Supplementary Fig. 5 **The GAM results on ETV_Fre.** Smooth effect curve and statistically significant level of each feature included in GAM on ETV_Fre.(n=157,830).

Comment 5:

The policy implications discussed in the manuscript are relevant and potentially valuable, especially regarding aerosol control and urban design strategies. However, since the analysis is primarily based on observational data and machine learning models, the results should be interpreted as associative rather than causal. Some of the policy recommendations may therefore need to be presented more cautiously. Strengthening the discussion on causality, such as acknowledging possible reverse causality or omitted variables, would further enhance the credibility of the conclusions.

Response:

We thank you for this important comment. We fully agree that, because our analysis is based on

observational data and machine-learning models, the results should be interpreted as associative rather than causal. In the original manuscript, some policy-oriented statements may have been expressed too strongly, and we appreciate this opportunity to clarify the scope of our conclusions.

Following your suggestion, we have revised the manuscript in three ways. First, we now state more explicitly in the Methods and Discussion that the LightGBM–SHAP framework reveals model-based conditional associations rather than causal effects. Second, we have softened policy wording throughout the manuscript, replacing phrases that implied validated intervention performance with more cautious language. Third, we have strengthened the discussion of limitations by explicitly acknowledging residual confounding, omitted variable bias, and potential reverse causality. For example, $PM_{2.5}$ may covary with unobserved factors such as fuel structure, traffic intensity, building operations, or boundary-layer conditions, and some urban variables may themselves respond to thermal risk through adaptation or planning. We now note that future work should test these relationships using stronger causal designs, such as quasi-experimental evaluation, policy shocks, instrumental-variable approaches, or higher-resolution scenario-based urban climate modelling.

We believe these revisions improve the credibility of the manuscript and better align the interpretation of the policy implications with the evidentiary limits of the analysis.

Corresponding modified content:

DISCUSSION

……

Aerosols are often treated as a by-product of urban growth[31]. Using data from more than 10,000 cities, we find that aerosols emerge as the leading predictor of ETV in our models. Their relative model importance has increased over the past 15 years and is about four times that of BGI. At low to moderate concentrations, shortwave scattering reduces daytime absorption, speeds the post-event cooldown, and decreases single-event variability in size[32,33]. At higher concentrations, especially where anthropogenic heat is large, enhanced nighttime longwave re-emission raises near-surface thermal inertia[34]. Warm anomalies then decay more slowly, and ETV intensity increases. We identify a global pivot near 20 $\mu g\ m^{-3}$. Beyond this level, the damping benefit quickly fades and can even reverse. Even so, these results remain associational rather than causal, due to $PM_{2.5}$ may covary with unobserved factors such as fuel structure, traffic intensity, building operations, or boundary-layer conditions. In addition, urban form and ventilation determine how quickly heat is removed[35]. A higher floor area ratio increases effective heat capacity and dampens both warming and cooling[36]. Taller buildings and more compact layouts, particularly under background winds or frontal passages, can enhance near-surface turbulence and short-term heat exchange[16]. From a policy perspective, this links air-quality

management to thermal-volatility management. $PM_{2.5}$ control remains a no-regret option because it yields immediate health benefits and may also help moderate ETV in polluted, energy-intensive settings, especially when paired with albedo management and ventilation-sensitive design.

BGI remains consistently negatively associated with ETV, but its contribution is smaller and more context-dependent than that of its well-established role in reducing mean heat exposure[16,22,26,37]. This suggests that ecological infrastructure can buffer thermal volatility, but its effectiveness depends strongly on surrounding urban form, ventilation, and climatic background. Morphology-related variables also show mixed signs, highlighting trade-offs among heat storage, roughness, shading, and turbulent exchange[16,37]. The policy implication is therefore a place-based portfolio approach rather than a one-size-fits-all intervention logic. High-pollution, high-density cities may benefit most from integrated packages combining emissions reduction, reflective-surface or envelope upgrades, ventilation corridors, and connected blue–green networks. Cities with already strong ecological bases may gain more from protecting continuity, reducing fragmentation, and targeting heat-vulnerable districts. Rapidly urbanizing cities may reduce future ETV lock-in by embedding ecological continuity, street ventilation, and surface-property management early in land-use planning[38,39].

More broadly, the value of this analysis lies less in prescribing a universal intervention package than in informing policy sequencing and regional prioritization. In South Asia and East Asia, where heavy pollution is associated with a greater risk of fluctuations, air-quality control should be paired with albedo management and ventilation-sensitive design. In dense urban corridors of the Middle East and the United States, morphology-sensitive retrofits and ventilation corridors may be more effective than green expansion alone. By contrast, cities in Western Europe, Eastern Europe, and the eastern United States may gain more from protecting and connecting existing blue–green networks. Because the present study is observational and retrospective, these directions should be interpreted as management-relevant rather than as causal or scenario-tested prescriptions.

Finally, we acknowledge several limitations and outline the next steps. Our analysis is designed for the city scale. Because ETV is derived from gridded daily near-surface air temperature, the dataset cannot fully resolve neighborhood- or canopy-scale thermal heterogeneity. Our conclusions should therefore be interpreted as city-level patterns rather than direct evidence of street-scale urban heat processes. Second, the next-step research should employ counterfactual designs or instrumental-variable strategies to disentangle the roles of emissions, morphology, and meteorology, clarify causal effects, and strengthen threshold estimates for the urban-process variable. Finally, future research should include future projections from CMIP6 models to assess trends in ETV across different scenarios. Despite these limitations, our harmonized analysis of 10,522 cities shows that urbanization is reshaping the intensity and frequency structure of temperature risk. By disentangling how urbanization processes interact with the climatic background, we outline a practical path to steer cities out of the high-frequency, large-amplitude tail of risk and back into a safer operating space.

METHODS

……

Empirical Modeling Approach

To effectively reveal the complex nonlinear interactions between urban characteristics and ETV, this study combines gradient boosting decision trees (LightGBM) and the Generalized Additive Model (GAM) to address our core research question[46-49]. LightGBM is used to capture nonlinear effects and high-order interactions, quantify feature importance, and visualize local response patterns, whereas GAM serves as a complementary semi-parametric framework to assess the statistical significance, functional direction, and robustness of the identified associations.

……

To enhance the robustness of the machine learning results and address inherent challenges related to interpretability, this study integrates the Shapley Additive Explanations (SHAP) algorithm and Monte Carlo Simulation (MCS) to systematically reveal feature importance and their nonlinear effects on ETV. SHAP was used to generate local explanations for the fitted LightGBM models by quantifying each feature's contribution to individual predictions, thereby enabling both global importance ranking and visualization of local nonlinear associations with ETV[51]. Because SHAP values are conditional on the fitted predictive model, they are interpreted here as model-based associations rather than causal effects. To assess the stability of these interpretations, we conducted 500 independent MCS runs to capture uncertainty arising from repeated model fitting. Subsequently, the SHAP values obtained from all simulations are aggregated, constructing robust distributions of feature importance and reinforcing the reliability of model interpretation[52]. Finally, to quantitatively characterize the nonlinear relationships between each feature and ETV, we employ restricted cubic spline (RCS) fitting to the aggregated SHAP-based partial dependence plots, thereby clearly delineating the marginal association patterns and trend dynamics of different variables with respect to ETV.

__Comment 6:__

It would be helpful if the authors could provide a README or clear documentation to facilitate code usability and reproducibility.

__Response:__

Thank you for this helpful suggestion. We agree that clear documentation is important for improving code usability and reproducibility. In response, we have added a dedicated README file to the code repository and provided a sample dataset to help users understand the data structure and run the analytical workflow.

Responses

No.: NCOMMS-25-104270A

Urbanization processes widen global divergence in extreme temperature variability

Thank you for the comments and suggestions regarding our manuscript entitled "Urbanization processes widen global divergence in extreme temperature variability" (No. NCOMMS-25-104270A). These comments are valuable in revising our paper and improving its quality.

The amendments in the paper, along with a point-by-point response to the comments and suggestions, are as follows. We believe that these revisions have substantially improved the manuscript's rigor, transparency, and interpretability, and we are grateful for your guidance in strengthening it. We trust that the revised paper meets your expectations.

<u>Responses to Reviewer #1's comments:</u>

The authors have satisfactorily addressed my previous concerns and made substantial revisions to the manuscript. However, I believe that two issues still require clarification before publication.

<u>Comment 1:</u>

The study argues that the newly proposed extreme temperature variability metric captures hazard-related information that is not adequately reflected by conventional temperature-extreme metrics. However, the manuscript does not clearly articulate the specific advantages of extreme temperature variability, nor does it explicitly explain which limitations of existing metrics it overcomes.

<u>Response:</u>

We thank you for this important comment. In the revised manuscript, we have strengthened the conceptual framing of ETV and clarified its distinct advantages. Conventional temperature-extreme metrics are primarily designed to characterize the occurrence, intensity, or duration of sustained thermal extremes. These metrics are highly informative for chronic warming and heatwave exposure, but they are less well suited to capturing abrupt day-to-day temperature swings. This is important because rapid temperature swings can generate physiological, infrastructural, and energy-system stress even when conventional heat metrics do not indicate persistent extreme conditions.

We therefore regard ETV not as a replacement for existing temperature-extreme metrics, but as a complementary hazard dimension. Its main advantage is that it captures the structure of thermal fluctuation itself rather than only the exceedance of a high-temperature threshold. In this sense, ETV addresses a limitation of conventional metrics that summarize heat burden mainly through absolute thresholds, annual maxima, or event duration, while providing limited information on the volatility and recovery structure of thermal exposure. To clarify, we have revised the Introduction to explicitly state that ETV is intended to capture a distinct, weather-scale dimension of climate risk.

**Corresponding modified content:**
**INTRODUCTION**<br>**……**<br>Urban areas sit at the frontline of climate risk. Cities concentrate people, infrastructure, and economic

activity, and their distinct microclimates (for example, urban heat islands) can amplify heat exposure[8]. Observations show that, in recent decades, urban centers have warmed about 29% faster than nearby rural areas[9]. Most studies emphasize macro-scale indicators, such as the differences in mean temperature between urban and suburban areas and the frequency and intensity of extreme events. In contrast, knowledge of urban temperature variability, including abrupt day-to-day fluctuations and changes, remains limited[6,7,10]. Conventional temperature-extreme metrics are primarily designed to characterize exceedance, duration, or annual maxima of sustained thermal extremes. They are therefore highly informative for chronic warming and heatwave exposure, but less suited to capturing abrupt day-to-day thermal volatility and the cumulative load generated by repeated temperature swings. ETV is intended to complement, rather than replace, these metrics by quantifying the structure of thermal fluctuation itself, thereby identifying risk associated with rapid temperature changes even where persistent extreme conditions are less pronounced. Recent work also shows that land use change, such as deforestation, can increase daily temperature variability by up to 20% in certain regions, resulting in more frequent and rapid temperature swings, both warm and cold. However, whether the dense and thermally complex fabric of cities dampens or magnifies such abrupt variability is still unclear and calls for direct investigation.

Comment 2:

Although the authors claim that combining LightGBM and GAM provides significant advantages for identifying nonlinear relationships between urban characteristics and extreme temperature variability, the approach is fundamentally based on statistical and machine learning models. Therefore, it cannot fully escape the inherent limitations associated with black box modelling. The authors should explicitly acknowledge these limitations and discuss them in more detail. Additionally, outlining potential physics- or process-based modelling frameworks that could address these limitations in future research and provide a more mechanistic understanding of the observed relationships would be valuable.

Response:

We thank you for this important comment. We agree that combining LightGBM with GAM improves the detection and interpretation of nonlinear associations but does not eliminate the inherent limitations of data-driven modeling. We have therefore revised the manuscript to acknowledge several specific limitations. The inferred associations depend on the variables included in the models and may remain affected by omitted-variable bias, residual confounding, and correlations among predictors.

Following your suggestion, we have also expanded the Discussion to outline a process-based research

framework. Future work could couple high-resolution regional atmospheric models with urban canopy and building-energy schemes to explicitly represent radiation trapping, heat storage, anthropogenic heat, ventilation, vegetation, and water. Physics-informed or hybrid process－machine-learning models may provide an additional pathway by combining global predictive capacity with stronger physical constraints.

**Corresponding modified content:**
**DISCUSSION**<br>**……**<br>Finally, we acknowledge several limitations and outline the next steps. Our analysis is designed for the city scale. First, although the combined LightGBM–GAM framework improves the detection and interpretation of nonlinear associations, it does not fully overcome the limitations of data-driven modeling. The estimated relationships may also be affected by omitted variables, residual confounding, and limited extrapolation beyond the observed climatic and urban conditions. Future work should couple high-resolution atmospheric models with urban canopy, building-energy, and aerosol–radiation–boundary-layer schemes to explicitly resolve radiation, heat storage, anthropogenic heat, turbulent exchange, and hydrological buffering. Controlled factorial simulations under matched synoptic conditions, validated against station, flux-tower, and remote-sensing observations, would help determine whether the associations identified here reflect causal physical pathways. Hybrid physics-informed machine-learning approaches may further combine global scalability with stronger process constraints. Second, because ETV is derived from gridded daily near-surface air temperature, the dataset cannot fully resolve neighborhood- or canopy-scale thermal heterogeneity. Our conclusions should therefore be interpreted as city-level patterns rather than direct evidence of street-scale urban heat processes. Finally, future research should include future projections from CMIP6 models to assess trends in ETV across different scenarios. Despite these limitations, our harmonized analysis of 10,522 cities shows that urbanization is reshaping the intensity and frequency structure of temperature risk. By disentangling how urbanization processes interact with the climatic background, we outline a practical path to steer cities out of the high-frequency, large-amplitude tail of risk and back into a safer operating space.
